# Modular supramolecular dimerization of optically tunable extended aryl viologens†
†Electronic supplementary information (ESI) available. See DOI: 10.1039/c9sc03057c


**DOI:** 10.1039/c9sc03057c

**Published:** 2019-08-12

**Authors:** Magdalena Olesińska, Guanglu Wu, Silvia Gómez-Coca, Daniel Antón-García, Istvan Szabó, Edina Rosta, Oren A. Scherman

**Affiliations:** a Melville Laboratory for Polymer Synthesis , Department of Chemistry , University of Cambridge , Lensfield Road , Cambridge , CB2 1EW , UK . Email: oas23@cam.ac.uk; b Department of Chemistry , King's College London , 7 Trinity Street , London , SE1 1DB , UK; c Department of Chemistry , University of Cambridge , Lensfield Road , Cambridge CB2 1EW , UK

## Abstract

Cucurbit[8]uril (CB[8]) mediated assembly of extended aryl viologens (EVs) into optically tunable dimers is reported for the first time. We show that the modular design and synthesis of a new class of π-conjugated viologen derivatives with rigid aromatic or heteroaromatic bridging units as well as electron donating molecular recognition motifs enable their self-assembly into 2 : 2 complexes with CB[8]. The quantitative dimerization process involving these two molecular components in an aqueous solution enables excimer-like interactions between closely packed charged guests giving rise to distinct spectroscopic behavior. The nature of these dimers (CB[8]_2_·(EV[X]R)_2_) in the ground and excited states was characterized by NMR, isothermal titration calorimetry, and steady-state spectroscopic measurements.

## Abstract 2

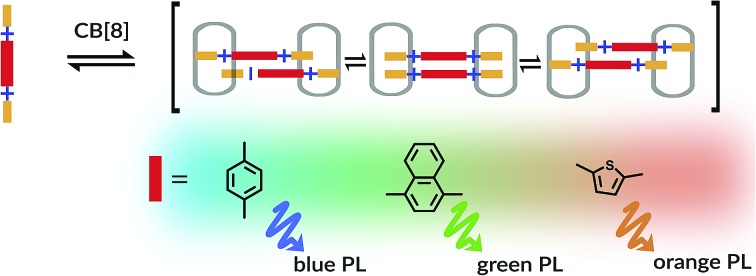
Cucurbit[8]uril (CB[8]) mediated assembly of extended aryl viologens (EVs) into optically tunable dimers is reported for the first time.

## 

Achieving precise control over the spatial arrangement of organic photoemissive molecules is of interest in the area of photovoltaic device construction, light-emitting diodes,^1^ sensing and bio-imaging.^2^ Both covalent and non-covalent interactions between fluorescent molecules have been used in order to optimize their best optical properties. Recently, a number of examples have been reported in the literature, which detail control over the emissive states of discrete organic molecules or complexes including the luminescence of molecular rotors based on aggregation induced emission,^3^ generation of excimers with modifications in covalently bound chromophores^4^–^6^ and H- and J-aggregate formation on account of encapsulation within supramolecular macrocycles or self-assembled cages.^7^–^16^ Nevertheless, these systems are synthetically challenging and do not provide a modular approach for tuning the optoelectronic properties.

Cucurbit[*n*]urils (CB[*n*]) are a family of macrocyclic host molecules often used in synthetic and materials sciences on account of their variety of sizes and capability to complex different types of guest molecules in an aqueous environment.^17^ In particular, cucurbit[8]uril (CB[8]) is of interest due to its ability to host two guest moieties simultaneously. We previously described the binding of CB[8] with symmetric diaryl viologens bearing electron-donating groups (Fig. 1a) in a 2 : 2 binding fashion, representing an understudied binding mode in the literature.^18^ The formation of such 2 : 2 complexes led to interesting changes of their optical properties. We observed a uniform red-shift in the absorption of the 2 : 2 complexes relative to their monomeric UV/vis spectra, and in a few specific cases (R = Me or OMe) an intense fluorescence emission typical of J-aggregates (see Fig. S75†).^18^ A similar observation of enhanced photoluminescence for 2 : 2 complexes of CB[8] and viologen derivatives was recently described.^19^,^20^

**Fig. 1:**
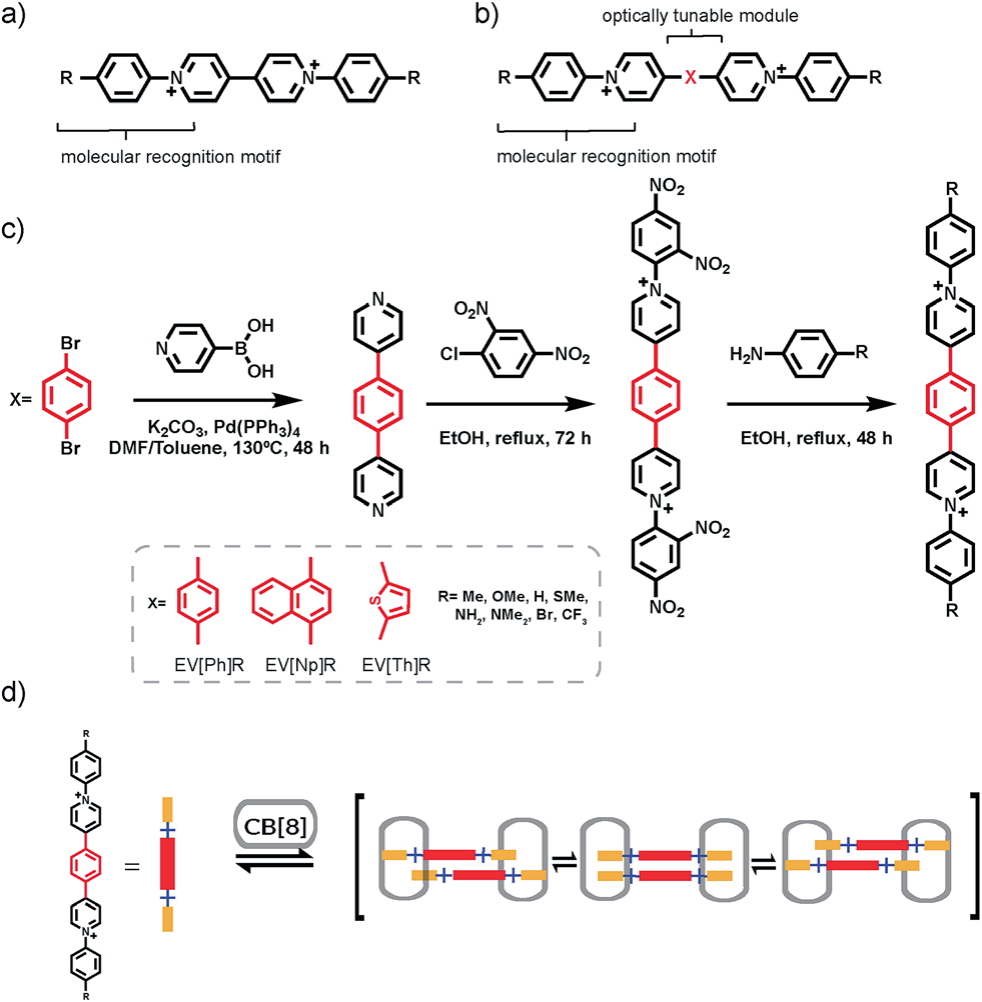
(a) Aryl viologen used in previous work with a molecular recognition motif (Ph-R) for CB[8] binding. (b) The extended aryl viologen design principle, with optically tunable (X) and molecular recognition (Ph-R) modules used in this work. (c) General synthetic route towards extended aryl viologens (EVs). (d) Scheme of 2 : 2 CB[8]_2_·(EV[Ph]R)_2_ complex formation for R groups with an electron donating character. Cl^–^ counterions have been omitted for clarity.

In order to take advantage of strict 2 : 2 complex formation through the CB[8] binding motif we explored the introduction of an extended π-bridging unit between the two pyridyl groups as shown in Fig. 1b. Symmetric extended aryl viologen (EV) derivatives with various conjugated cores *i.e.* phenyl (EV[Ph]), naphthyl (EV[Np]) and thiophene (EV[Th]) were synthesized in a three-step procedure, as depicted in Fig. 1c. Bipyridine derivatives were synthesized through Suzuki coupling of pyridine 4-boronic acid and the corresponding dibromo species. These bipyridine derivatives were then subjected to a two-step Zincke reaction with the formation and isolation of the symmetric Zincke salt. Subsequent reaction of the symmetric Zincke salt with appropriate amines yielded various extended aryl viologens, EV[Ph]R, EV[Np]R and EV[Th]R (see the ESI†). The directing capability of the CB[8] macrocycle around the aryl pyridyl groups facilitate π–π stacking of the linker units and control the spatial arrangement of the EV monomers. The donor–acceptor character of molecules coupled in close proximity imposed by the CB[8] macrocycles leads to positive or negative coulombic interactions.^21^ This results in competition between a favorable electrostatic interaction and π–π stacking that has a strong effect on the photophysical performance of the formed dimers.^22^

We report here the extent of inter-chromophoric interactions in the ground and excited states based on their steady-state electronic transitions and fluorescence lifetimes as a function of their binding with CB[8]. We hypothesized that the emissive excited state properties of the dimeric complexes CB[8]_2_·(EV[X]R)_2_ would not only be influenced by the host-guest interactions in their ground state but also in their excited state as an excimer-like complex. Previous observations have been made for molecular packing of naphthalene, anthracene and phenanthrene under pressure or in the solid state with a red shift of the energies of their electronic transitions (lower energy), and with broadened and featureless spectral bands.^23^ In the system used in this study, complexation inside the confined space of the CB[8] macrocycles results in distinct π-stacking in solution. It was recently shown that the distance between molecules affects charge transfer interactions and excimer formation.^4^ However, recent reports by Wu *et al.* and many other reports are focused mostly on covalent dimers where the dimeric units are not free to rotate or rearrange.^2^,^4^,^24^–^26^ Given that the supramolecular dimeric systems reported here are dynamic, we reasoned that upon excitation, the two guest molecules are still able to rearrange in their excited states within the complex and reach the most stable excited state geometry.

Following synthesis and characterization, the binding properties of these new extended aryl viologens with CB[8] were probed using spectroscopic and thermodynamic techniques, including ^1^H NMR and isothermal titration calorimetry (ITC). Typical NMR experiments are carried out at 100 uM at room temperature where the 2 : 2 complexes form instantly. Guest complexation with the hydrophobic cavity of CB[8] typically results in an upfield shift of the encapsulated protons and a downfield shift of protons in close proximity to the carbonyl portals as shown in Fig. 2. Interestingly, titration of EV[Ph]Me with a solution of CB[8] led to the broadening of the guest signals (Fig. 2a and S52†). The proton integration ratio of EV[Ph]Me mixed with CB[8] suggests the formation of a dynamic 2 : 2 host-guest complex (see the ESI Fig. S49†). The broad NMR peaks are attributed to the unencumbered aromatic linker unit, in this case a phenyl ring, which is small enough to allow the CB[8] macrocycles to shuttle back and forth in the 2 : 2 complex. Augmenting the bridging unit from Ph to Np results in a complex that appears to be less dynamic as the bulkier Np dimer retards the rate of CB shuttling. Upon titration of the bulkier EV[Np]Me molecule with a CB[8] solution, a 0.5 ppm upfield shift of the methyl proton *H*_a_ was observed (Fig. 2b and S56†) at a 1 : 1 ratio, indicative of encapsulation or partial encapsulation inside the cavity of CB[8]. The incorporation of a heteroatom into the bridging species was also possible resulting in an extended viologen bearing a thiophene unit, EV[Th]Me. Analogous proton shifts of the tolyl group and thiophene core were observed (Fig. 2c and S67†) upon 2 : 2 complex formation with EV[Th]Me; a pronounced broadening of peaks in the CB[8]:EV[Th]Me complex suggests an increase of dynamics in such a system.

**Fig. 2:**
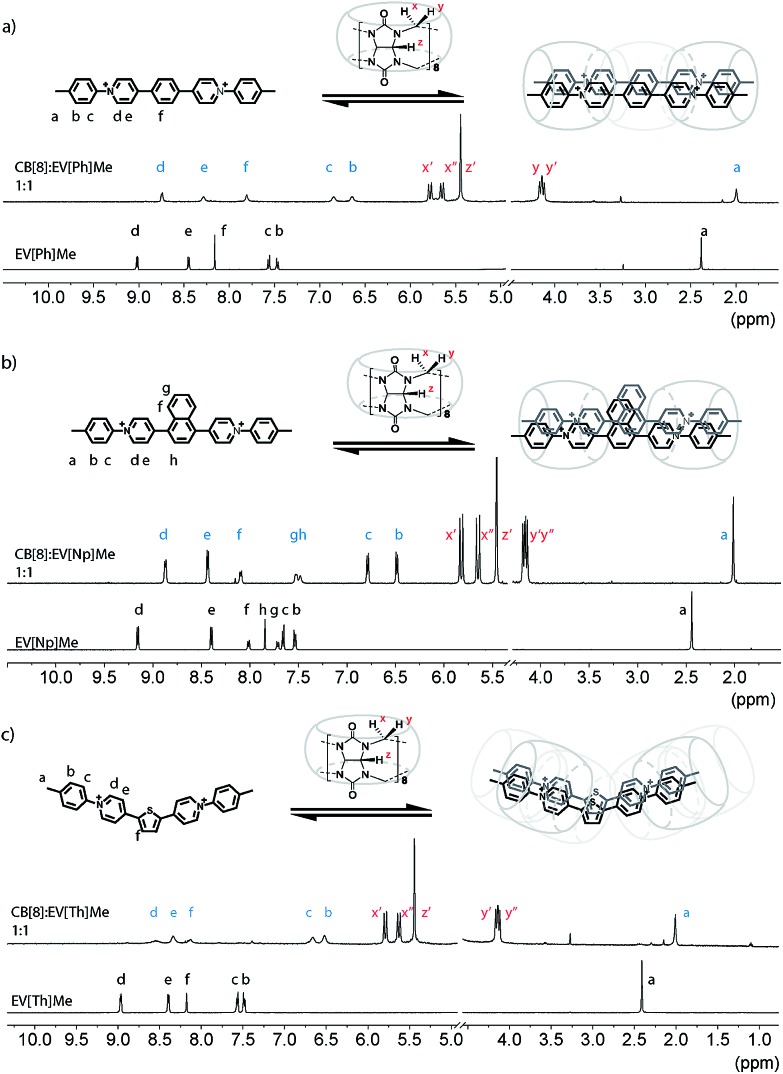
^1^H NMR spectra (500 MHz) of (a) EV[Ph]Me, (b) EV[Np]Me, and (c) EV[Th]Me and their complexes with 1 eq. of CB[8] (*D*_2_O, 298 K). Cl^–^ counterions have been omitted for clarity.

Regardless of the bridging unit, all of the EVs studied revealed a 2 : 2 binding motif, which could be confirmed from the unique splitting pattern of the NMR peaks corresponding to the asymmetric magnetic field for methine protons at the CB[8] portals.^18^ The size and geometric orientation of the aromatic bridging unit, however, may have an effect on the shuttling dynamics and relative location of the CB[8] macrocycle in the complex. Moreover, bridging units with a larger aromatic surface area, such as naphthalene, lead to a less dynamic complex that may benefit from stronger π–π interactions.

The EV-CB[8] complexes were further evaluated using ITC. Our recent work showed that the formation of CB[8] complexes is readily characterized by the magnitude of the overall binding enthalpy (Δ*H*), typically around –70 kJ mol^–1^.^18^ The enthalpy data obtained for all the EVs bearing electron donating groups were found to range from –90 kJ mol^–1^ to –70 kJ mol^–1^ suggesting 2 : 2 binding with CB[8] (see the ESI†) in agreement with ^1^H NMR (Fig. 2). Additionally, the binding constants (*K*_a_) for the EV and CB[8] complexes were found to be within 5.5 × 10^6^ M^–1^ to 3.8 × 10^7^ M^–1^, indicating strong host–guest binding. Importantly, when EV[Np]R bearing electron withdrawing groups (R = CF_3_ and Br) was mixed with a stoichiometric amount of CB[8], the complexes formed were not found to bind in a 2 : 2 manner, but rather yielded 1 : 1 or n:n complexes as confirmed by NMR and ITC measurements (see the ESI†).

Complexation inside the CB[8] cavity affects the local environment of extended viologens, which in turn alters their optical properties. It has been reported that encapsulation in CB[8] causes a red shift in the absorption and emission bands, as well as an increase in the fluorescence quantum yield (*Φ*_*PL*_) and fluorescence lifetime (*τ*).^10^,^27^–^30^ For the extended viologens reported here, we expect the structural differences within this series (increasing the π-surface area and inclusion of a heteroaromatic group in the bridging unit) to augment both complexation with CB[8] and their respective electronic transition energies. Normalized steady-state absorption and emission spectra of the three EVs studied in the absence and presence of 1 eq. of CB[8] are shown in Fig. 3. The spectral properties are summarized in Table 1.

**Fig. 3:**
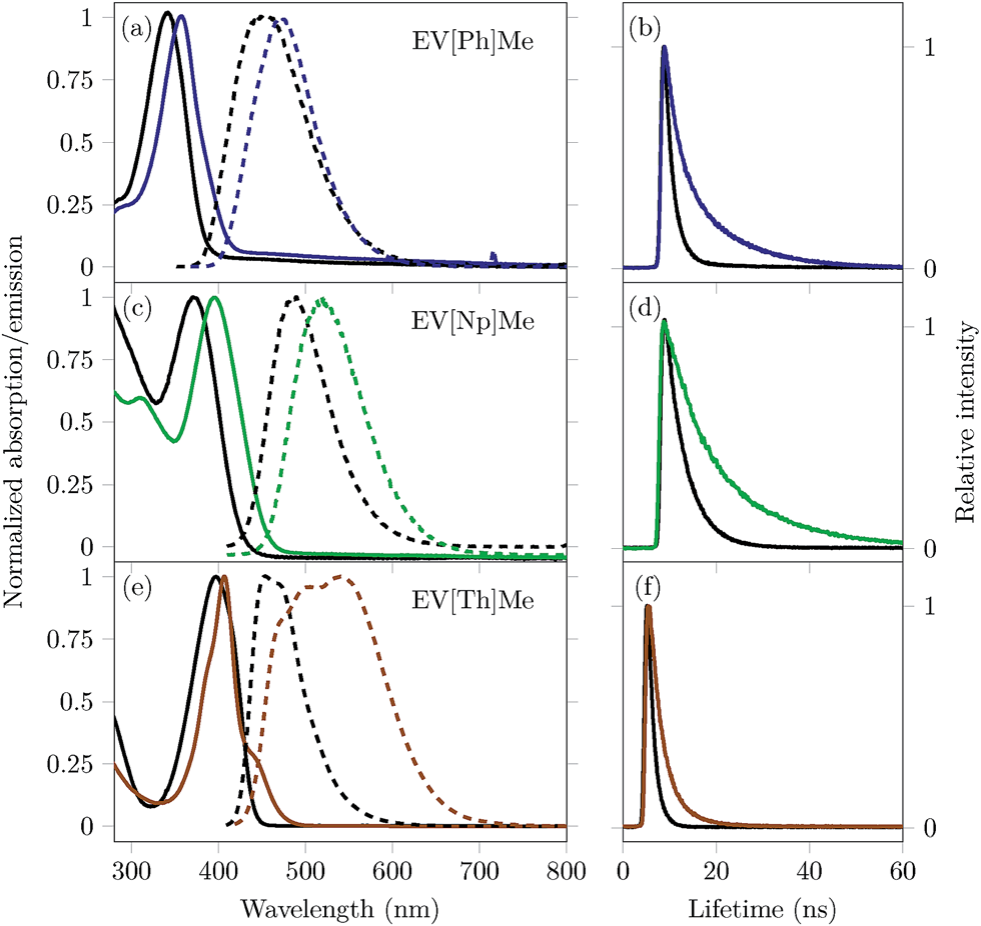
Left graph: Normalized absorption (solid lines) and fluorescence (dashed lines) spectra of extended viologens (a) EV[Ph]Me, (c) EV[Np]Me, and (e) EV[Th]Me (black lines) and their complexes with 1 eq. of CB[8] (colored lines); Right graph: Time-correlated single photon counting decay profiles for (b)EV[Ph]Me (black line) and EV[Ph]Me:CB[8] in a 2 : 2 mixture (blue line), (d) EV[Np]Me (black line) and EV[Np]Me:CB[8] in a 2 : 2 mixture (green line), and (f) EV[Th]Me (black line) and EV[Th]Me:CB[8] in a 2 : 2 mixture (orange line). All measurements were performed in water, with a guest concentration of 10 μM.

**Table 1:** Steady-state spectral data of EV[X]Me in water

Guest	Host	*λ* max abs [nm], (*ε*^*a*^ [M^–1^ cm^–1^])	*λ* max em [nm]	Stokes shift [nm], [Δ*υ*(cm^–1^)]	*Φ*_PL_ ^*b*^	*τ*_S1_ ^*c*^ (ns)	*k*_rad_ ^*d*^ (×10^8^ s^–1^)	*k*_nrad_ ^*e*^ (×10^8^ s^–1^)
EV[Ph]Me	—	343 (24 400)	456	113 (7225)	0.65	1.5	4.33	2.33
EV[Ph]Me	CB[8]	357 (37 200)	472	115 (6824)	0.92	2.5 (23%) 10.9(77%)	0.84	0.07
EV[Np]Me	—	371 (15 700)	487	116 (6348)	0.68	4.2	1.62	0.76
EV[Np]Me	CB[8]	396 (24 900)	518	122 (5947)	0.58	6.8 (31%) 17.1(69%)	0.33	0.24
EV[Th]Me	—	397 (51 740)	453	56 (3114)	0.62	1.3	4.77	2.93
EV[Th]Me	CB[8]	407 (77 700)	544	137 (6187)	0.07	2.7 (82%) 6.7 (18%)	0.26	3.44

^*a*^The molar absorptivities at the wavelength *λ*maxabs.

^*b*^Fluorescence quantum yield at r.t.

^*c*^Fluorescence lifetime.

^*d*^Fluorescence radiative rate estimated using the equation *k*_rad_ = *Φ*_PL_/*τ*_S1_.

^*e*^ *k*_nrad_= (1/*τ*_S1_) – *k*_rad_.

UV/vis and emission spectra of all three EV[X]Me reveal a red shift in the absorption and fluorescence emission upon increasing the aromatic conjugation or incorporating a heteroatom into the core (see Fig. 3).

The ground-state UV/vis absorption spectra of EV[Ph]Me (solid black line, Fig. 3a) in water show a maximum absorption band at *λ*_abs_ = 343 nm (*ε* = 24 400 M^–1^ cm^–1^) and a broad, featureless emission band with a maximum at *λ*_em_ = 456 nm (*Φ*_*PL*_ = 0.65). Upon mixing with 1 eq. of CB[8], bathochromic shifts of over 10 nm in the absorption and emission spectra are observed, as well as strong enhancement in the fluorescence quantum yield (*Φ*_*PL*_ = 0.92). Similarly, for EV[Np]Me (solid black line, Fig. 3c) the ground-state UV/vis absorption spectra are characterized by a maximum absorption band at *λ*_abs_ = 371 nm (*ε* = 15 700 M^–1^ cm^–1^) and an emission band at *λ*_em_ = 487 nm (*Φ*_*PL*_ = 0.68). Upon mixing with 1 eq. of CB[8], a 25 nm red-shift in the absorption spectra and a 31 nm red-shift in the emission spectra are observed, as well as a small decrease in the fluorescence quantum yield (*Φ*_*PL*_ = 0.58). The UV/vis absorption spectra of EV[Th]Me (solid black line, Fig. 3e) in water show a maximum in the absorption band at *λ*_abs_ = 397 nm (*ε* = 51 740 M^–1^ cm^–1^) and a maximum in the emission band at *λ*_em_ = 453 nm (*Φ*_*PL*_ = 0.62). Upon mixing with 1 eq. of CB[8], a 10 nm red-shift in the absorption spectra and a 91 nm red-shift in the emission spectra are observed.

Unlike the cases of EV[Ph]Me and EV[Np]Me, a huge decrease in the fluorescence quantum yield is recorded for EV[Th]Me(*Φ*_*PL*_ = 0.07). The emission spectrum observed for EV[Th]Me complexed with CB[8] has characteristics of typical excimer-like species; here, where 2 : 2 complex formation is evident, two thiophene bridging moieties are electronically coupled in the excited state. Correspondingly, monomeric EV[Th]Me and de-aggregated 2 : 1 complexes with CB[7] have high quantum yields of 0.62 and 0.67, respectively (see the ESI†). The Stokes shift of 122 nm (5947 cm^–1^) for the CB[8]_2_·(EV[Np]Me)_2_ complex and 137 nm (6187 cm^–1^) for CB[8]_2_·(EV[Th]Me)_2_ complex indicates that the dipole moments of the photoexcited states have been changed significantly, suggesting the formation of intramolecular charge transfer states. Broad, unstructured red-shifted emission is characteristic of charge–transfer transitions as highlighted by Wasielewski and co-workers.^4^ Moreover, these are also characteristic steady-state parameters exhibited by excimers or excimer-like species.

Time-correlated single photon counting (TCSPC) of the EVs alone and in the presence of 1 eq. of CB[8] was carried out to gain insight into the possible EV–EV interactions and their dynamics in the excited state. Fig. 3 highlights the emission decay profiles, and the corresponding lifetime data are given in Table 1. Following the excitation of EV[Ph]Me at its *λ*maxabs, the major emissive component decays with a time constant of 1.5 ns. Upon encapsulation within the CB[8] cavity, long-lived fluorescence was observed with a decay time of 10.9 ns. Additionally, EV[X]Me with an increased π-surface area showed longer fluorescence lifetimes, decaying with a time constant of 17 ns. The long-lived emission of these complexes indicates the existence of a stable, long-lived excited state.

It has been reported that the inclusion of dye molecules inside CBs leads to a decrease in *k*_nrad_ on account of the mechanical protection from quenchers present in solution.^29^,^31^,^32^ However, the (CB[8]_2_·(EV[X]Me)_2_) complexes for X = Ph and Np give rise to a notable decrease in both *k*_rad_ and *k*_nrad_. In the case of the (CB[8]_2_·(EV[Th]Me)_2_) complex we observed lower radiative but higher non-radiative decay rates in comparison to free guests (see Table 1). On one hand, such observations could be explained by a high non-radiative decay due to inter-system crossing or internal conversion within this complex. On the other hand the photophysical processes occurring within the 2 : 2 complex clearly depend on the core structure, molecular geometry, and the way the stacking occurs within the distinct dimeric complex.^21^,^33^

To gain more insight into the dynamic, static and spectroscopic properties of the EV-CB[8] dimeric complexes, molecular dynamics (MD) simulations were performed, followed by quantum chemical calculations. Molecular interactions in these MD simulations include both coulombic repulsion and π–π attraction between the pairs of EVs, leading to the observed dynamic behavior of the complexes (see the ESI† and simulation movies). The simulations suggest a more dynamic behavior of EV[Ph]Me compared to EV[Np]Me or EV[Th]Me, in agreement with experimental observations. The MD structures were used as initial starting points to carry out further quantum chemical calculations. We used a recently developed semiempirical method, GFN-xTB,^34^ that has been demonstrated to successfully model supramolecular complexes.^35^ Furthermore, it can also be combined with the sTDA approximation for the calculation of UV spectra (for details see the ESI†).^36^,^37^ To explore different aggregates at this level of theory, we first clustered the structures from the MD simulation trajectories and selected representative geometries as starting points for the geometry optimization. The most stable optimized molecular structures of EV[Ph]Me, EV[Np]Me, and EV[Th]Me complexed with CB[8] in a 2 : 2 manner using TB-DFT are shown in Fig. 4, together with their characteristic geometric parameters in Table S4.† In the optimized structures, the CB[8]-complexed guest molecules have short π–π distances between each other. Furthermore, they also show slightly shorter distances between the parallel rings of the guest molecules. The obtained optimized geometries suggest a stronger effect of π–π interactions and a smaller coulombic repulsion giving rise to final structures with stabilized head-to-head geometries compared with head-to-tail arrangements.

**Fig. 4:**
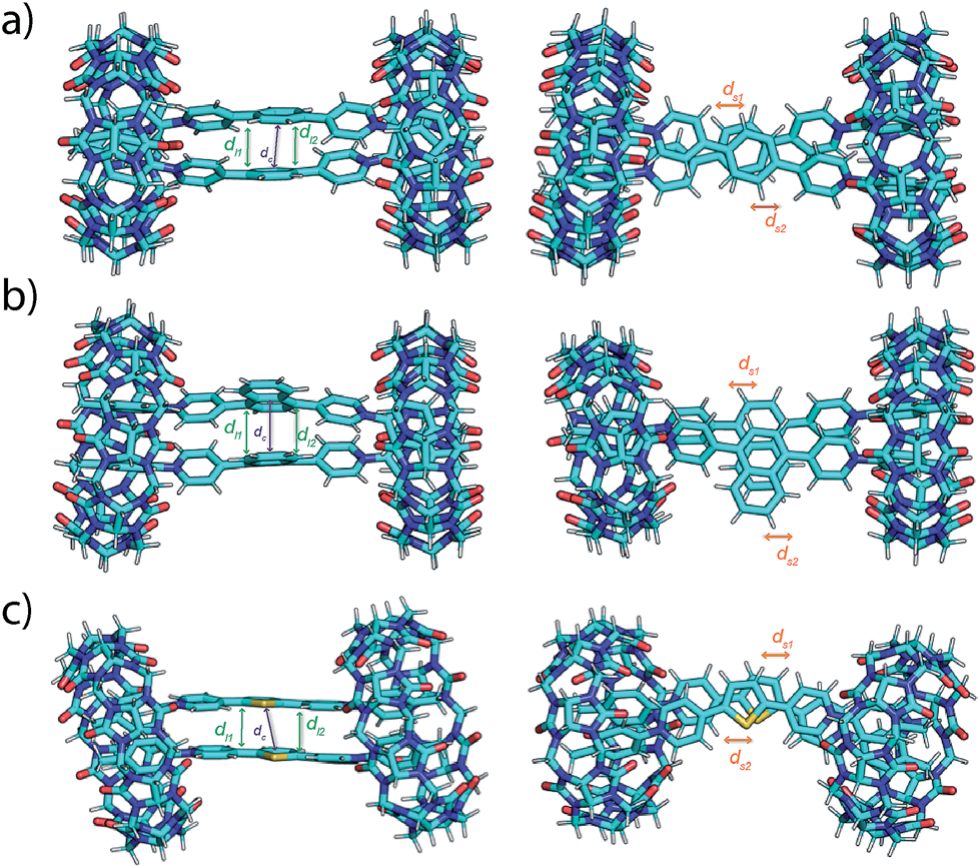
Front and top view of GFN2-xTB DFT-computed ground-state geometries of 2 : 2 complexes of CB[8] with (a) EV[Ph]Me, (b) EV[Np]Me, and (c) EV[Th]Me. In the figures, centroid distances (*d*_c_, purple), distances between parallel planes (*d*_l1_ and *d*_l2_, green) and intercentroid shifts (*d*_s1_ and *d*_s2_, orange) between the rings are also indicated by arrows (see the ESI†).

The calculated spectroscopic properties of the EVs show good agreement with the experimental absorption spectra, displaying red shifts in absorption upon increasing the aromatic conjugation or incorporation of a heteroatom (see Fig. S83†). In the case of the EV-CB[8] dimeric complexes, no shift or a small blue shift in the calculated absorption spectra is observed (see Fig. S84†). This suggests the enhanced stability of the H-stacked instead of J-stacked guest dimers in the semiempirical calculations, which is likely due to using an implicit solvent in the quantum chemical calculations. Accordingly, MD structures show more balanced interactions with considerably longer intercentroid distances (*d*_c_) and shifts (*d*_s1_ and *d*_s2_) between the rings compared with the geometry optimized structures (Fig. S80–82†). The presence of explicit water interacting with both the EVs and CB[8] therefore might play a role in increasing the stability of the J-type orientation.

We have described the synthesis of a new family of extended viologen compounds with increasing π-surfaces between the two pyridinium units. The binding properties of this family of molecules with CB[8] are assessed through ^1^H NMR and ITC measurements and all three EVs result in the formation of explicit 2 : 2 host-guest complexes. The optical properties of the resulting supramolecular assemblies were probed and 2 : 2 assembly in water was found to result in the formation of discrete dimers with tunable luminescence. Two EVs in particular exhibited high absorptivity upon complexation with CB[8] and produce bright blue (EV[Ph]Me) and green (EV[Np]Me) emission, respectively. For these two EVs, the fluorescence spectra exhibited red-shifted, broad, structureless bands with fluorescence quantum yields as high as 0.92 and long fluorescence lifetimes of up to 17 ns. On the other hand, EV[Th]Me with a heteroatom in the bridging moiety, resulted in an emission spectrum typical of excimer-like structures with weak orange luminescence. Here in the benzene-naphthalene–thiophene series, stronger π–π interactions through an increase of aromatic surfaces and, consequently, the restricted rotation or rearrangement in the cavity of CB[8] molecules affect the formation and optical efficiency of excited dimers. Our results suggest that CB[8]-extended aryl viologen supramolecular complexes are a new way to form distinct dimeric entities through a straightforward, synthetically accessible modular approach. These systems represent a platform for studying excited dimers in the excited state such as excimers or exciplexes. Furthermore, systematic modification of a bridging unit of extended aryl viologen derivatives will lead to a range of systems with long-lived excited states. Finally, on account of such interesting optical properties, these distinct dimers can find a myriad of applications in imaging.^6^

## Conflicts of interest

There are no conflicts to declare.

## Supplementary Material

Supplementary information

Supplementary movie

Supplementary movie

Supplementary movie
