# Supplementary material for: Modular supramolecular dimerization of optically tunable extended aryl viologens
Source: Chem Sci. 2019 Aug 12;10(38):8806–11. doi: 10.1039/c9sc03057c (PMC6849629; doi:10.1039/c9sc03057c)
Supplement: Supplementary file 1 [file SC-010-C9SC03057C-s001.pdf]

Electronic Supplementary Material (ESI) for Chemical Science.
This journal is © The Royal Society of Chemistry 2019

Supporting information for: Modular supramolecular dimerization of optically-tunable extended aryl viologens

Magdalena Olesińska,[†] Guanglu Wu,[†] Silvia Gómez-Coca,[‡] Daniel Antón-García,[¶] Istvan Szabo,[‡] Edina Rosta,[‡] and Oren A. Scherman[*,†]

†*Melville Laboratory for Polymer Synthesis, Department of Chemistry, University of Cambridge, Lensfield Road, Cambridge CB2 1EW, UK.*

‡*Department of Chemistry, King's College London, 7 Trinity Street, London, United Kingdom, SE1 1DB, UK.*

¶*Department of Chemistry, University of Cambridge, Lensfield Road, Cambridge CB2 1EW, UK.*

E-mail: oas23@cam.ac.uk

Contents

1 Materials and methods **S3**

2 Synthesis and characterization of extended aryl viologens **S5**

3 ^{1}H NMR and ^{13}C NMR spectra **S10**

4 High resolution mass spectrometry (HRMS) **S25**

5 ^{1}H NMR titration of extended viologens into CB[8] and CB[7] **S35**

6 Isothermal titration thermograms **S45**

7 Steady-state and time-resolved photophysical characterisation of extended viologens before and after adding CB[8] and CB[7] **S53**

8 Computational details **S57**

8.1 Molecular dynamics S57

8.2 TB-DFT calculations S58

Starting geometries S58

Geometry optimization S58

Spectroscopic data S63

References **S65**

1 Materials and methods

Materials All the materials as following were purchased from commercial suppliers and used without further purification: 4-Pyridinylboronic acid (97 %, Fisher Scientific), 1,4-Dibromonaphthalene (>98 %, Acros Organics), 1,4-Dibromobenzene (>98 %, Alfa Aesar), 2,5-Dibromothiophene (96 %, Alfa Aesar), Tetrakis(triphenylphosphine) palladium(0) (99.8 % (metals basis), Pd 9 % min, Alfa Aesar), aniline (>99.5 %, Sigma-Aldrich), p-Toluidine (>99 %, Alfa Aesar), p-Anisidine (>99 %, Sigma-Aldrich), 4-(Methylthio)aniline (>98 %, Alfa Aesar), p-Phenylenediamine (>97 %, Alfa Aesar), N,N-Dimethyl-p-phenylenedia9mine (>97 %, Sigma-Aldrich), 4-(Trifluoromethyl)aniline (99 %, Sigma-Aldrich), 4-Bromoaniline (97 %, Sigma-Aldrich), 1-adamantanamine hydrochloride (>97 %, Sigma-Aldrich), Potassium Hydrogen Carbonate (anhydrous, Fisher Scientific), N,N-Dimethylformamide (anhydrous, Sigma-Aldrich), toluene (Fisher Scientific), ethanol (absolute, Fisher Scientific), and diethyl ether (>99 %, Aldrich). Milli-Q water (18.2 MΩ cm) was used for preparation of all non-deuterated aqueous solutions, Cucurbit[8]uril (CB[8]) was synthesized according to the published procedure.[S1]

Isothermal Titration Calorimetry (ITC) All ITC experiments were carried out on a Microcal ITC200 at 298.15 K in 50 mM sodium acetate buffer (pH = 4.75). In a typical ITC, the host molecule (CB[8]) was in the sample cell, and guest molecule was in the injection syringe with a concentration of about ten times concentration of host. The concentration of CB[8] was calibrated by the titration with a standard solution of 1-adamantanamine. All raw data of ITC were integrated by NITPIC (v.1.2.0), fitted in Sedphat (v.12.1b), and visualized through GUSSI (v.1.1.0).

Nuclear Magnetic Resonance Spectroscopy (NMR) ^{1}H NMR spectra were acquired in heavy water (D_2O) at 298 K, and recorded on a Bruker AVANCE 500 with TCI Cryoprobe Spectrometer (500 MHz) being controlled by TopSpin2. ^{13}C NMR (126 MHz) spectra were

acquired in heavy water (D_2O), and recorded on a Bruker AVANCE III QNP Cryoprobe with external reference TMS set to 0 ppm. Titrations were performed by using stock solutions of host and guest calibrated against the concentrated solution of 3-(Trimethylsilyl)-1-propane-sulfonic acid sodium salt.

HPLC-LCMS HPLC-LCMS was performed on Waters ZQ with Waters 2795, with 50 x 4.6 mm C18 column (Phenomenex Kinetix solid core column), using acetonitrile:water (1:1) as a mobile phase.

UV/Vis Spectroscopy UV/Vis spectra were recorded on a Varian Cary 400 UV/Vis spectrophotometer using a Suprasil Quartz (QS) cuvette with 1 cm path length at 298 K. EV[X]R, EV[X]R-CB[8] and EV[X]R-CB[7] aqueous solutions were tested in the concentration of 0.01 mM.

Fluorescence Spectroscopy Fluorescence spectroscopy was recorded on an Agilent Varian Cary Eclipse fluorescence spectrophotometer using a Suprasil Quartz (QS) cuvette with 1 cm path length at 298 K.

Time-Correlated Single Photon Counting TCSPC was recorded using a FS5 Spectrofluorometer, Edinburgh Instrument. A TCSPC laser (360 nm) was used for fluorescence lifetime measurements at peak emission wavelength for measured molecules and complexes. A Suprasil Quartz (QS) cuvette with 1 cm path length was used for all measurements. The data was fit with the exponential reconvolution function and the non-linear least square method.

Fluorescence Quantum Yield Measurements The absolute fluorescence quantum yield was determined by using an Edinburgh Instruments Integrating Sphere Module (SC-30) on the FS5 spectrofluorometer. Fluorescence spectra analysis software Fluoracle and Quantum

Yield Wizard were used to obtain quantum yields. The 1.5 ml Suprasil Quartz (QS) cuvette with 1 cm path length was used for all measurements at 298 K. Fluorescence quantum yield measurements were done for 1 ml aqueous solutions of EV[X]R, EV[X]R-CB[8] and EV[X]R-CB[7] with an optical density in the range of 0.1-0.06.

2 Synthesis and characterization of extended aryl viologens

Synthesis of EV[Ph], EV[Np] and EV[Th] were adapted and modified from previously reported procedures.[S2,S3]

1,4-di(pyridin-4-yl)benzene - EV[Ph]

4-Pyridinylboronic acid (1.25 g, 10 mmol), 1,4-dibromobenzene (1 g, 4.2 mmol) and K_2CO_3 (2.8 g, 20.4 mmol) were added to a 1:1 mixture of dry, degassed toluene and DMF (300 ml). Next, $Pd(PPh_3)_4$ (0.39 g, 0.34 mmol) was added to the reaction mixture and the solution heated to 130 °C under N_2 for 72 h. Thereafter, the reaction mixture was cooled to room temperature and filtered. The organic phase was concentrated under vacuum and the residue dissolved in CH_3Cl and washed three times with water. The collected organic phase was concentrated under vacuum and the residue dissolved in acetone. Concentrated HCl was then added dropwise to the solution, resulting in precipitation of the product. The precipitate was collected by filtration and then dissolved in H_2O. Finally, aqueous NaOH (10 M) was added dropwise to the H_2O layer until the pH was ca. 8-9, resulting in the precipitation of pure white powder of EV[Ph] (0.8 g, 82 %). **^{1}H NMR** (500 MHz, $CDCl_3$) δ (ppm): 8.70 (dd, J = 4.75; 1.51 Hz, 4H), 7.77 (s, 4H), 7.56 (dd, J = 4.55; 1.61 Hz, 4H). **^{13}C NMR** (126 MHz, $CDCl_3$) δ (ppm): 150.30, 147.52, 138.82, 127.79, 121.57 **MS** ESI-MS: m/z [M+H]+ calc for $C_{16}H_{12}N_2H$: 233.1073, found: 233.1074

EV[Np] and EV[Th] were obtained using the same general method with appropriate 1,4-dibromonapthyl and 2,5-Dibromothiophene, respectively.

1,4-di(pyridin-4-yl)naphthalene - EV[Np]

^{1}H NMR (500 MHz, $CDCl_3$) δ (ppm): 8.77 (dd, J = 4.45; 1.53 Hz, 4H), 7.90 (m, 2H), 7.51 (m, J = 4.55; 8H). **^{13}C NMR** (126 MHz, $CDCl_3$ δ (ppm): 149.38, 148.96, 138.09, 131.10, 127.03, 126.36, 125.80, 125.17. **MS** ESI-MS: m/z [M+H]+ calc for $C_{20}H_{14}N_2H$: 283.1230, found: 283.1229

4,4'-(thiophene-2,5-diyl)bis(1-methylpyridin-1-ium) - EV[Th]

^{1}H NMR (500 MHz, $CDCl_3$) δ (ppm): 8.64 (d, J = 6.3 Hz, 4H), 7.55 (s, 2H), 7.53 – 7.50 (m, 4H). **^{13}C NMR** (126 MHz, $CDCl_3$) δ (ppm): 150.38, 142.61, 140.85, 126.64, 119.79. **MS** ESI-MS: m/z [M+H]+ calc for $C_{14}H_{10}N_2SH$: 239.0637, found: 239.0637

The symmetric Zinke salt for all EV[X]DNB was prepared using general procedure described below.

4,4'-(1,4-phenylene)bis(1-(2,4-dinitrophenyl)pyridin-1-ium) - EV[Ph]DNB

1,4-di(pyridin-4-yl)benzene (1 g, 4.3 mmol) and 1-chloro-2,4-dinitrobenzene (12.91 g, 2.6 mmol) were refluxed for 72 h in ethanol. After that, the reaction mixture was cooled down to room temperature and concentrated. The residue was precipitated with diethyl ether and dried. The precipitate was purify via reverse-phase HPLC with eluent gradient of H_2O/MeCN/0.1 % TFA. Collected fractions were concentrated in vacuum, dissolved in 2 mM aqueous HCl and then lyophilized. Pure yellow solid in 65 % yield was obtained. **^{1}H NMR** (500 MHz, D_2O) δ (ppm): 9.33 (d, J = 2.5 Hz, 2H), 9.15 – 9.08 (m, 4H), 8.87 (dd, J = 8.8, 2.5 Hz, 2H), 8.67 – 8.60 (m, 4H), 8.29 (s, 4H), 8.21 (d, J = 8.7 Hz, 2H). **^{13}C NMR** (126 MHz, D_2O) δ (ppm): 158.28, 149.58, 145.28, 143.03, 138.50, 137.28, 131.17, 130.56, 129.76, 125.58, 122.69 **MS** ESI-MS: m/z [M]2+ calc for $C_{28}H_{18}N_6O_8$: 283.0588, found: 283.0581

4,4'-(naphthalene-1,4-diyl)bis(1-(2,4-dinitrophenyl)pyridin-1-ium) - EV[Np]DNB
^{1}H NMR (500 MHz, D_2O) δ (ppm): 9.34 (d, J = 2.5 Hz, 2H), 9.20 (dd, J = 5.0, 1.8 Hz, 4H), 8.88 (dd, J = 8.6, 2.5 Hz, 2H), 8.53 (dd, J = 5.1, 1.9 Hz, 4H), 8.25 (d, J = 8.7 Hz, 2H), 8.06 (dd, J = 6.5, 3.4 Hz, 2H), 7.92 (s, 2H), 7.74 (dd, J = 6.5, 3.2 Hz, 2H). **^{13}C NMR** (126 MHz, D_2O) δ (ppm): 160.28, 149.65, 145.16, 143.04, 138.58, 136.61, 131.24, 130.62, 130.07, 129.25, 128.84, 128.14, 125.12, 122.75. **MS** ESI-MS: m/z [M]2+ calc for $C_{32}H_{20}N_6O_8$: 308.0666, found: 308.0722

4,4'-(thiophene-2,5-diyl)bis(1-(2,4-dinitrophenyl)pyridin-1-ium) - EV[Th]DNB
^{1}H NMR (500 MHz, D_2O) δ (ppm): 9.32 (s, 2H), 9.02 (d, J = 6.1 Hz, 4H), 8.86 (d, J = 11.2 Hz, 2H), 8.54 (d, J = 4.8 Hz, 4H), 8.33 – 8.27 (m, 2H), 8.19 (d, J = 9.7 Hz, 2H) **^{13}C NMR** (126 MHz, D_2O) δ (ppm): 150.75, 149.54, 145.26, 143.85, 143.04, 138.46, 134.61, 131.16, 130.57, 123.54, 122.70, 117.47, 115.14. **MS** ESI-MS: m/z [M]2+ calc for $C_{26}H_{16}N_6O_8S$: 286.0370, found: 286.0372

The procedure below was used to synthesized the substituted EV[X]Rs (where X= [Ph], [Np], [Th] and depending on molecule R=H, Me, OMe, SMe, NH_2, NMe_2, CF_3, Br) starting from appropriate symmetric Zinke salt and amine.

4,4'-(1,4-phenylene)bis(1-(p-tolyl)pyridin-1-ium) dichloride - EV[Ph]Me
4,4'-(1,4-phenylene)bis(1-(2,4-dinitrophenyl)pyridin-1-ium) (1 g, 1.6 mmol) and toluidine (0.5 g, 4.7 mmol) were refluxed for 24 h in 100 ml of ethanol. After that, the reaction mixture was cooled down to room temperature and concentrated. The residue was precipitated with diethyl ether and dried. **^{1}H NMR** (500 MHz, D_2O) δ (ppm): 9.03 (d, J = 6.8 Hz, 4H), 8.45 (d, J = 6.9 Hz, 4H), 8.16 (s, 4H), 7.58 (d, J = 8.5 Hz, 4H), 7.48 (d, J = 8.4 Hz, 4H), 2.39 (s, 6H). **^{13}C NMR** (126 MHz, D_2O) δ (ppm): 155.55, 144.09, 142.58, 139.87, 137.03, 130.92, 129.32, 125.32, 123.55, 20.26. **MS** ESI-MS: m/z [M]2+ calc for $C_{30}H_{26}N_2$: 207.1043, found: 207.1041

4,4'-(1,4-phenylene)bis(1-phenylpyridin-1-ium) dichloride - EV[Ph]H

^{1}H NMR (500 MHz, D_2O) δ (ppm): 9.07 (dd, J = 5.3, 1.5 Hz, 4H), 8.49 (dd, J = 5.2, 1.2 Hz, 4H), 8.19 (s, 4H), 7.74 – 7.63 (m, 10H). **^{13}C NMR** (126 MHz, D_2O) δ (ppm): 162.84, 155.93, 144.28, 142.27, 137.09, 131.52, 130.52, 129.37, 125.39, 123.92. **MS** ESI-MS: m/z [M]2+ calc for $C_{28}H_{22}N_2$: 193.0886, found: 193.0882

4,4'-(1,4-phenylene)bis(1-(4-methoxyphenyl)pyridin-1-ium) dichloride - EV[Ph]OMe

^{1}H NMR (500 MHz, D_2O) δ (ppm): 8.97 (d, J = 7.0 Hz, 4H), 8.39 (d, J = 7.0 Hz, 4H), 8.12 (s, 4H), 7.65 – 7.59 (m, 4H), 7.19 – 7.14 (m, 4H), 3.82 (s, 6H). **^{13}C NMR** (126 MHz, D_2O) δ (ppm): 161.23, 155.02, 143.91, 136.88, 135.40, 129.28, 125.24, 125.23, 115.57, 55.82. **MS** ESI-MS: m/z [M]2+ calc for $C_{30}H_{26}N_2O_2$: 223.0992, found: 223.0986

4,4'-(naphthalene-1,4-diyl)bis(1-(p-tolyl)pyridin-1-ium) dichloride - EV[Np]Me

^{1}H NMR (500 MHz, D_2O) δ (ppm): 9.13 (dd, J = 5.0, 1.9 Hz, 4H), 8.37 (dd, J = 5.3, 1.6 Hz, 4H), 7.99 (dd, J = 6.5, 3.3 Hz, 2H), 7.82 (s, 2H), 7.70 (dd, J = 6.5, 3.3 Hz, 2H), 7.66 – 7.60 (m, 4H), 7.51 (d, J = 7.7 Hz, 4H), 2.42 (s, 6H). **^{13}C NMR** (126 MHz, D_2O) δ (ppm): 157.50, 143.95, 142.64, 140.06, 136.45, 130.95, 130.12, 129.03, 128.50, 127.69, 125.11, 123.69, 20.26. **MS** ESI-MS: m/z [M]2+ calc for $C_{34}H_{28}N_2$: 232.1121, found: 232.1118

4,4'-(naphthalene-1,4-diyl)bis(1-phenylpyridin-1-ium) dichloride - EV[Np]H

^{1}H NMR (500 MHz, D_2O) δ (ppm): 9.18 (d, J = 6.7 Hz, 4H), 8.41 (d, J = 6.6 Hz, 4H), 8.01 (dd, J = 6.5, 3.3 Hz, 2H), 7.84 (s, 2H), 7.77 (dd, J = 7.0, 2.9 Hz, 4H), 7.74 – 7.67 (m, 8H). **MS** ESI-MS: m/z [M]2+ calc for $C_{32}H_{24}N_2$: 218.0964, found: 218.0965

4,4'-(naphthalene-1,4)bis(1-(4-methoxyphenyl)pyridin-1-ium) dichloride - EV[Np]OMe

^{1}H NMR (500 MHz, D_2O) δ (ppm): 9.10 (dd, J = 5.3, 1.5 Hz, 4H), 8.35 (dd, J = 5.2, 1.3 Hz, 4H), 7.99 (dd, J = 6.5, 3.3 Hz, 2H), 7.81 (s, 2H), 7.73 – 7.68 (m, 6H), 7.22 (dd, J = 6.8, 2.4 Hz, 4H), 3.87 (s, 6H). **^{13}C NMR** (126 MHz, D_2O) δ (ppm): 161.27, 157.26, 143.96,

136.47, 135.76, 130.15, 129.03, 128.51, 127.70, 125.48, 125.13, 115.62, 55.87. **MS** ESI-MS: m/z [M]2+ calc for $C_{34}H_{28}N_2O_2$: 248.1070, found: 248.1070

4,4'-(naphthalene-1,4-diyl)bis(1-(4-(methylthio)phenyl)pyridin-1-ium) dichloride - EV[Np]SMe

^{1}H NMR (500 MHz, D_2O) δ (ppm): 9.17 – 9.15 (m, 2H), 8.45 – 8.36 (m, 2H), 8.01 (dd, J = 6.5, 3.3 Hz, 1H), 7.84 (s, 1H), 7.75 – 7.67 (m, 3H), 7.61 – 7.53 (m, 2H), 2.55 (s, 3H). **^{13}C NMR** (126 MHz, D_2O) δ (ppm): 143.86, 143.45, 136.48, 130.14, 129.11, 128.54, 127.75, 127.02, 126.45, 125.74, 125.14, 124.32, 14.12. **MS** ESI-MS: m/z [M]2+ calc for $C_{34}H_{28}N_2S_2$: 264.0841, found: 264.0838

4,4'-(naphthalene-1,4-diyl)bis(1-(4-aminophenyl)pyridin-1-ium) dichloride - EV[Np]NH$_2$

^{1}H NMR (500 MHz, D_2O) δ (ppm): 9.13 – 9.02 (m, 4H), 8.40 – 8.29 (m, 4H), 8.00 (dd, J = 6.5, 3.3 Hz, 2H), 7.82 (s, 2H), 7.70 (dd, J = 6.5, 3.3 Hz, 2H), 7.61 – 7.47 (m, 4H), 7.07 – 6.95 (m, 4H), 6.66 (s, 1H). **MS** ESI-MS: m/z [M]2+ calc for $C_{32}H_{26}N_4$: 233.1073, found: 233.1075

4,4'-(naphthalene-1,4-diyl)bis(1-(4-(dimethylamino)phenyl)pyridin-1-ium) dichloride - EV[Np]NMe$_2$

^{1}H NMR (500 MHz, D_2O) δ (ppm): 9.10 – 9.04 (m, 2H), 8.35 – 8.29 (m, 2H), 8.01 (dd, J = 6.5, 3.3 Hz, 1H), 7.82 (s, 1H), 7.71 (dd, J = 6.6, 3.2 Hz, 1H), 7.66 – 7.61 (m, 2H), 7.07 – 7.02 (m, 2H), 2.99 (s, 6H). **MS** ESI-MS: m/z [M]2+ calc for $C_{36}H_{34}N_4$: 261.1386, found: 261.1390

4,4'-(naphthalene-1,4-diyl)bis(1-(4-(trifluoromethyl)phenyl)pyridin-1-ium) dichloride - EV[Np]CF$_3$

^{1}H NMR (500 MHz, D_2O) δ (ppm): 9.32 (d, J = 6.9 Hz, 2H), 8.59 – 8.51 (m, 2H), 8.18 – 8.03 (m, 5H), 7.95 (s, 1H), 7.81 (dt, J = 6.6, 3.3 Hz, 1H). **MS** ESI-MS: m/z [M]2+ calc for

$C_{34}H_{22}F_6N_2$: 286.0838 , found: 286.0840

4,4'-(naphthalene-1,4-diyl)bis(1-(4-bromophenyl)pyridin-1-ium) dichloride - EV[Np]Br
^{1}H NMR (500 MHz, D_2O) δ (ppm): 9.22 – 9.15 (m, 2H), 8.46 – 8.40 (m, 2H), 8.01 (dd, J = 6.5, 3.3 Hz, 1H), 7.93 – 7.88 (m, 2H), 7.85 (s, 1H), 7.74 – 7.68 (m, 3H) **MS** ESI-MS: m/z [M]2+ calc for $C_{32}H_{22}Br_2N_2$: 297.0059, found: 297.0062

4,4'-(thiophene-2,5-diyl)bis(1-(p-tolyl)pyridin-1-ium) dichloride - EV[Th]Me
^{1}H NMR (500 MHz, D_2O) δ (ppm): 9.03 – 8.85 (m, 2H), 8.42 – 8.33 (m, 2H), 8.15 (s, 1H), 7.60 – 7.52 (m, 2H), 7.46 (d, J = 8.2 Hz, 2H), 2.38 (s, 3H). **^{13}C NMR** (126 MHz, D_2O) δ (ppm): 148.36, 144.11, 143.09, 142.55, 139.87, 133.39, 130.91, 123.46, 123.39, 20.26. **MS** ESI-MS: m/z [M]2+ calc for $C_{28}H_{24}N_2S$: 210.0825, found: 210.0828

3 ^{1}H NMR and ^{13}C NMR spectra

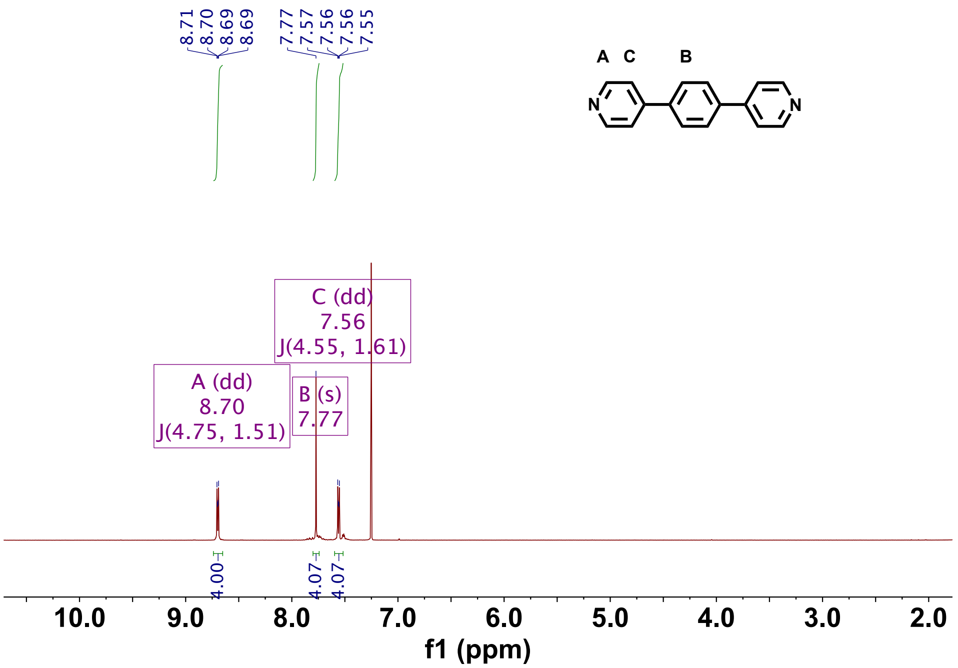

Figure S1: ^{1}H NMR of EV[Ph] (500 MHz, 298.15 K, $CDCl_3$)

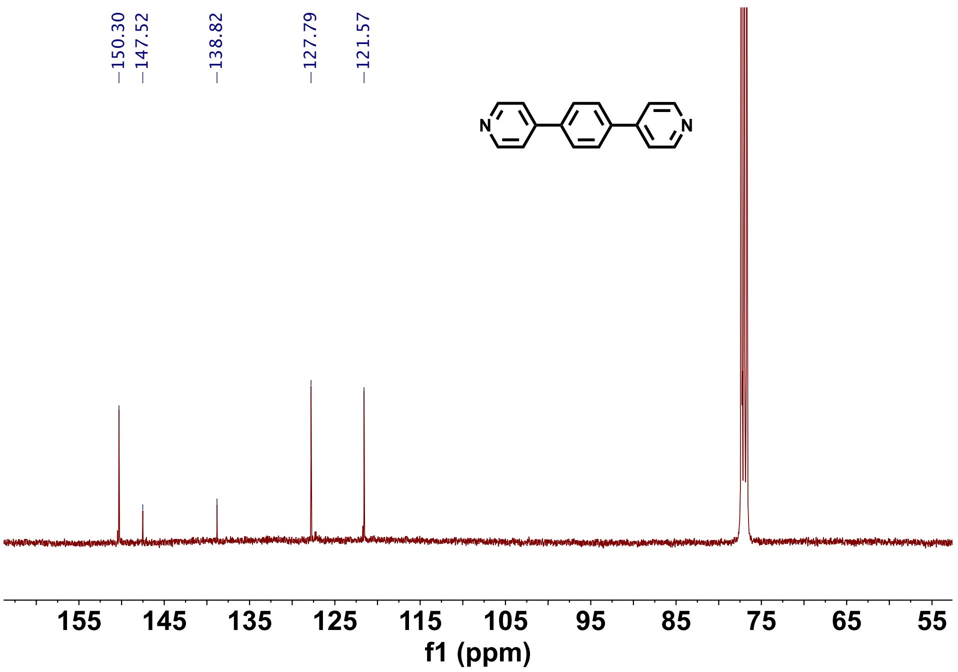

Figure S2: ^{13}C NMR of EV[Ph] (126 MHz, 298.15 K, $CDCl_3$)

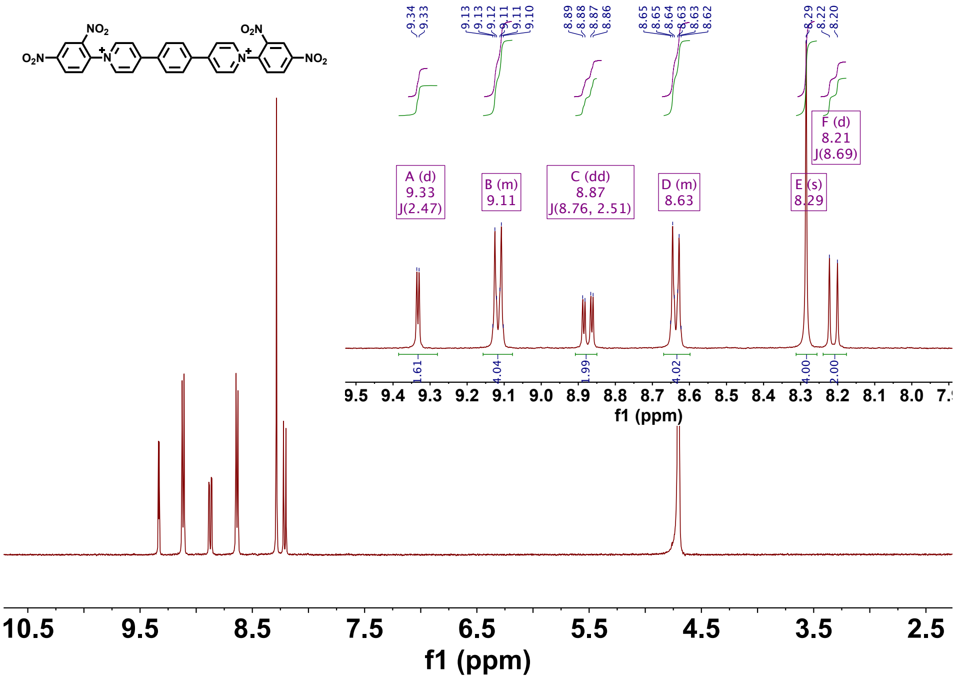

Figure S3: ^{1}H NMR of EV[Ph]DNB (500 MHz, 298.15 K, D_2O)

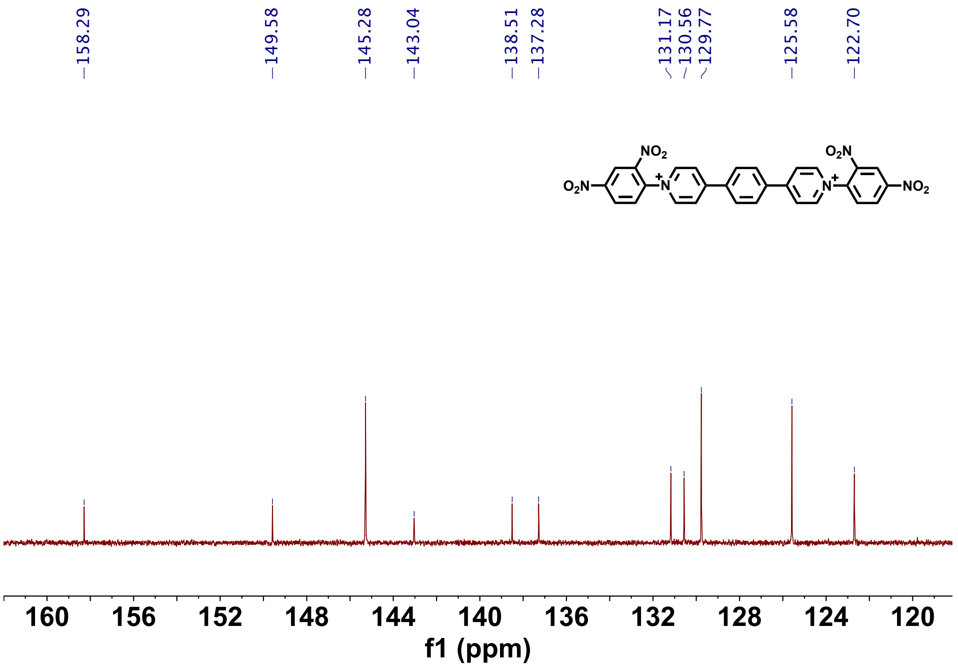

Figure S4: ^{13}C NMR of EV[Ph]DNB (126 MHz, 298.15 K, D_2O)

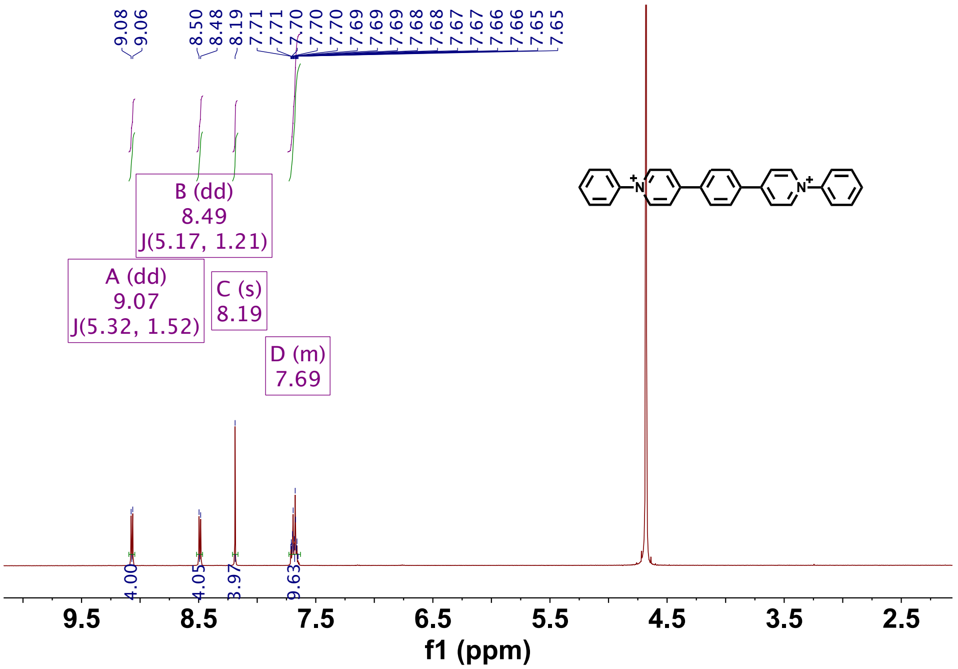

Figure S5: ^{1}H NMR of EV[Ph]H (500 MHz, 298.15 K, D_2O)

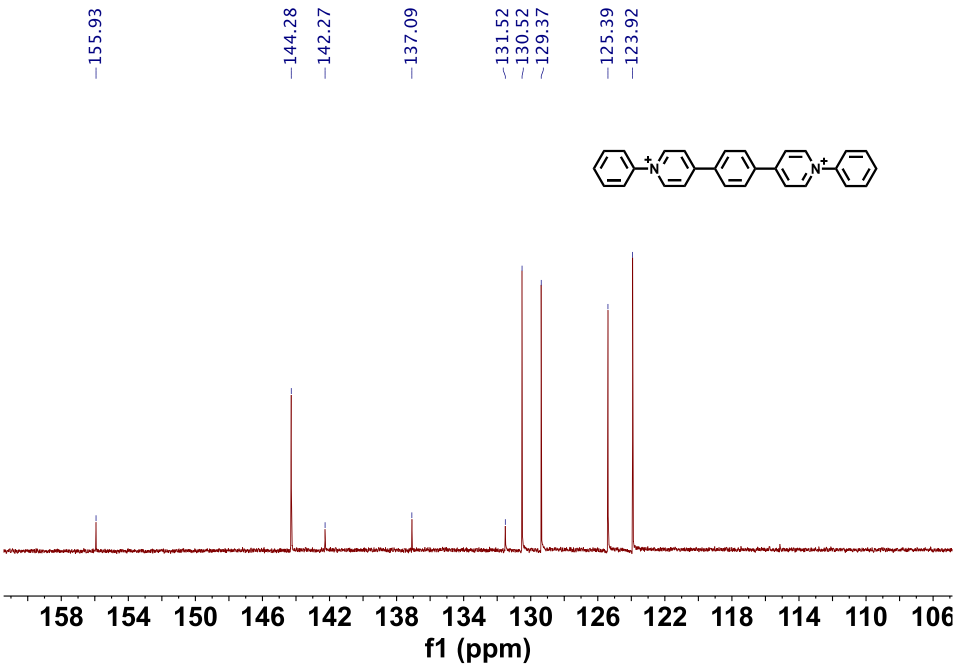

Figure S6: ^{13}C NMR of EV[Ph]H (126 MHz, 298.15 K, D_2O)

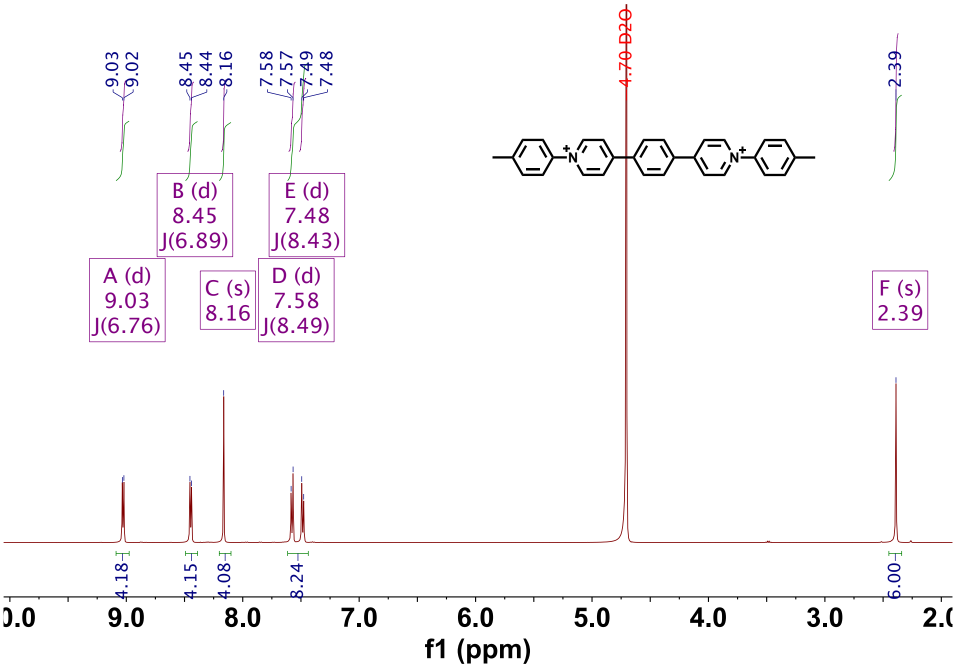

Figure S7: ^{1}H NMR of EV[Ph]Me (500 MHz, 298.15 K, D_2O)

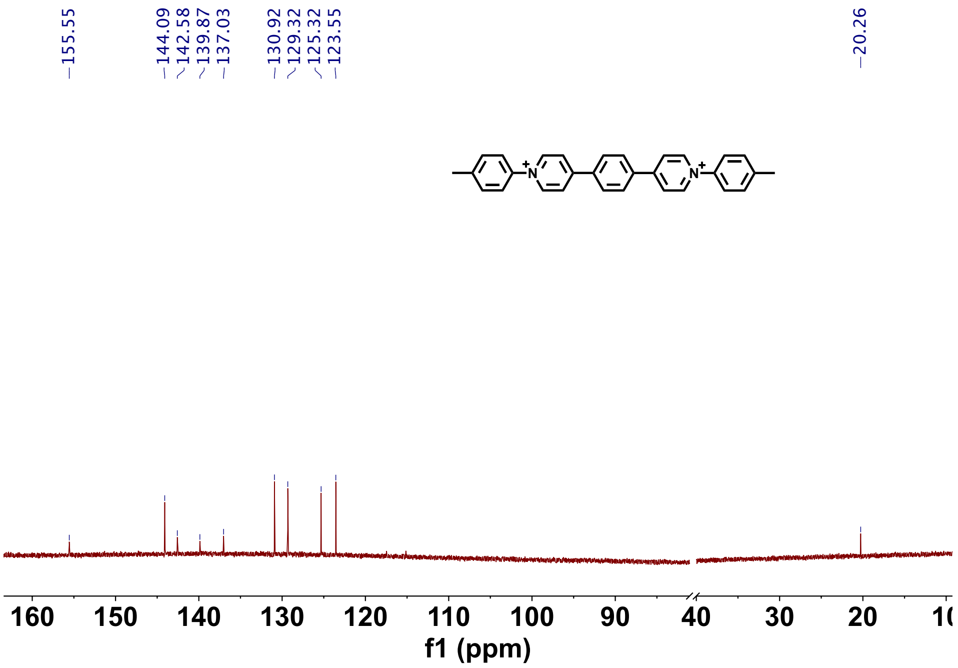

Figure S8: ^{13}C NMR of EV[Ph]Me (126 MHz, 298.15 K, D_2O)

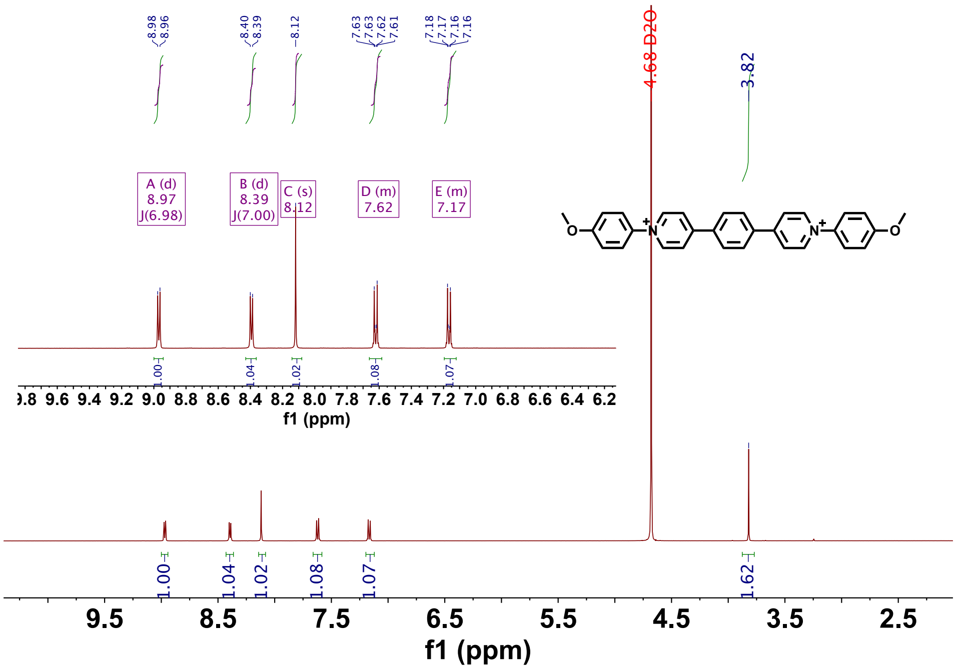

Figure S9: ^{1}H NMR of EV[Ph]OMe (500 MHz, 298.15 K, D_2O)

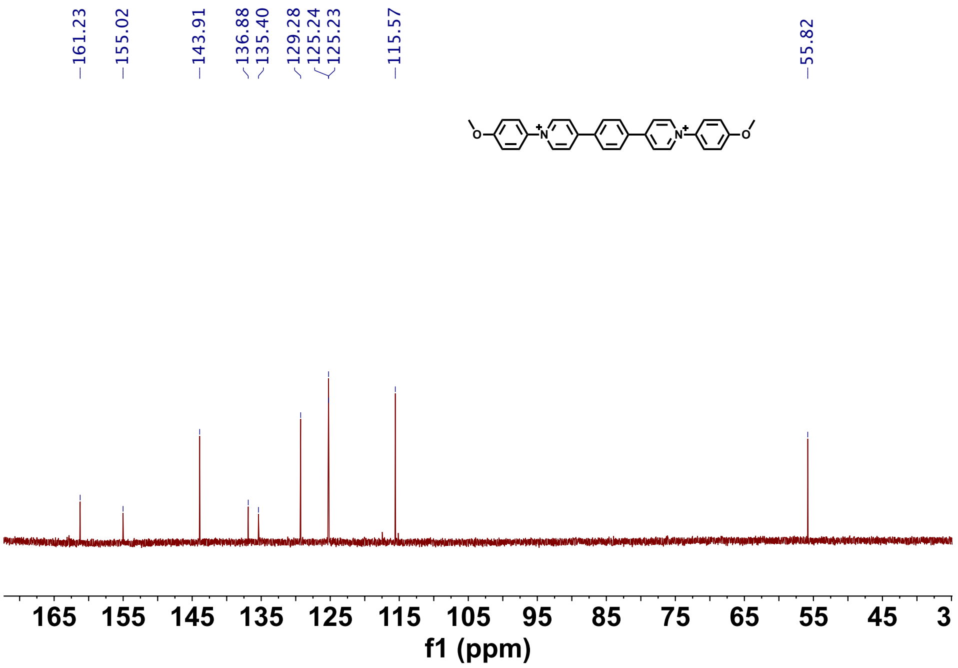

Figure S10: ^{13}C NMR of EV[Ph]OMe (126 MHz, 298.15 K, D_2O)

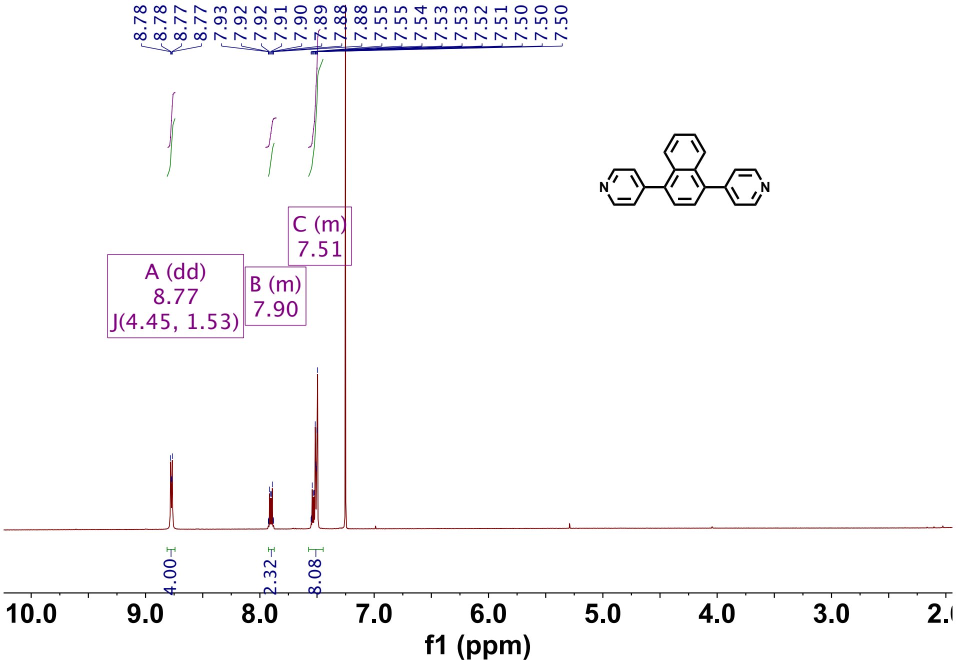

Figure S11: ^{1}H NMR of EV[Np] (500 MHz, 298.15 K, $CDCl_3$)

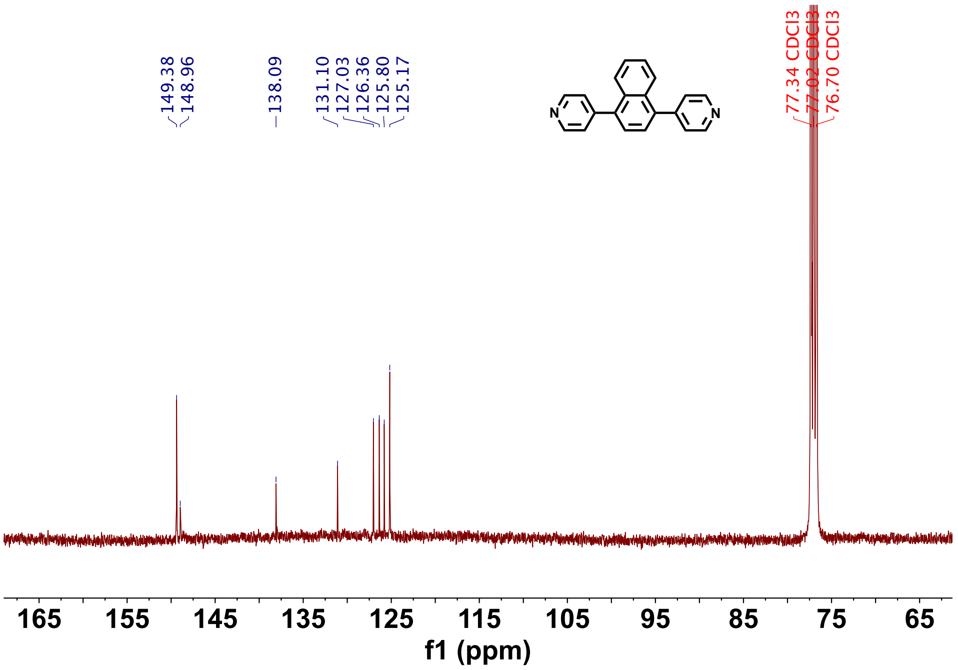

Figure S12: ^{13}C NMR of EV[Np] (126 MHz, 298.15 K, $CDCl_3$)

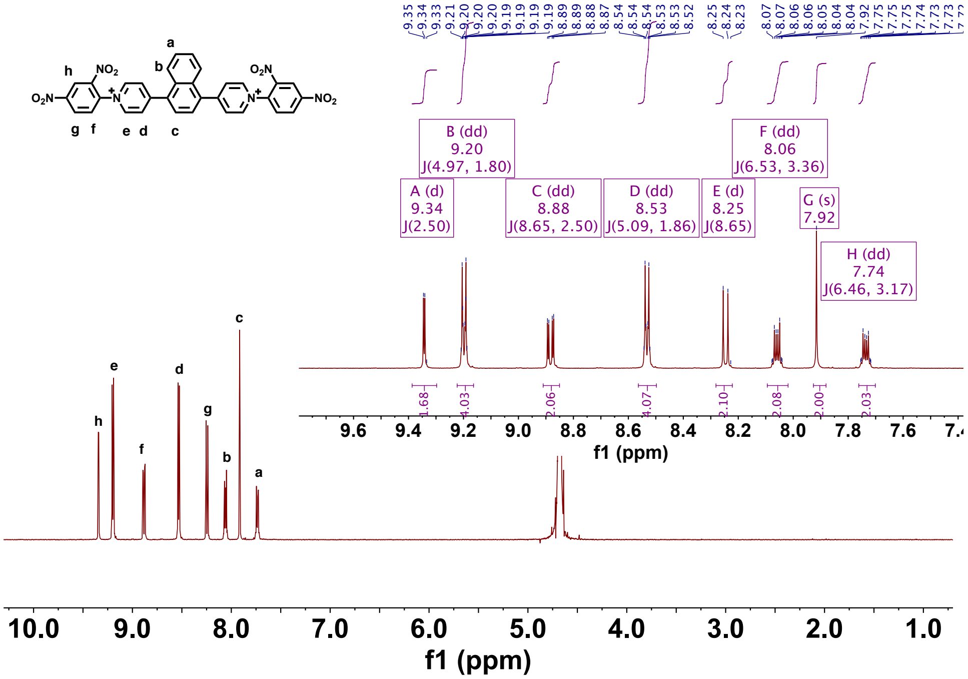

Figure S13: ^{1}H NMR of EV[NP]DNB (500 MHz, 298.15 K, D_2O)

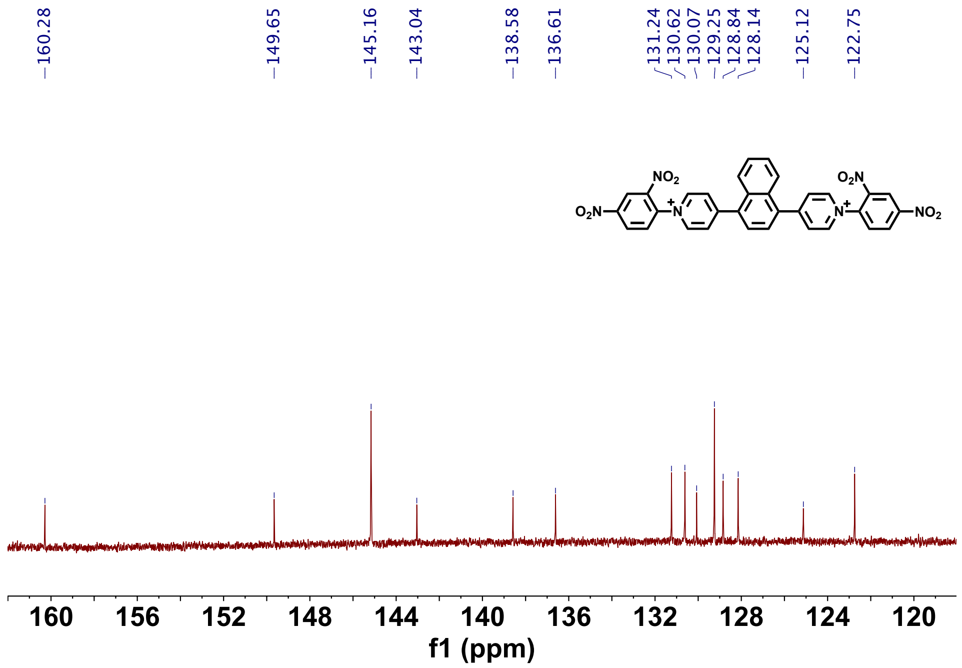

Figure S14: ^{13}C NMR of EV[Np]DNB (126 MHz, 298.15 K, D_2O)

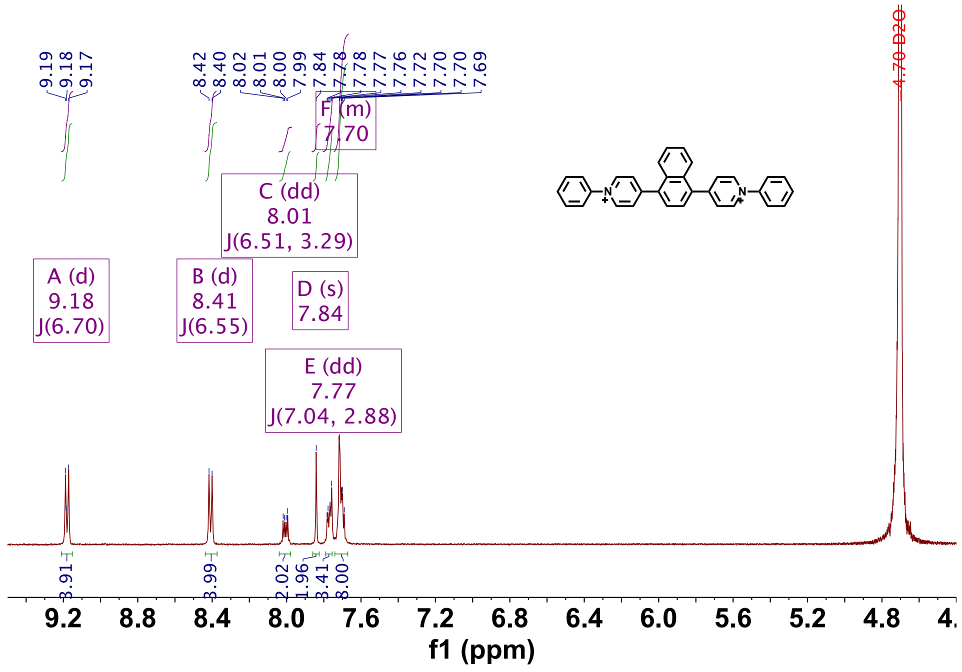

Figure S15: ^{1}H NMR of EV[Np]H (500 MHz, 298.15 K, D_2O)

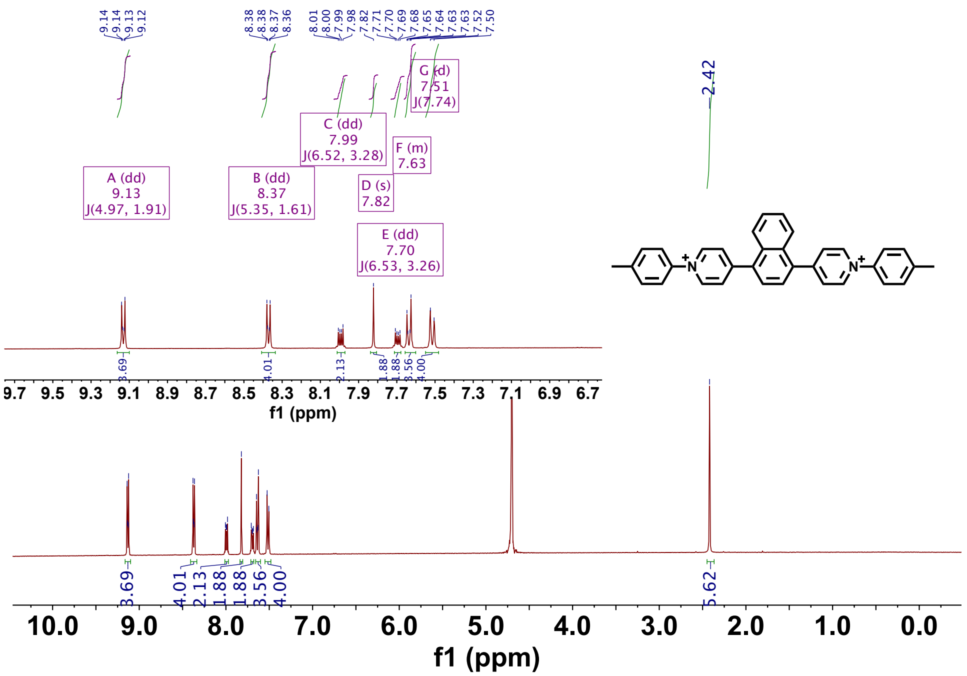

Figure S16: ^{1}H NMR of EV[Np]Me (500 MHz, 298.15 K, D_2O)

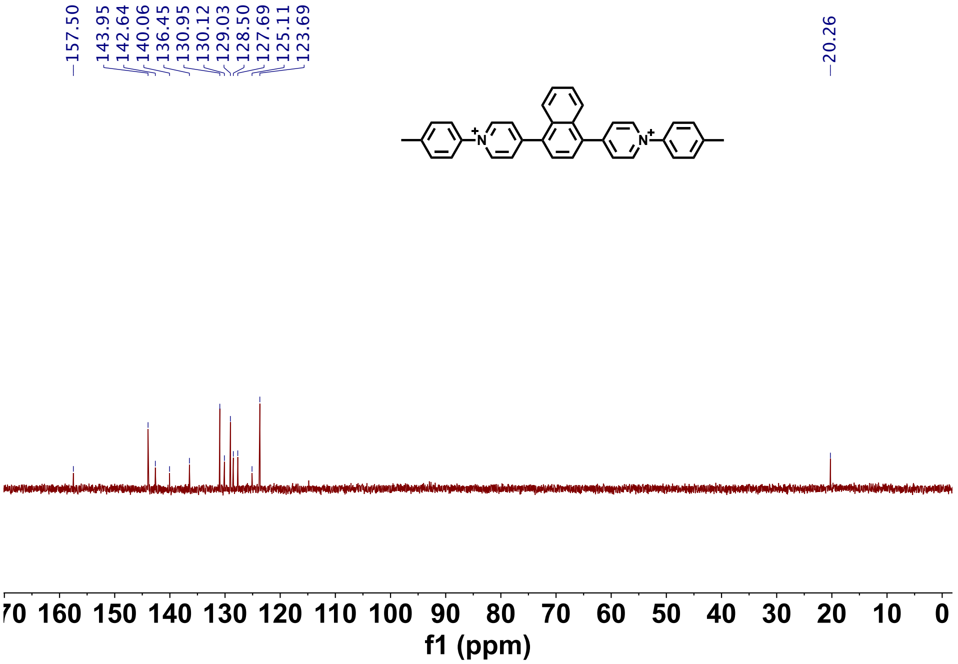

Figure S17: ^{13}C NMR of EV[Np]Me (126 MHz, 298.15 K, D_2O)

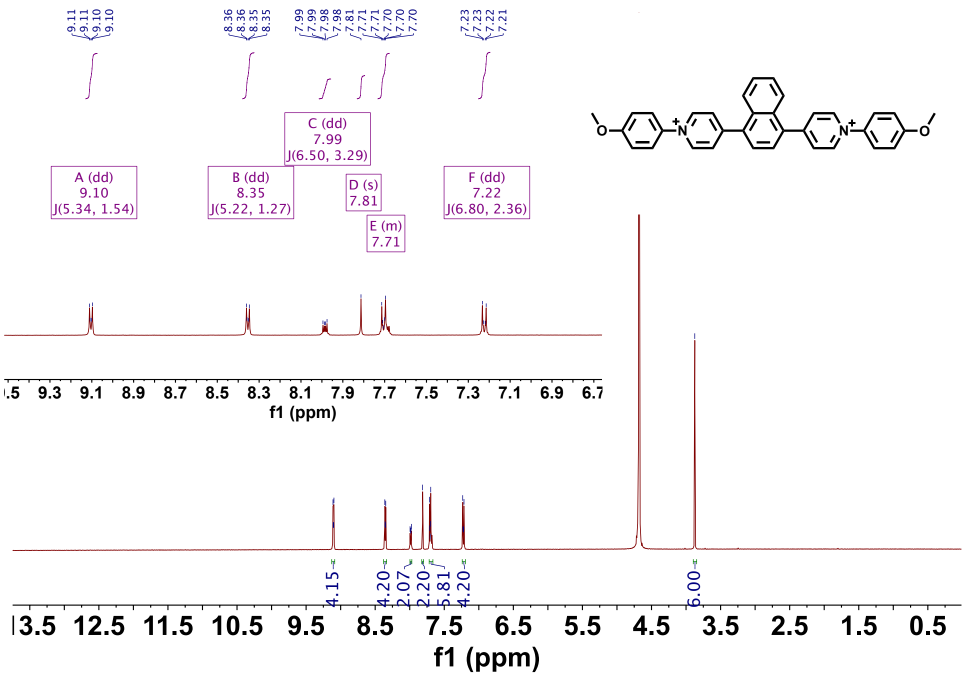

Figure S18: ^{1}H NMR of EV[Np]OMe (500 MHz, 298.15 K, D_2O)

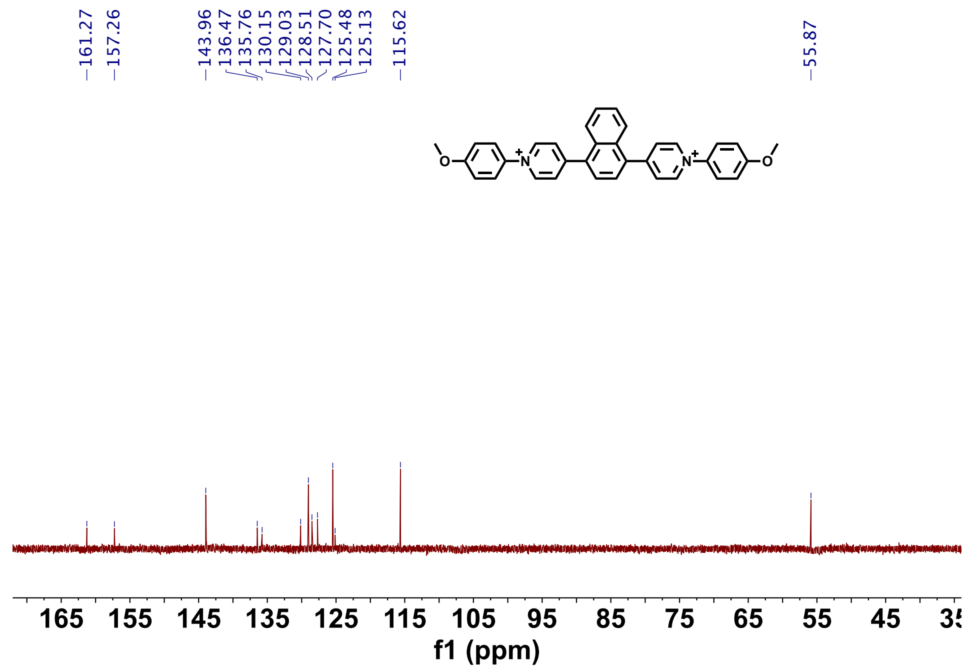

Figure S19: ^{13}C NMR of EV[Np]OMe (126 MHz, 298.15 K, D_2O)

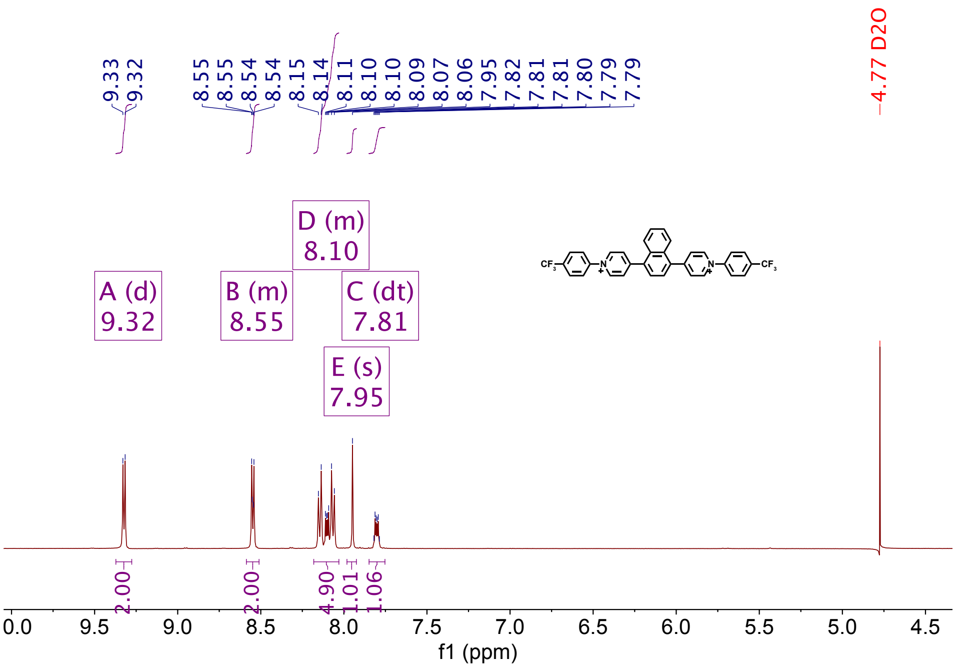

Figure S20: ^{1}H NMR of EV[Np]CF_3 (500 MHz, 298.15 K, D_2O)

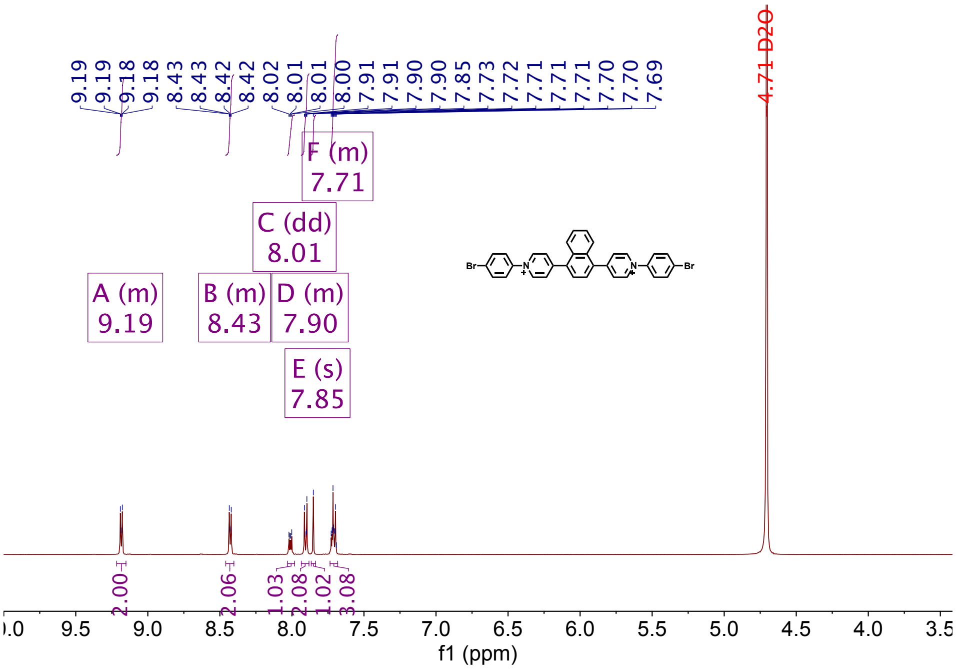

Figure S21: ^{1}H NMR of EV[Np]Br (500 MHz, 298.15 K, D_2O)

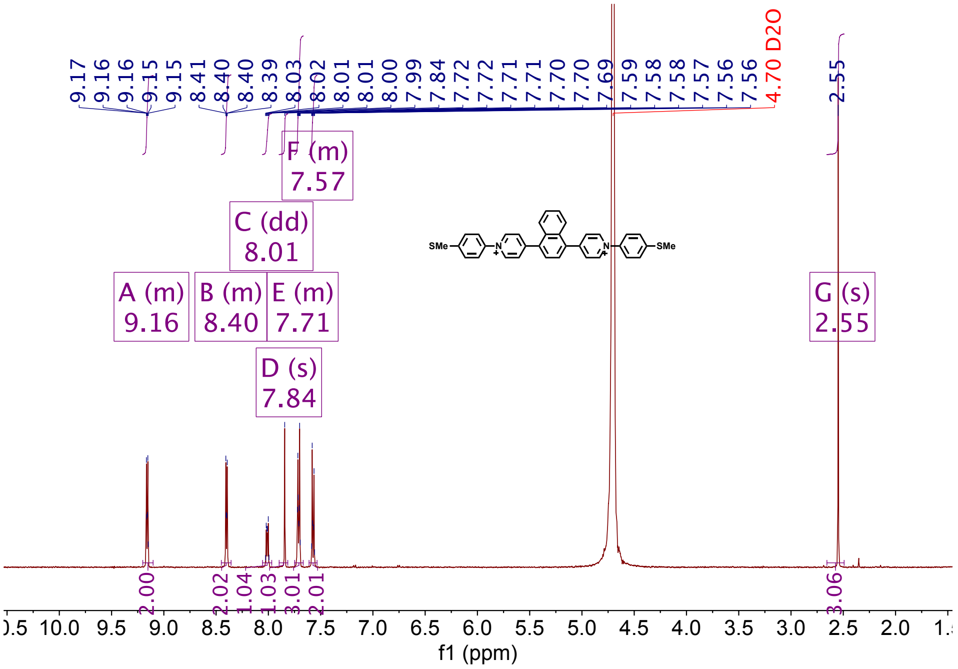

Figure S22: ^{1}H NMR of EV[Np]SMe (500 MHz, 298.15 K, D_2O)

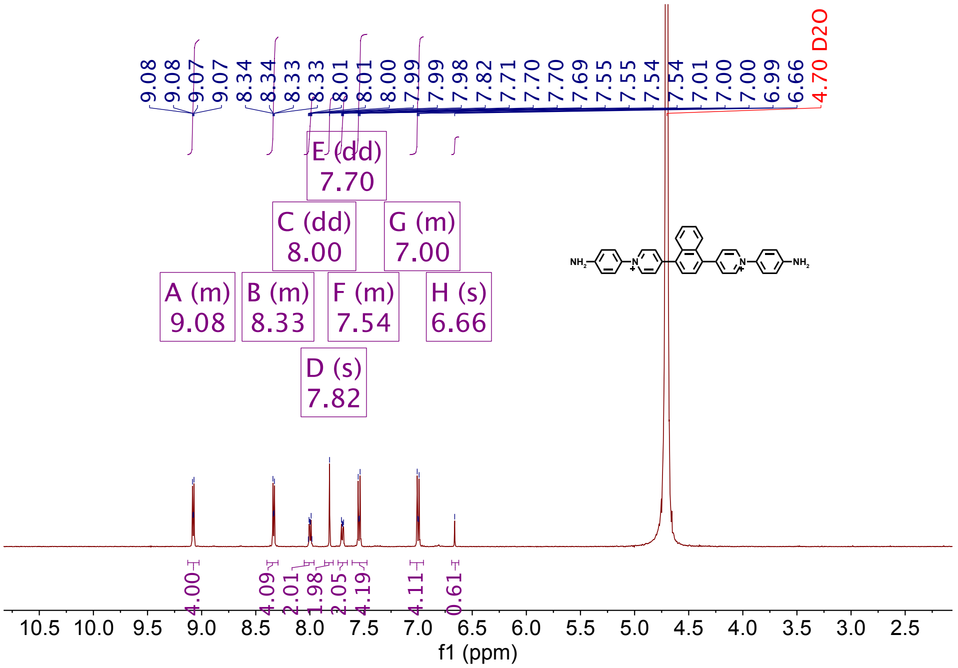

Figure S23: ^{1}H NMR of EV[Np]NH_2 (500 MHz, 298.15 K, D_2O)

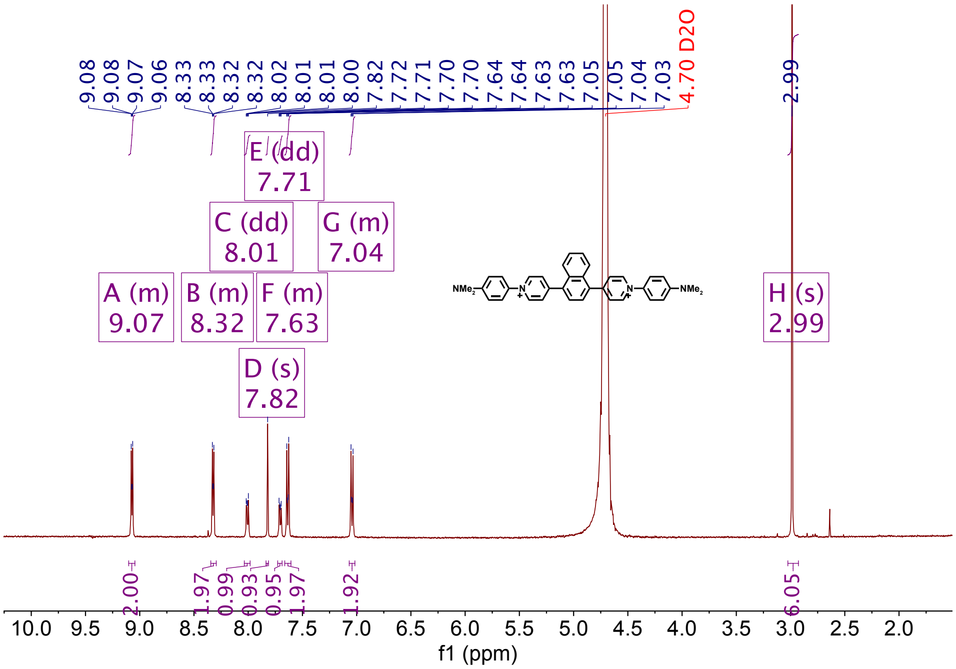

Figure S24: ^{1}H NMR of EV[Np]NM$_2$ (500 MHz, 298.15 K, D_2O)

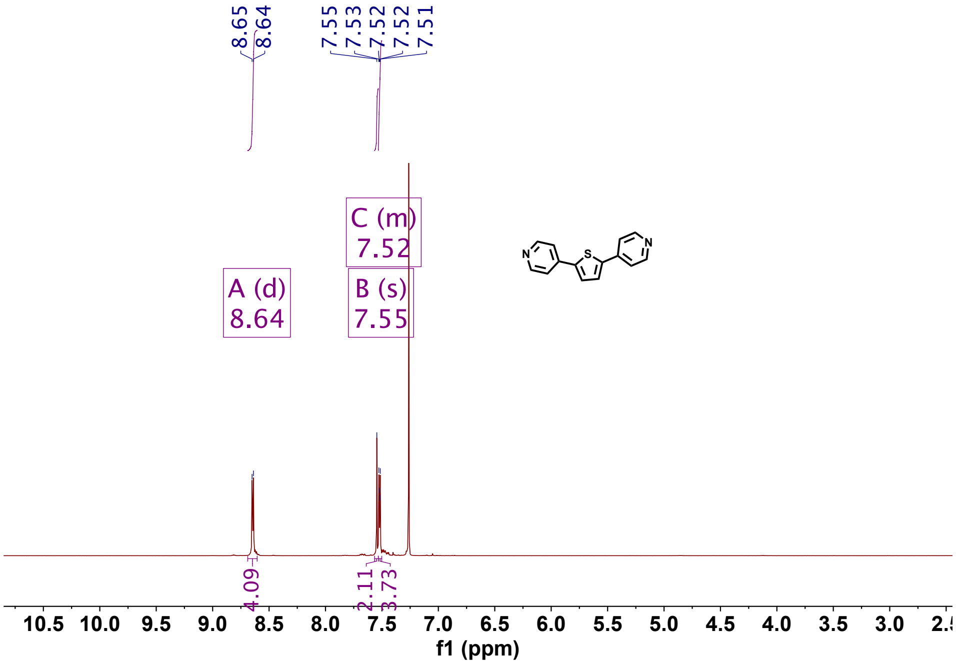

Figure S25: ^{1}H NMR of EV[Th] (500 MHz, 298.15 K, $CDCl_3$)

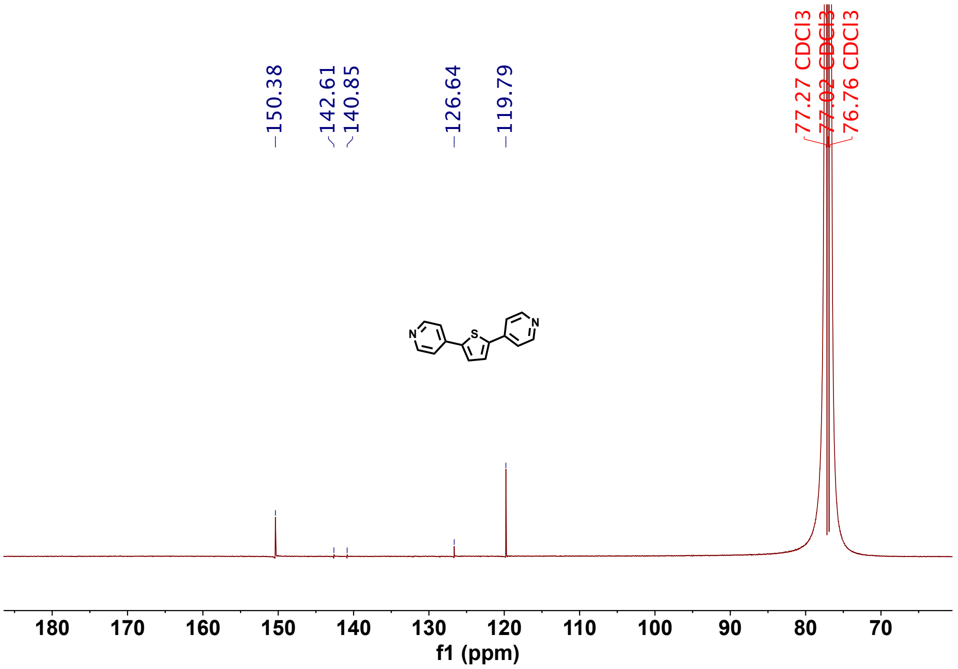

Figure S26: ^{13}C NMR of EV[Th] (126 MHz, 298.15 K, $CDCl_3$)

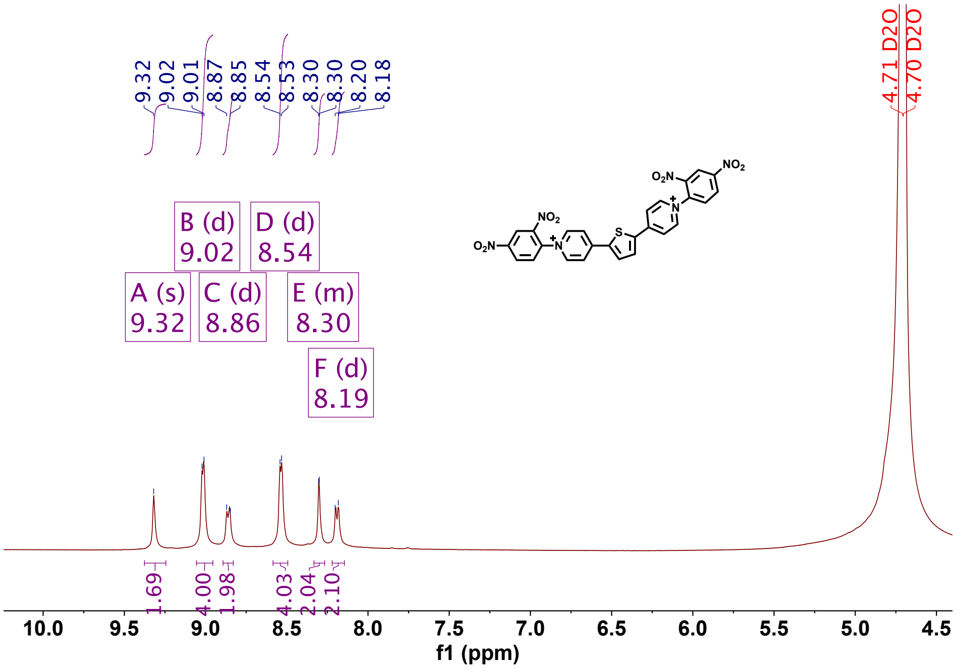

Figure S27: ^{1}H NMR of EV[Th]DNB (500 MHz, 298.15 K, D_2O)

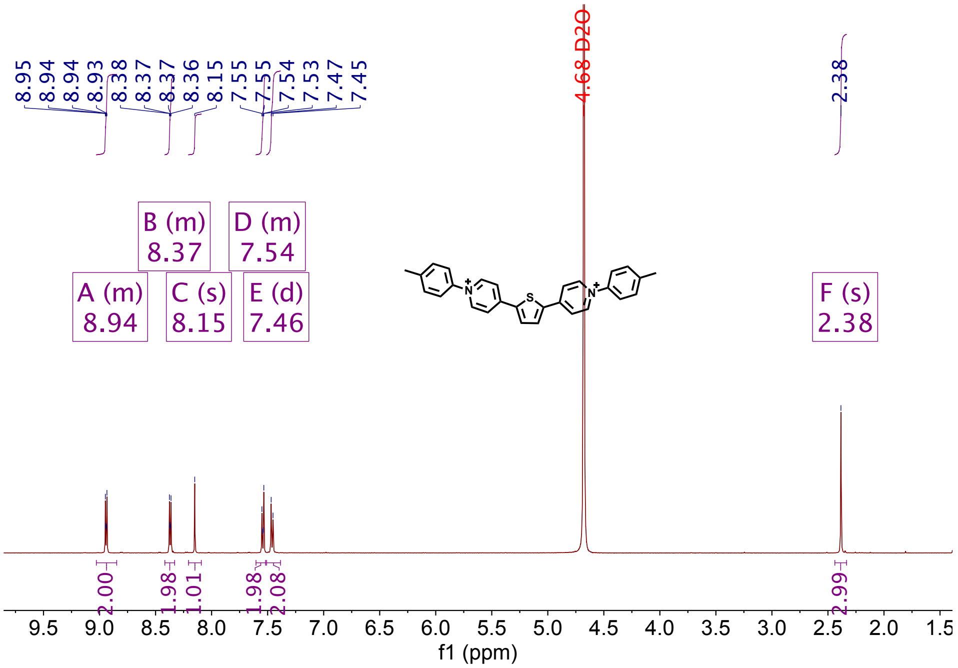

Figure S28: ^{1}H NMR of EV[Th]Me (500 MHz, 298.15 K, D_2O)

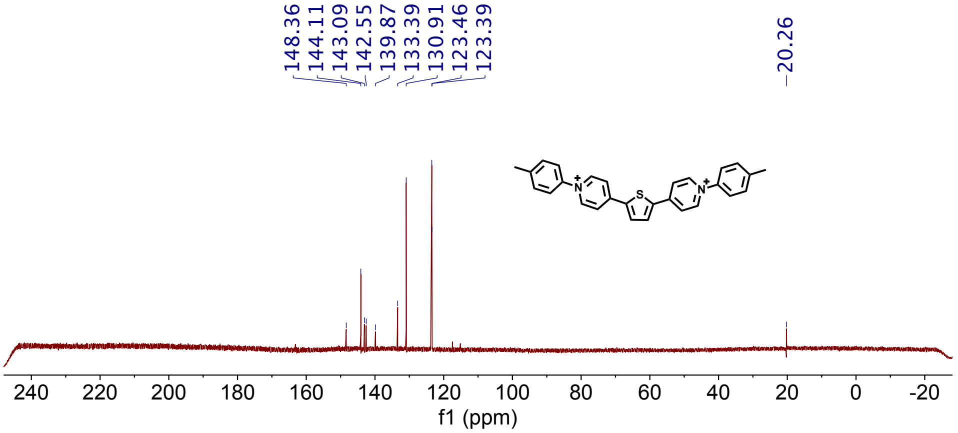

Figure S29: ^{13}C NMR of EV[Th]Me (126 MHz, 298.15 K, $CDCl_3$)

4 High resolution mass spectrometry (HRMS)

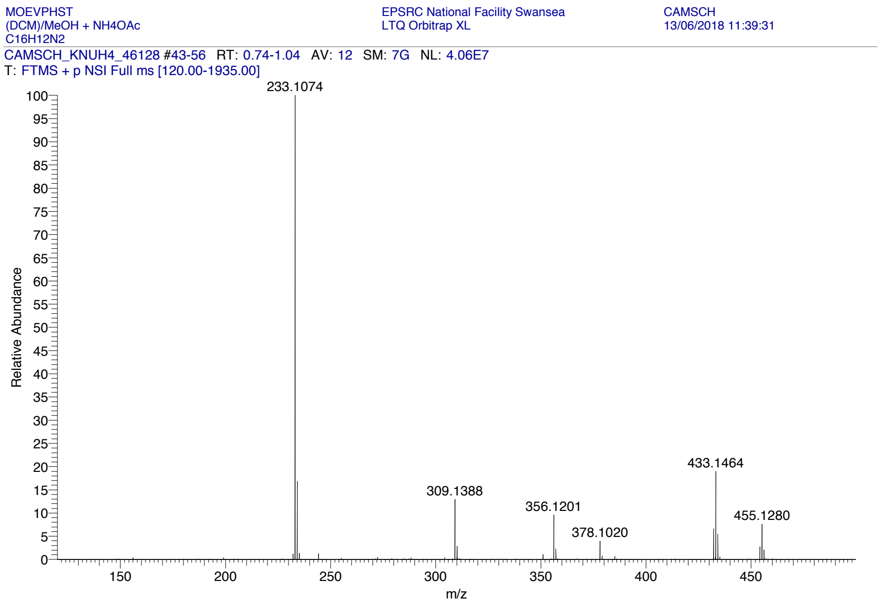

Figure S30: HRMS of EV[Ph]

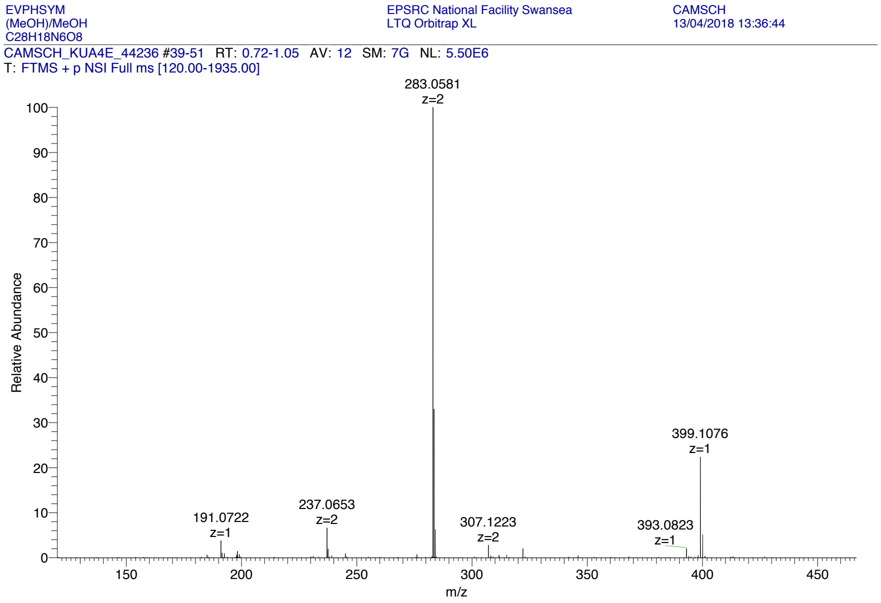

Figure S31: HRMS of EV[Ph]DNB

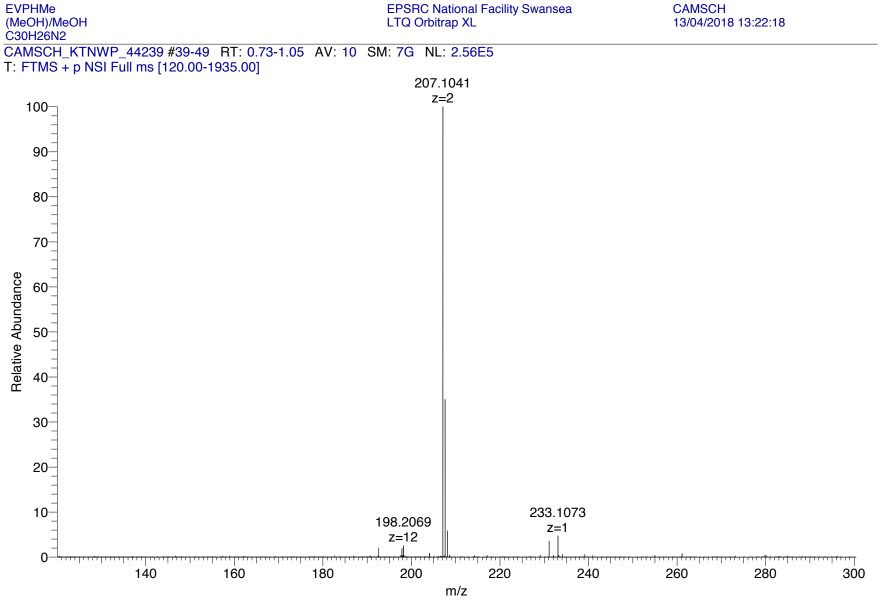

Figure S32: HRMS of EV[Ph]Me

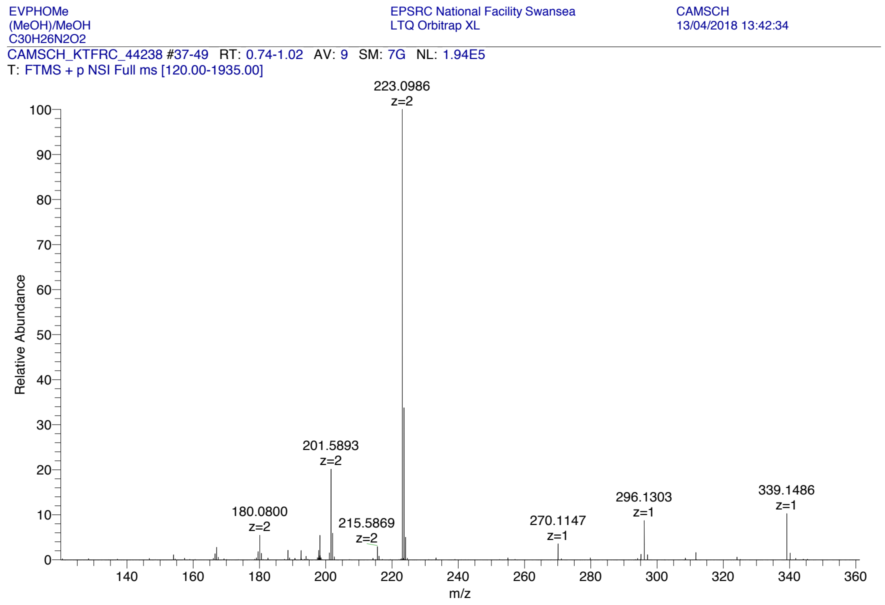

Figure S33: HRMS of EV[Ph]OMe

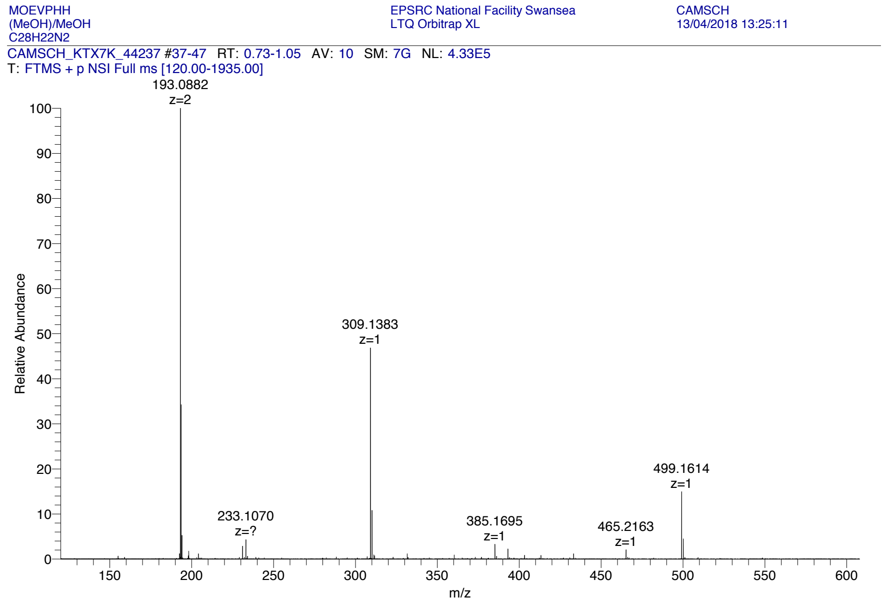

Figure S34: HRMS of EV[Ph]H

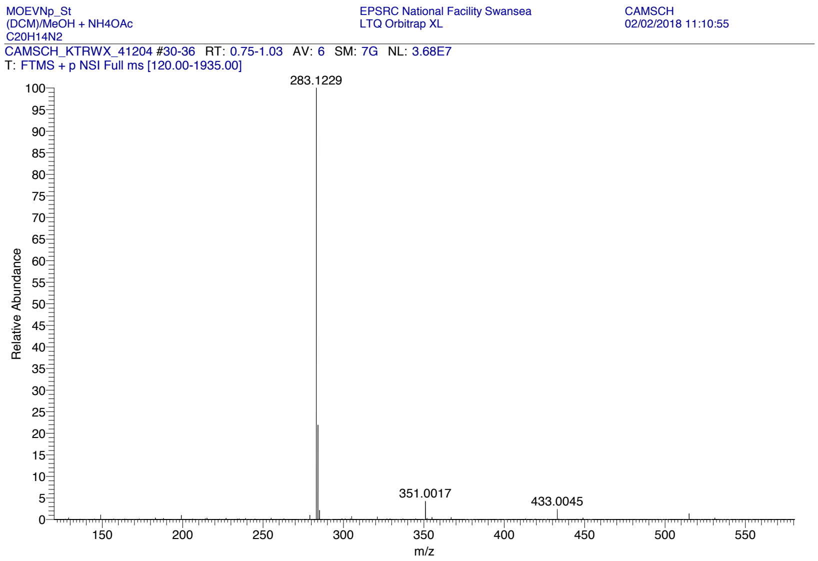

Figure S35: HRMS of EV[Np]

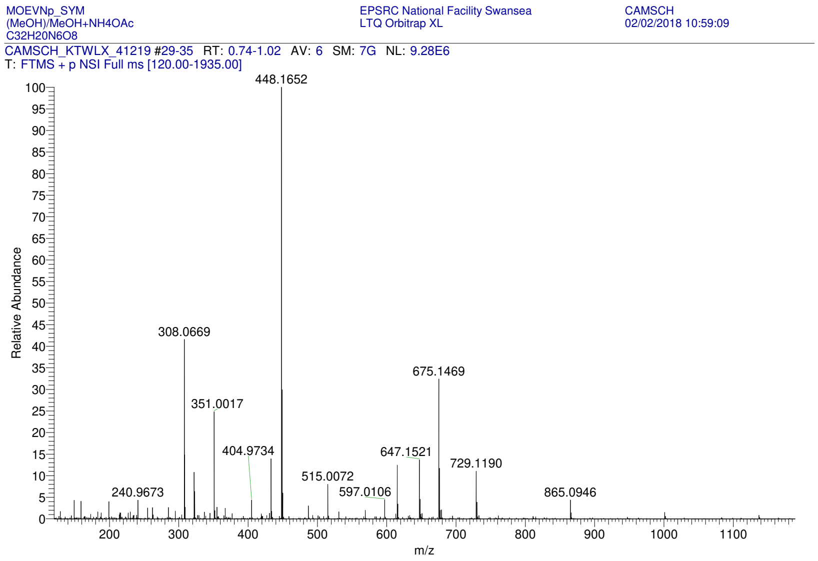

Figure S36: HRMS of EV[Np]DNB

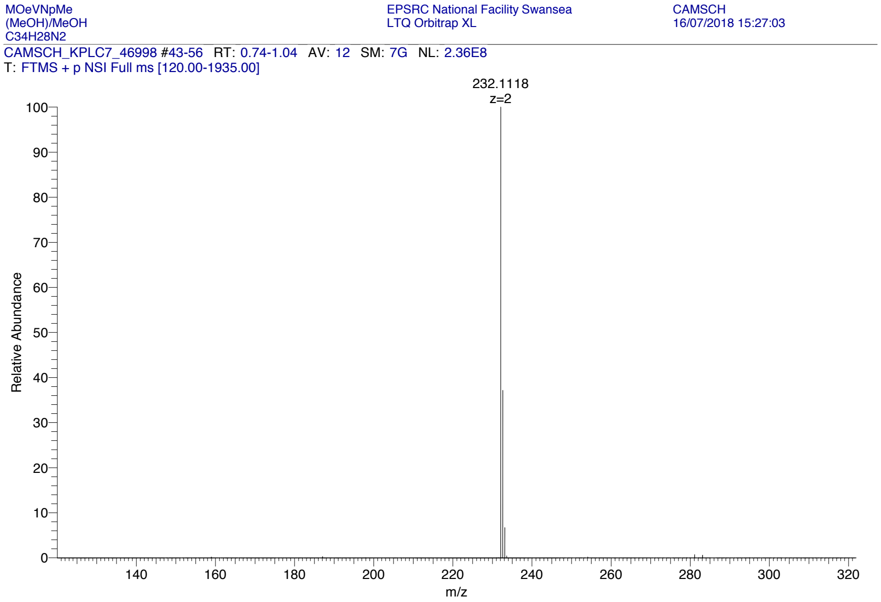

Figure S37: HRMS of EV[Np]Me

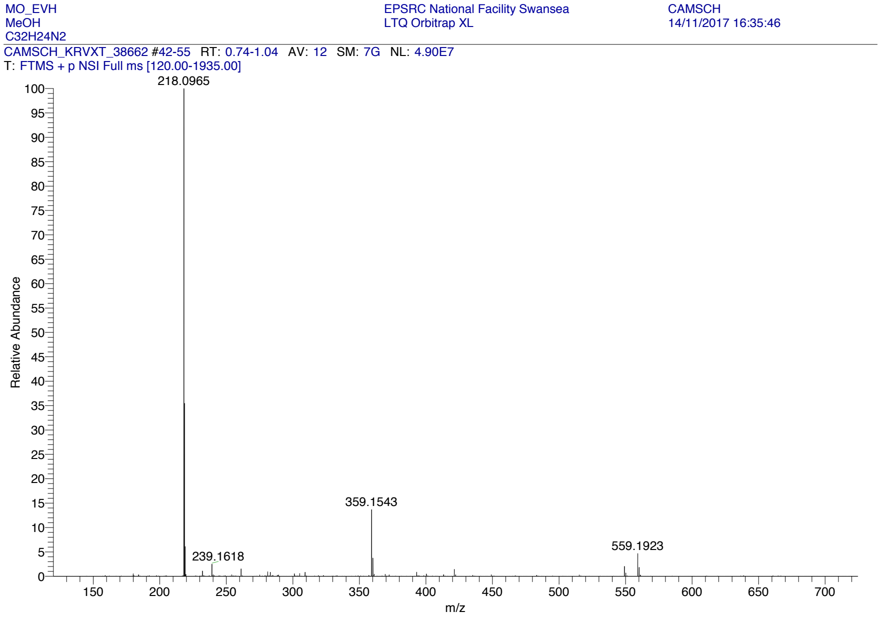

Figure S38: HRMS of EV[Np]H

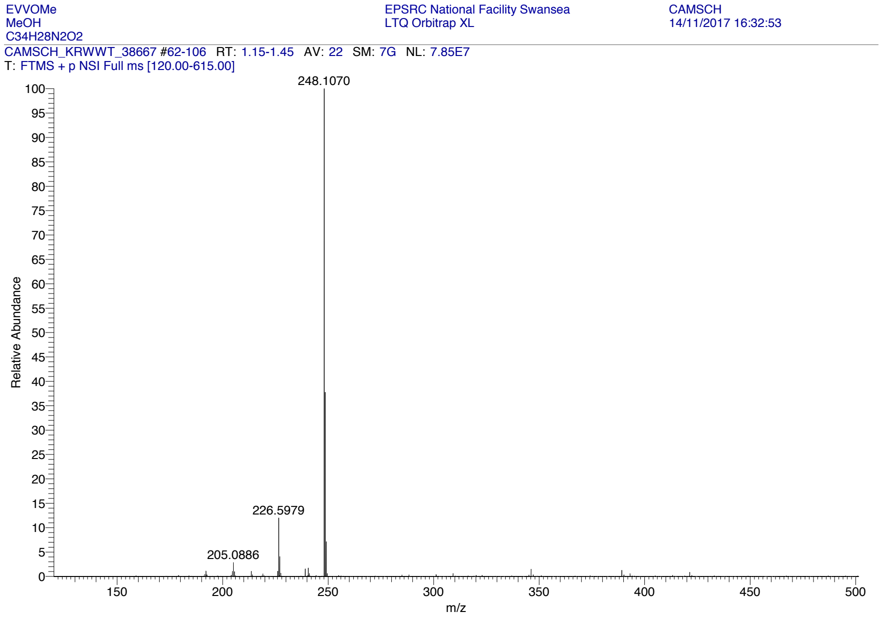

Figure S39: HRMS of EV[Np]OMe

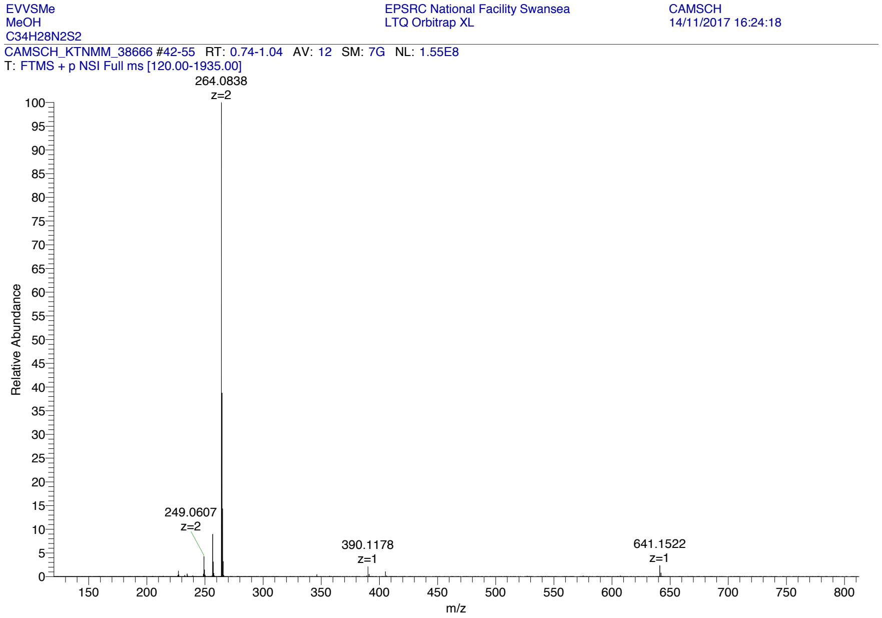

Figure S40: HRMS of EV[Np]SMe

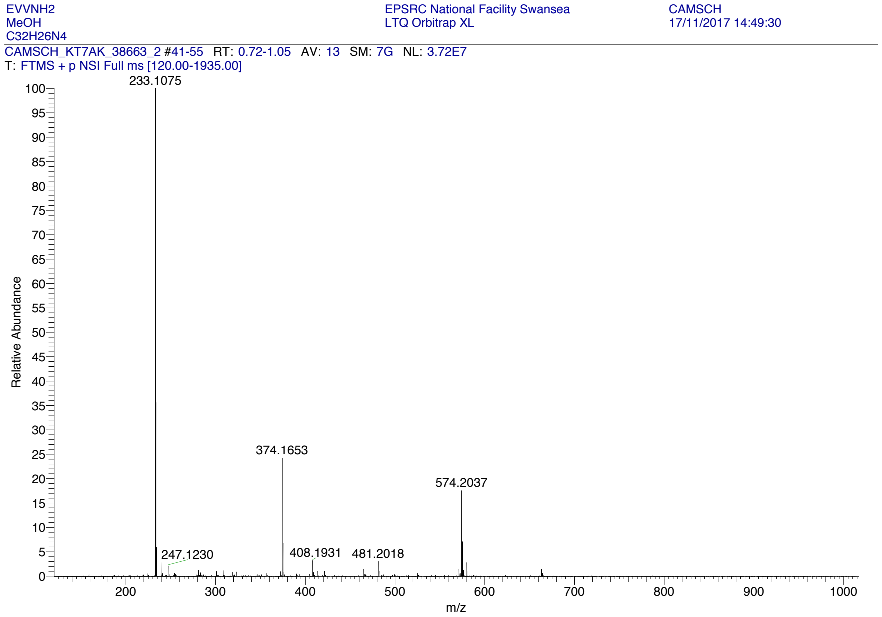

Figure S41: HRMS of EV[Np]NH_2

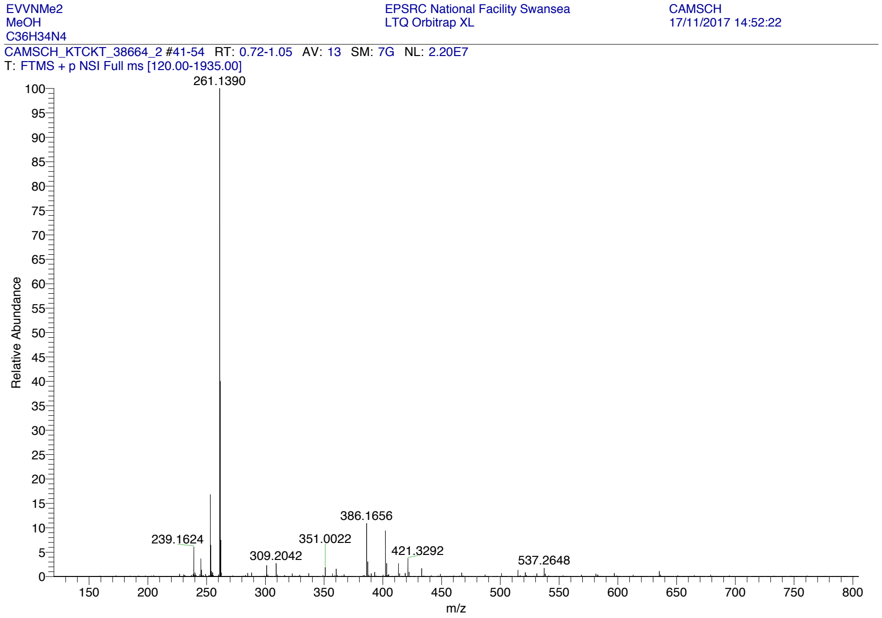

Figure S42: HRMS of EV[Np]NMe_2

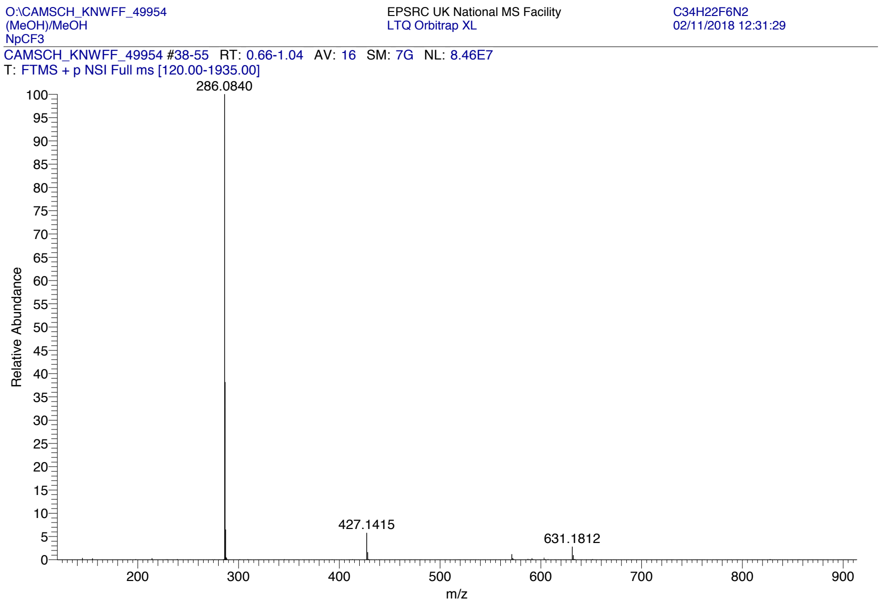

Figure S43: HRMS of EV[Np]CF_3

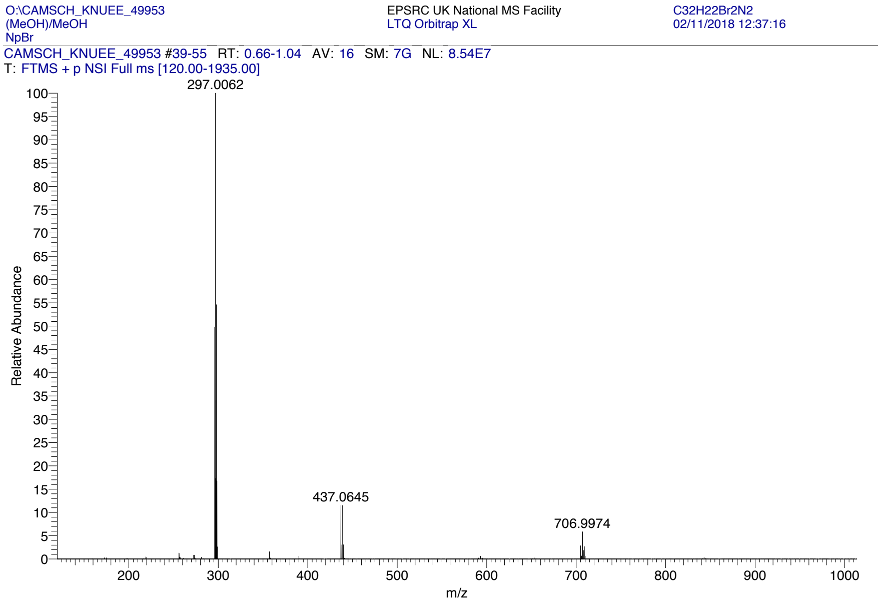

Figure S44: HRMS of EV[Np]Br

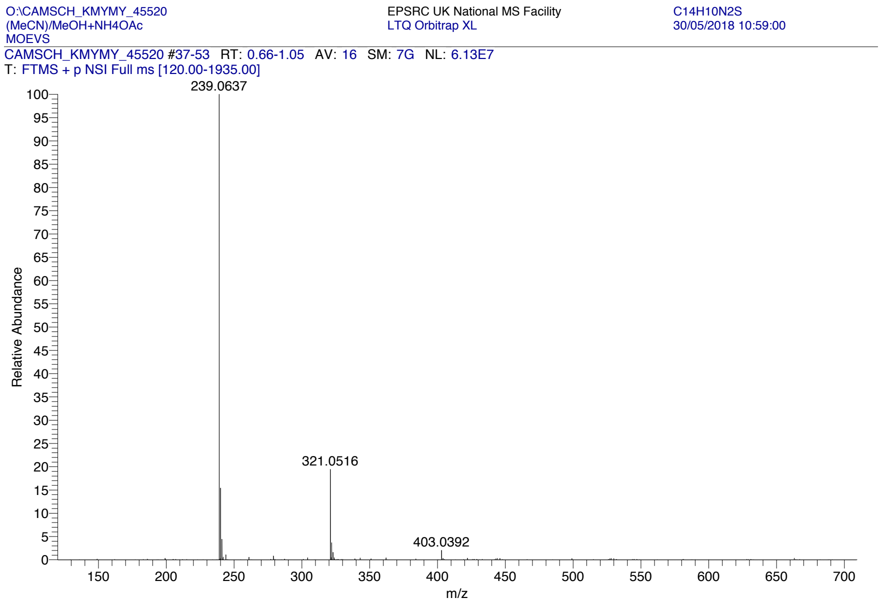

Figure S45: HRMS of EV[Th]

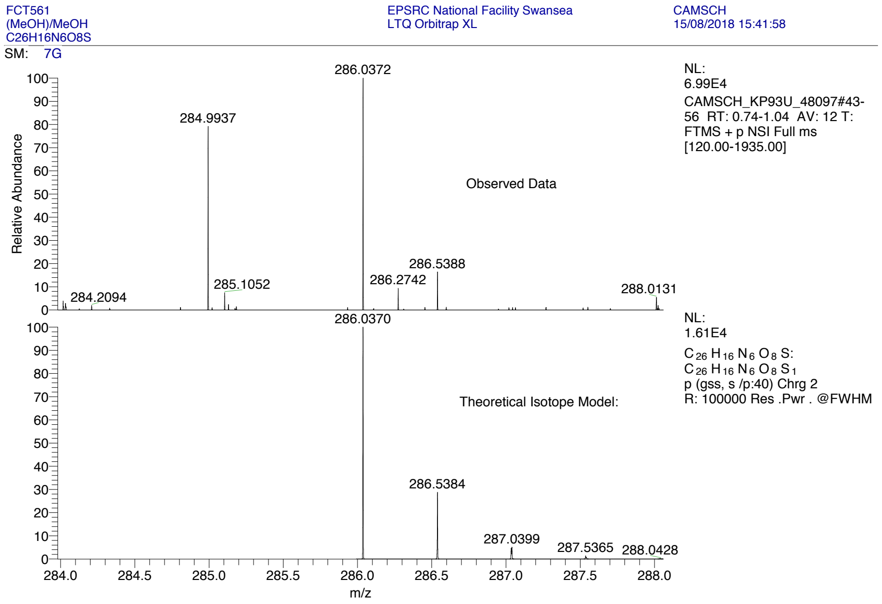

Figure S46: HRMS of EV[Th]DNB

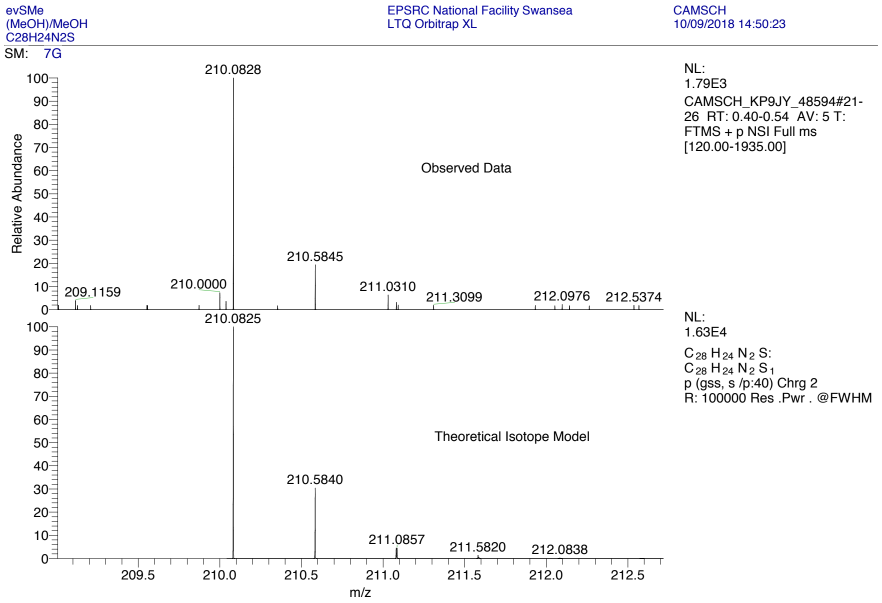

Figure S47: HRMS of EV[Th]Me

5 ^{1}H NMR titration of extended viologens into CB[8] and CB[7]

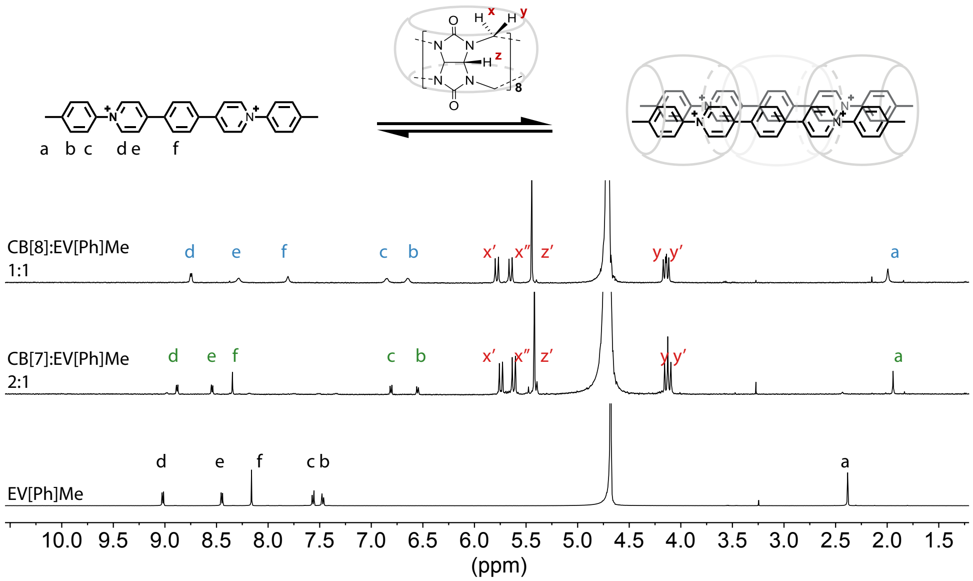

Figure S48: ^{1}H NMR of EV[Ph]Me titrated into CB[7] until a ratio of 2:1 and CB[8] in 1:1 ratio (500 MHz, 298.15 K, D_2O, Cl^- counterions)

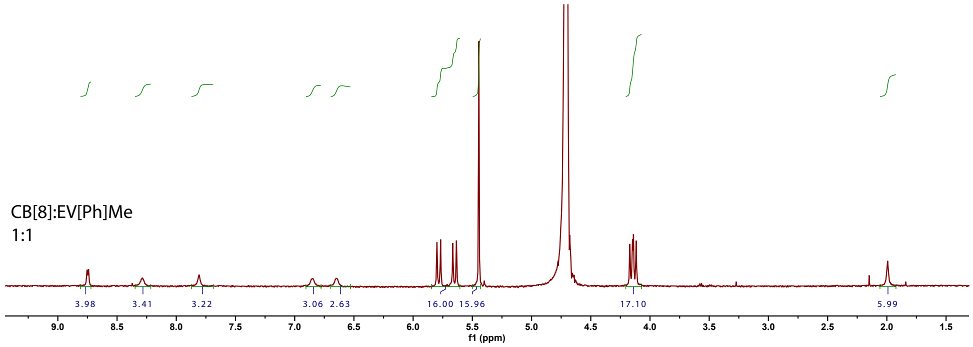

Figure S49: ^{1}H NMR of 1:1 host-guest ratio mixture of EV[Ph]Me and CB[8] (500 MHz, 298.15 K, D_2O, Cl^- counterions)

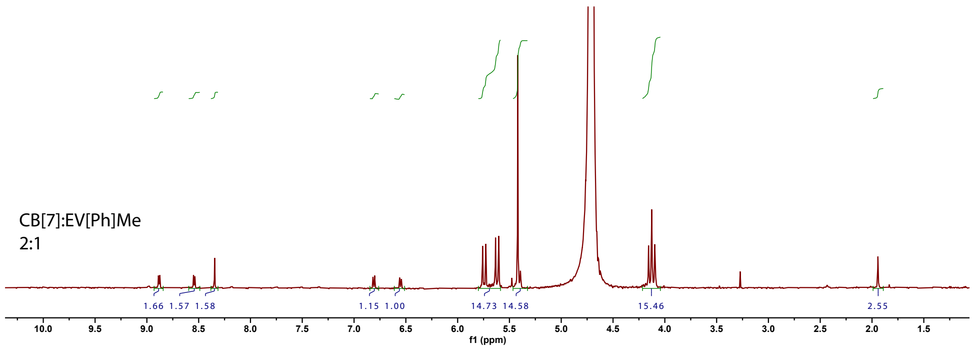

Figure S50: ^{1}H NMR of 2:1 host-guest ratio mixture of EV[Ph]Me and CB[7] (500 MHz, 298.15 K, D_2O, Cl^- counterions)

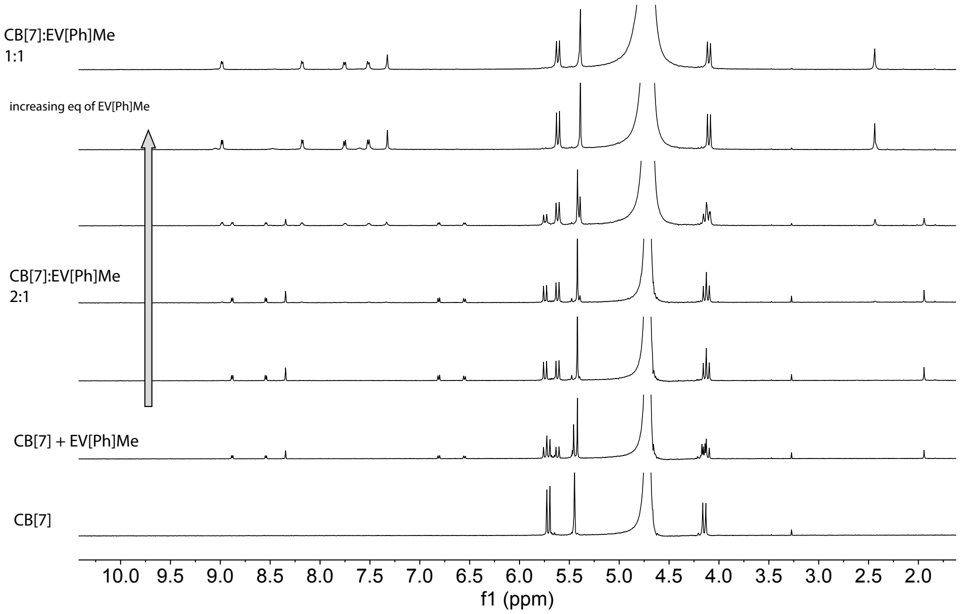

Figure S51: ^{1}H NMR titration of EV[Ph]Me into CB[7] (500 MHz, 298.15 K, D_2O, Cl^- counterions)

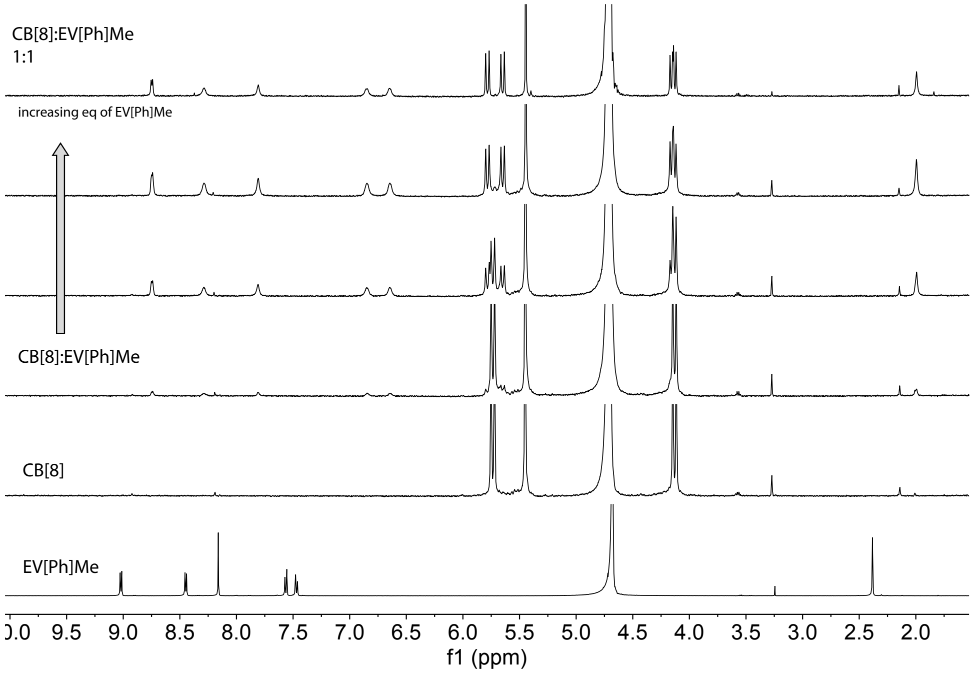

Figure S52: ^{1}H NMR titration of EV[Ph]Me into CB[8] (500 MHz, 298.15 K, D_2O, Cl^- counterions)

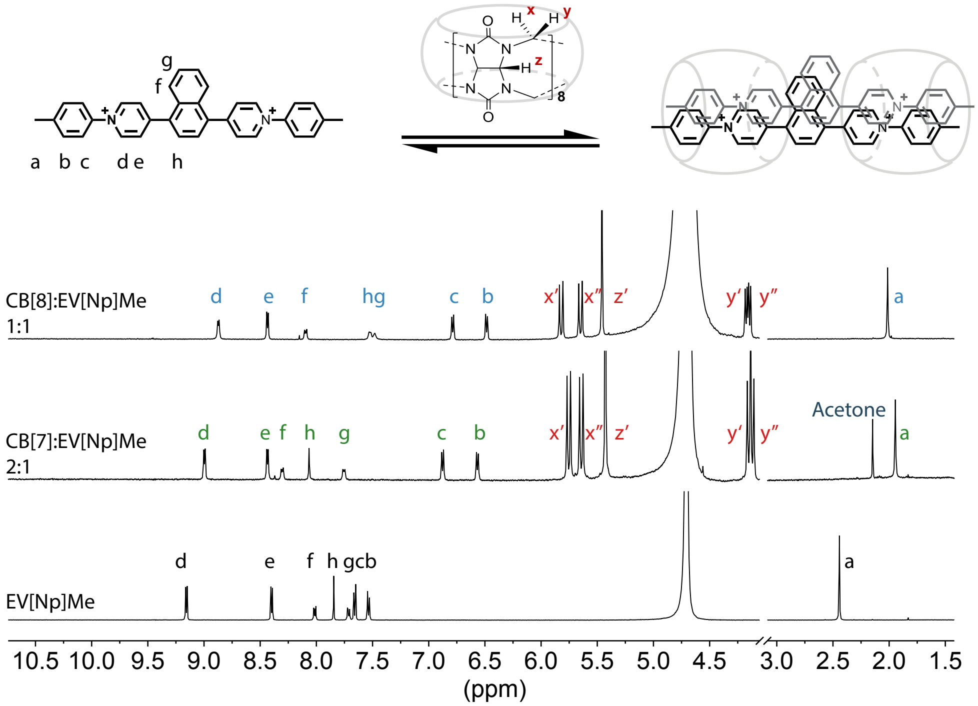

Figure S53: ^{1}H NMR of EV[Np]Me titrated into CB[7] until a ratio of 2:1 and CB[8] in 1:1 ratio (500 MHz, 298.15 K, D_2O, Cl^- counterions)

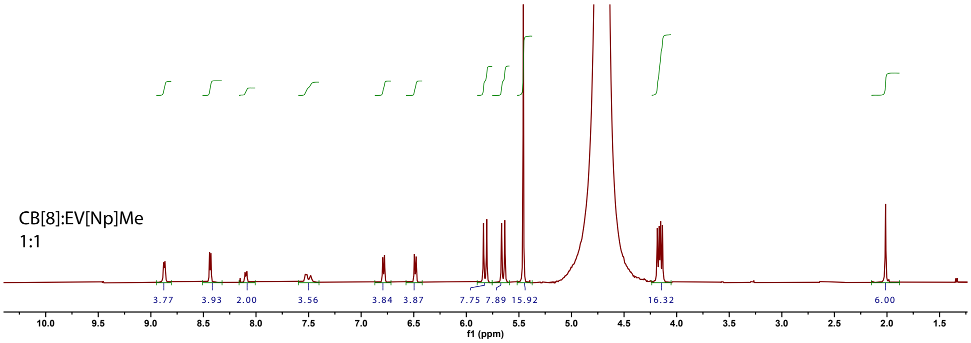

Figure S54: ^{1}H NMR of 1:1 host-guest ratio mixture of EV[Np]Me and CB[8] (500 MHz, 298.15 K, D_2O, Cl^- counterions)

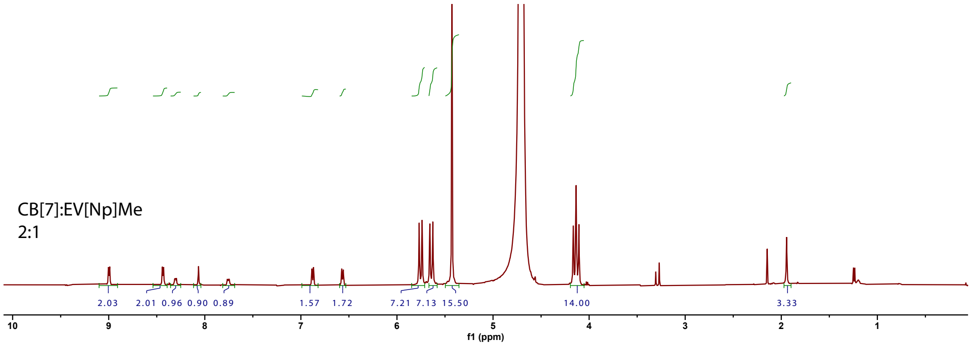

Figure S55: ^{1}H NMR of 2:1 host-guest ratio mixture of EV[Np]Me and CB[7] (500 MHz, 298.15 K, D_2O, Cl^- counterions)

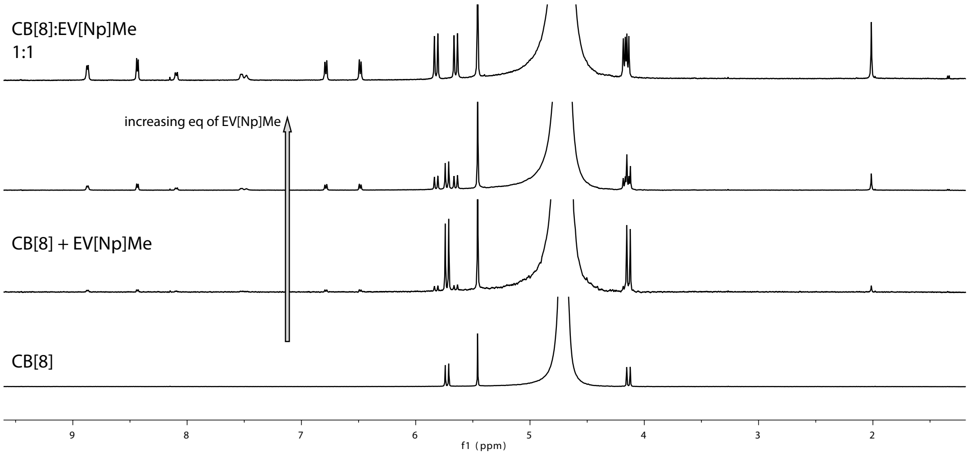

Figure S56: ^{1}H NMR titration of EV[Np]Me into CB[8] (500 MHz, 298.15 K, D_2O, Cl^- counterions)

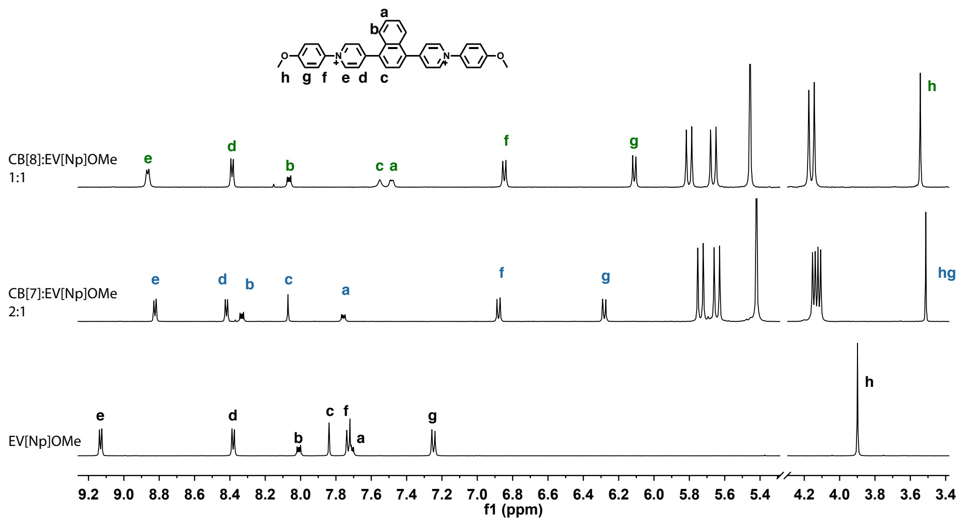

Figure S57: ^{1}H NMR of EV[Np]OMe titrated into CB[7] until a ratio of 2:1 and CB[8] in 1:1 ratio (500 MHz, 298.15 K, D_2O, Cl^- counterions)

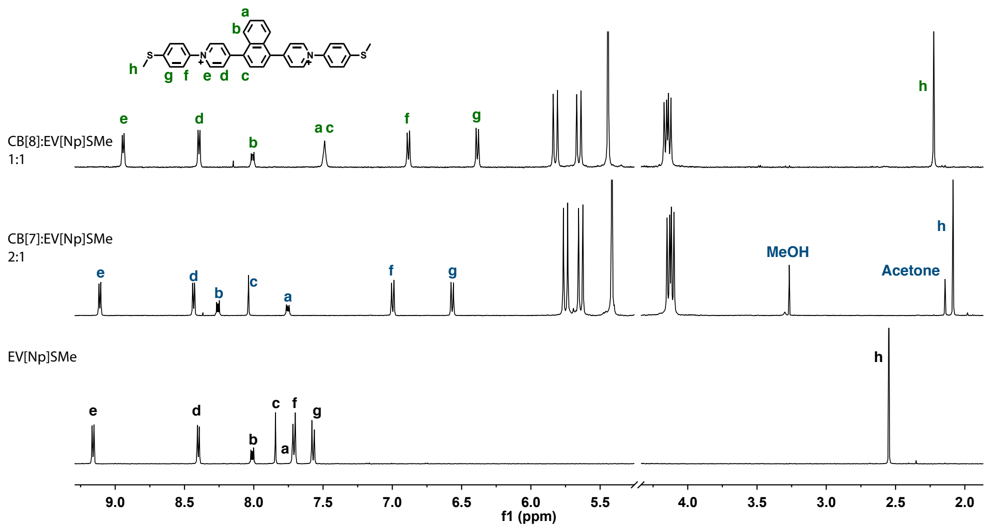

Figure S58: ^{1}H NMR of EV[Np]SMe titrated into CB[7] until a ratio of 2:1 and CB[8] in 1:1 ratio (500 MHz, 298.15 K, D_2O, Cl^- counterions)

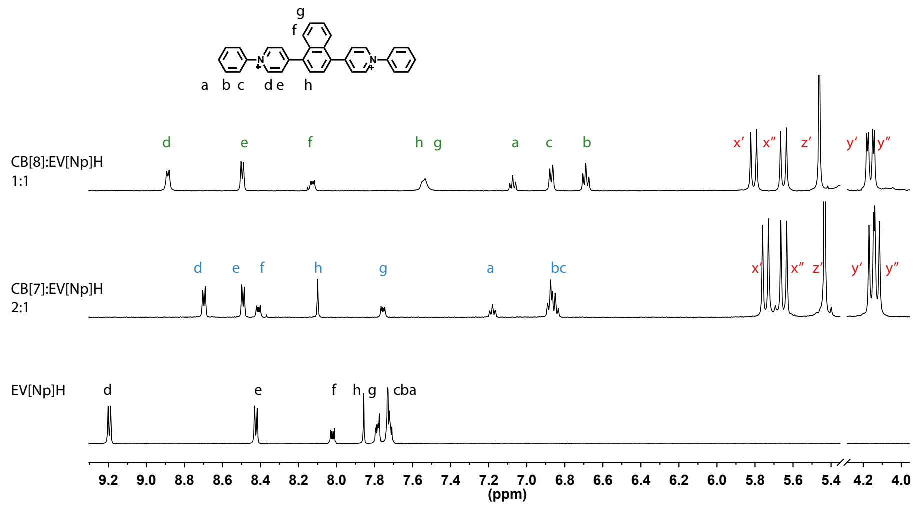

Figure S59: ^{1}H NMR of EV[Np]H titrated into CB[7] until a ratio of 2:1 and CB[8] in 1:1 ratio (500 MHz, 298.15 K, D_2O, Cl^- counterions)

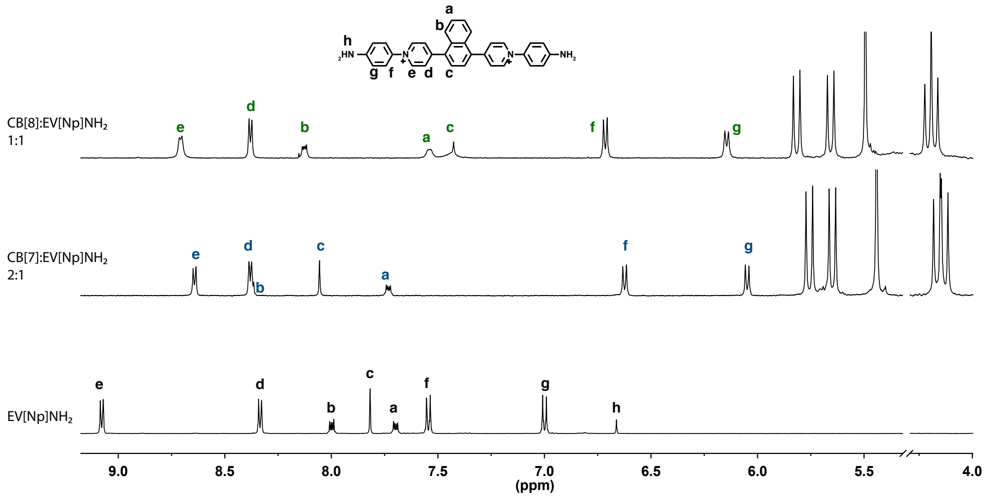

Figure S60: ^{1}H NMR of EV[Np]NH_2 titrated into CB[7] until a ratio of 2:1 and CB[8] in 1:1 ratio (500 MHz, 298.15 K, D_2O, Cl^- counterions)

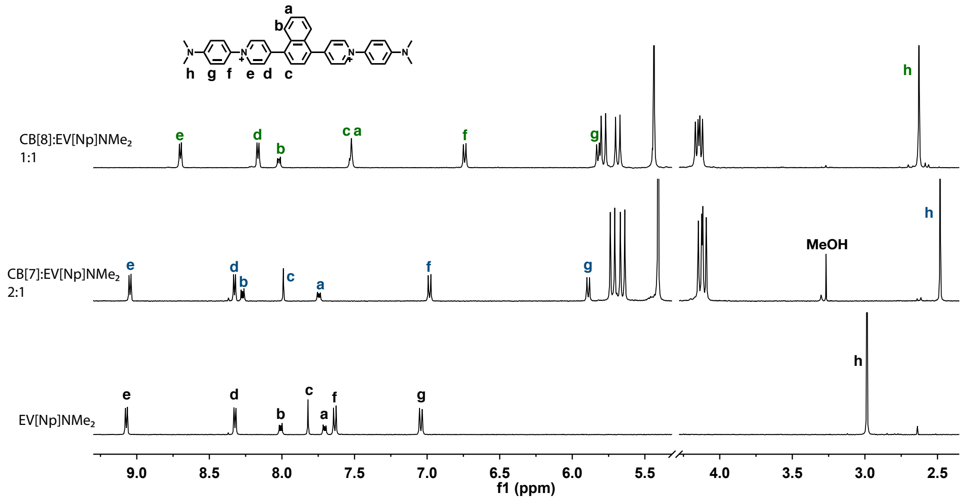

Figure S61: ^{1}H NMR of EV[Np]NMe$_2$ titrated into CB[7] until a ratio of 2:1 and CB[8] in 1:1 ratio (500 MHz, 298.15 K, D$_2$O, Cl$^-$ counterions)

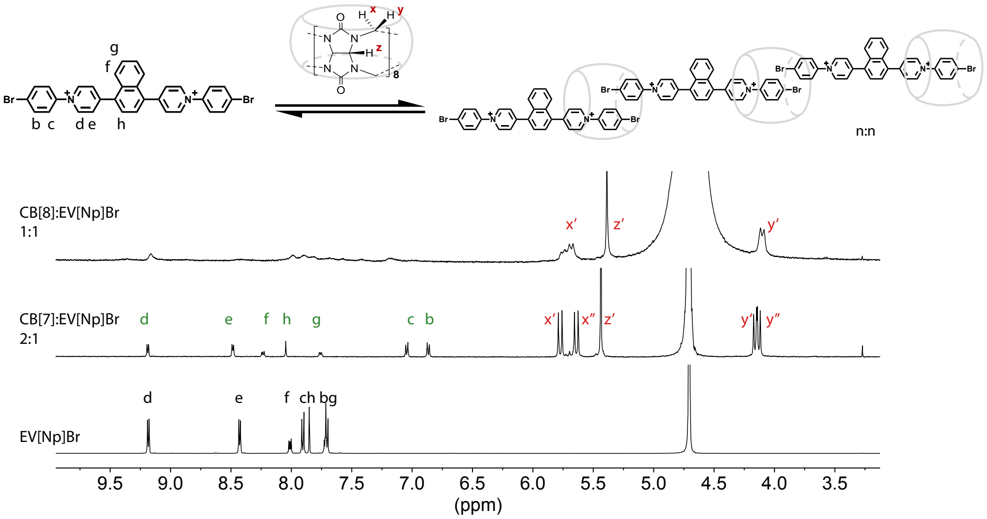

Figure S62: ^{1}H NMR of EV[Np]Br titrated into CB[7] until a ratio of 2:1 and CB[8] in 1:1 ratio (500 MHz, 298.15 K, D$_2$O, Cl$^-$ counterions)

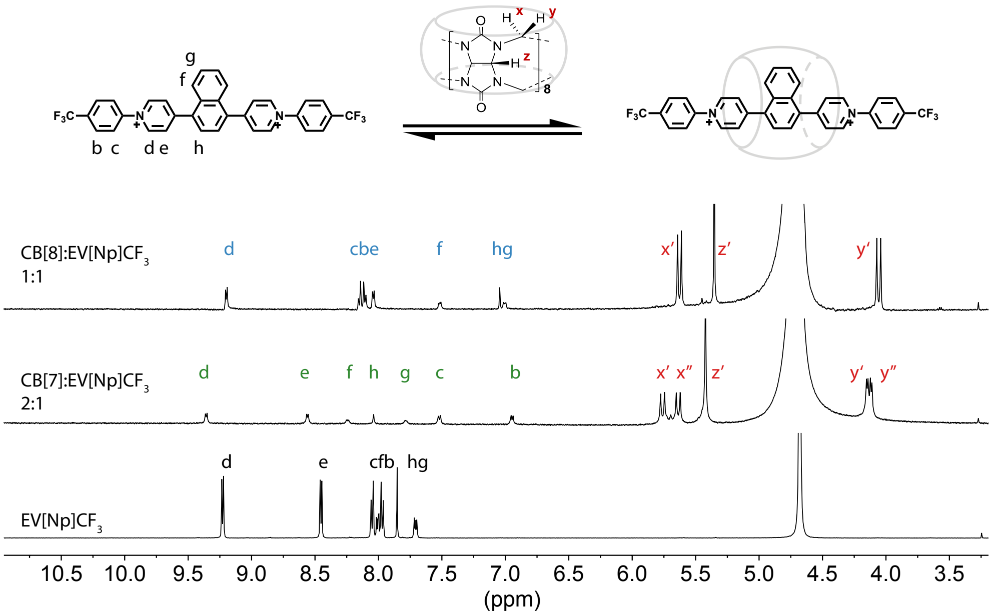

Figure S63: ^{1}H NMR of EV[Np]CF$_3$ titrated into CB[7] until a ratio of 2:1 and CB[8] in 1:1 ratio (500 MHz, 298.15 K, D_2O, Cl^- counterions)

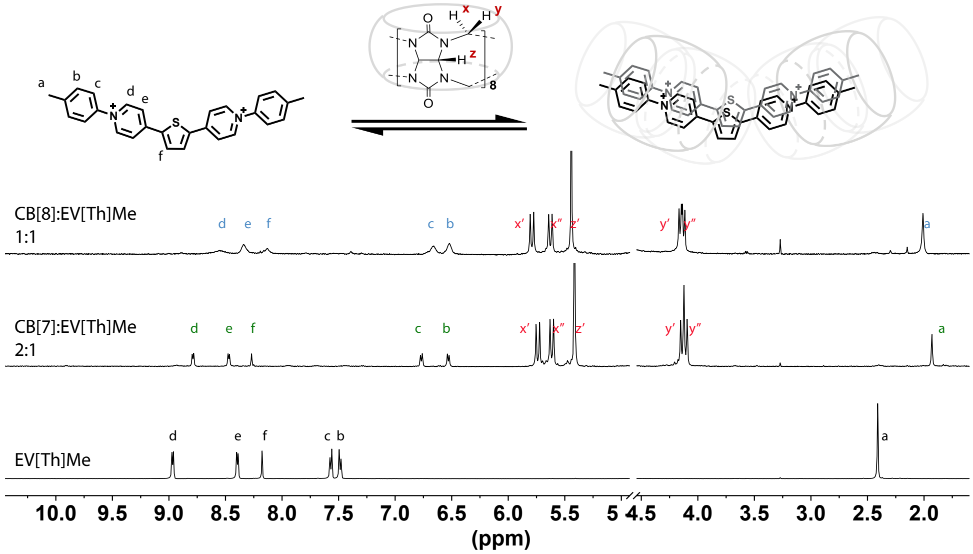

Figure S64: ^{1}H NMR of EV[Th]Me titrated into CB[7] until a ratio of 2:1 and CB[8] in 1:1 ratio (500 MHz, 298.15 K, D_2O, Cl^- counterions)

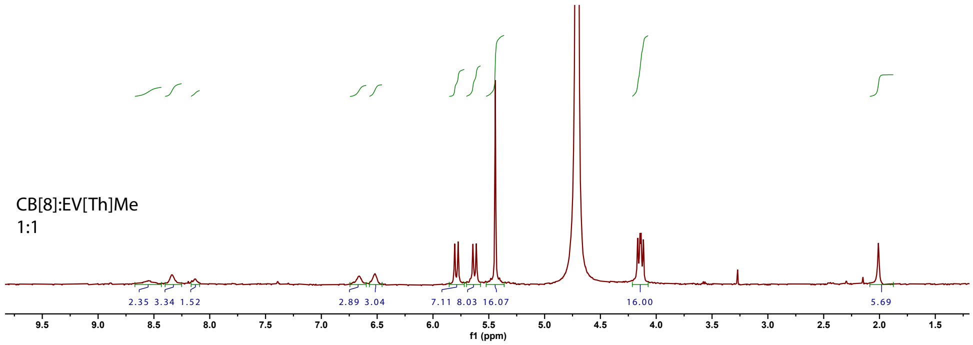

Figure S65: ^{1}H NMR of 1:1 host-guest ratio mixture of EV[Th]Me and CB[8] (500 MHz, 298.15 K, D_2O, Cl^- counterions)

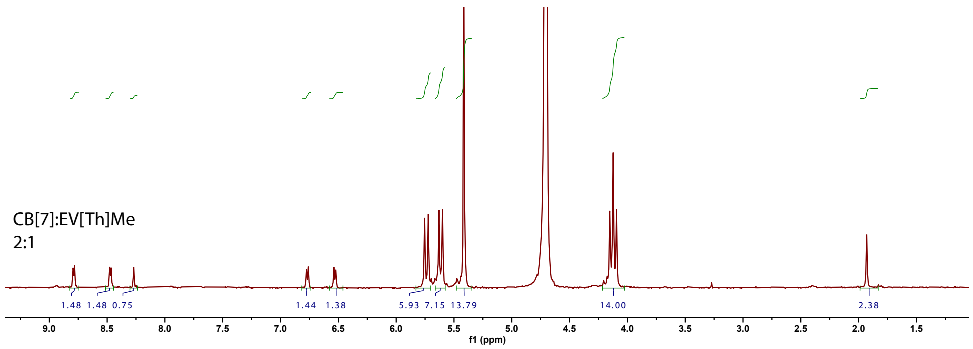

Figure S66: ^{1}H NMR of 2:1 host-guest ratio mixture of EV[Th]Me and CB[7] (500 MHz, 298.15 K, D_2O, Cl^- counterions)

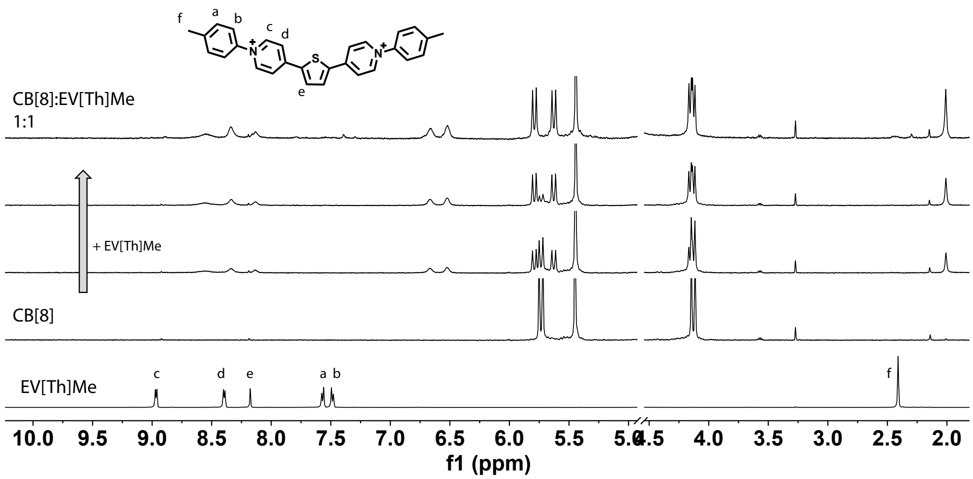

Figure S67: ^{1}H NMR titration of EV[Th]Me into CB[8] (500 MHz, 298.15 K, D_2O, Cl^- counterions)

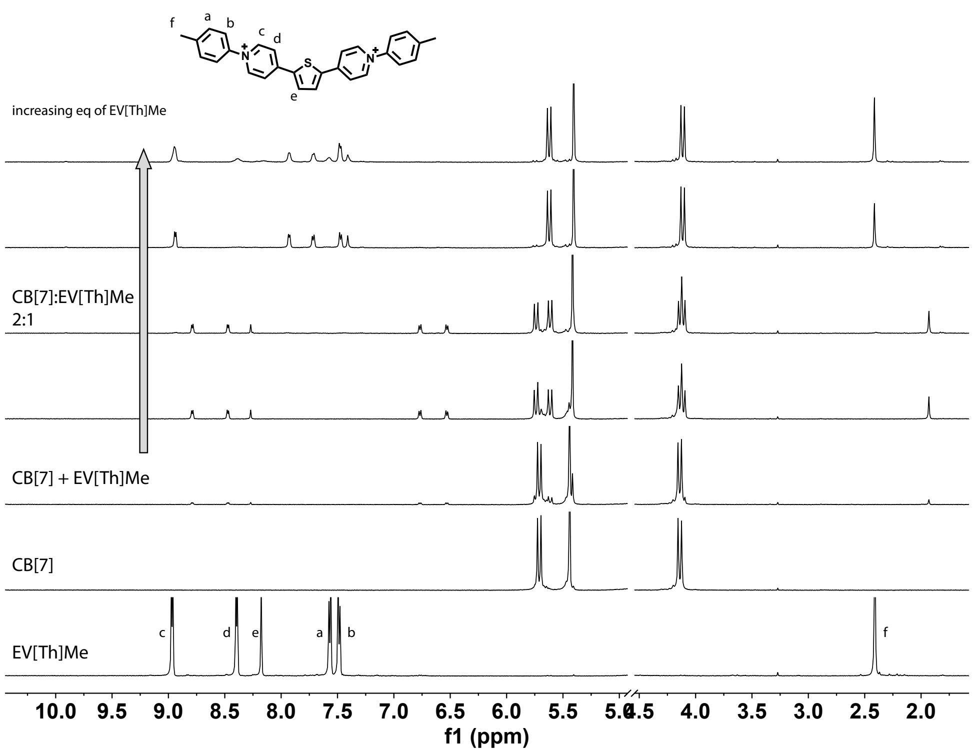

Figure S68: ^{1}H NMR titration of EV[Th]Me into CB[7] (500 MHz, 298.15 K, D_2O, Cl^- counterions)

6 Isothermal titration thermograms

Table S1: Thermodynamic data of EV[X]R binding with CB[8] (T=298.15 K, 50 mM sodium acetate buffer pH = 4.75).

	ΔG (kJ mol^{-1})	ΔH (kJ mol^{-1})	$T\Delta S$ (kJ mol^{-1})	K (M^{-1})
EV[Ph]Me	-38.5	-95.2	-56.7	5.49×10^6
EV[Np]Me	-42.7	-67.6	-24.7	3.21×10^7
EV[Th]Me	-43.3	-53.7	-10.4	3.78×10^7
EV[Np]H	-41.5	-56.1	-14.6	1.86×10^7
EV[Np]OMe	-42.7	-58.3	-15.6	2.97×10^7
EV[Np]SMe	-43.3	-68.3	-25.0	3.78×10^7
EV[Np]NMe$_2$	-42.5	-59.0	-16.6	2.77×10^7
EV[Np]NH$_2$	-40.7	-71.1	-30.5	1.32×10^7

Table S2: Thermodynamic data of EV[X]R binding with CB[7] (T=298.15 K, 50 mM sodium acetate buffer pH = 4.75).

	ΔG (kJ mol^{-1})	ΔH (kJ mol^{-1})	$T\Delta S$ (kJ mol^{-1})	K (M^{-1})
EV[Ph]Me	-35.5	-36.9	-1.4	1.65×10^6
EV[Np]Me	-38.5	-32.0	6.5	5.45×10^6
EV[Th]Me	-36.7	-17.0	19.8	2.72×10^6

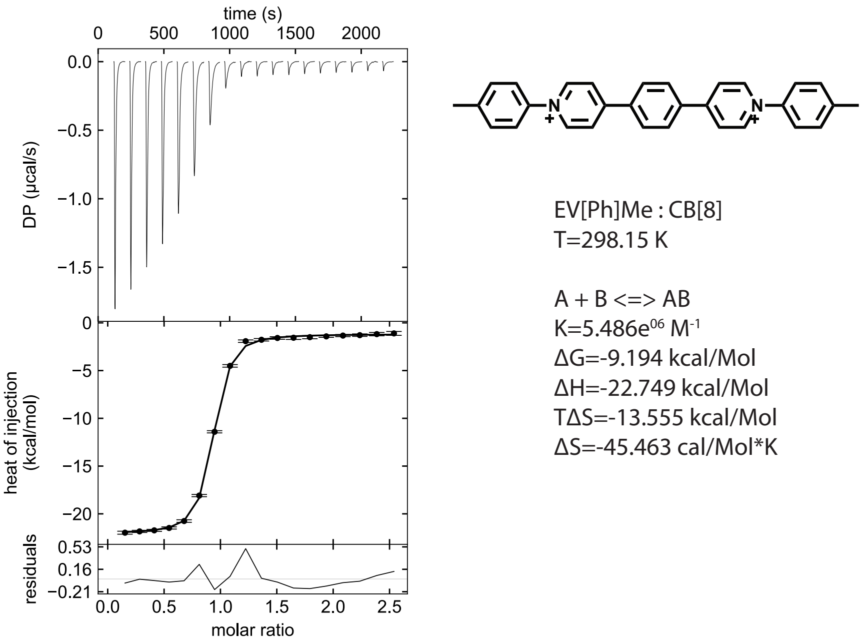

Figure S69: ITC titration of 0.7 mM EV[Ph]Me to 0.051 mM CB[8] (T=298.15 K, 50 mM sodium acetate buffer pH = 4.75, Cl^- counterions)

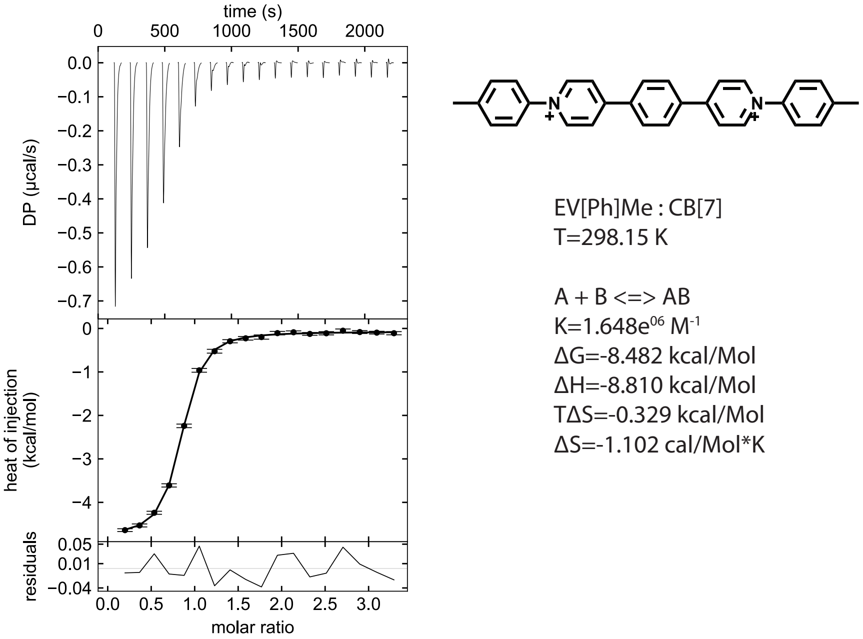

Figure S70: ITC titration of 1 mM EV[Ph]Me to 0.061 mM CB[7] (T=298.15 K, 50 mM sodium acetate buffer pH = 4.75, Cl^- counterions)

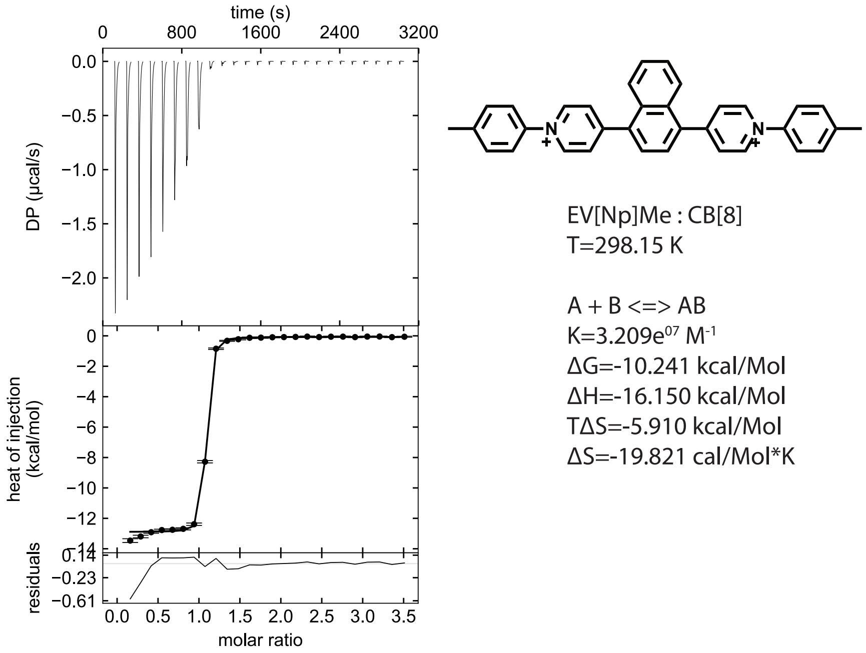

Figure S71: ITC titration of 0.8 mM EV[Np]Me to 0.051 mM CB[8] (T=298.15 K, 50 mM sodium acetate buffer pH = 4.75, Cl^- counterions)

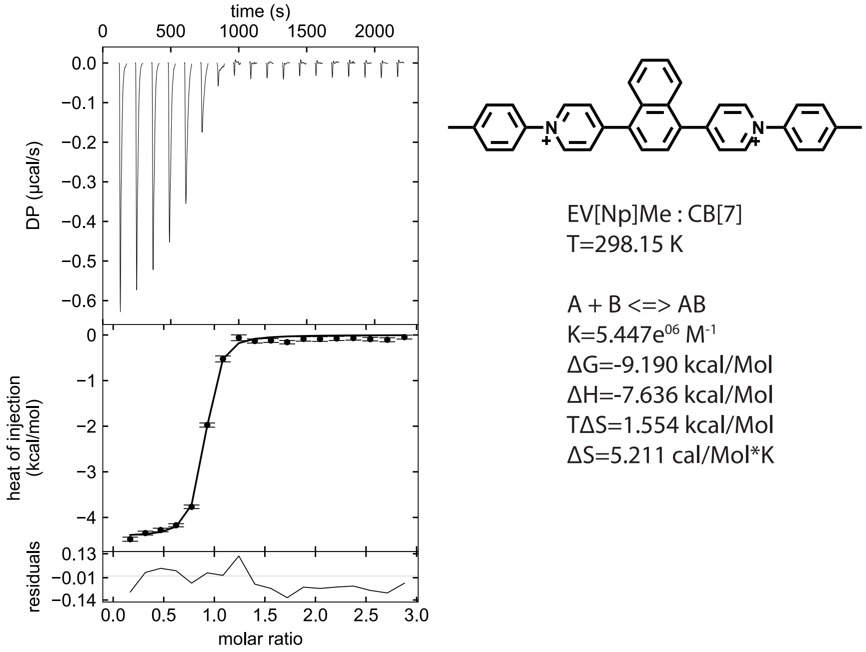

Figure S72: ITC titration of 1.2 mM EV[Np]Me to 0.061 mM CB[7] (T=298.15 K, 50 mM sodium acetate buffer pH = 4.75, Cl^- counterions)

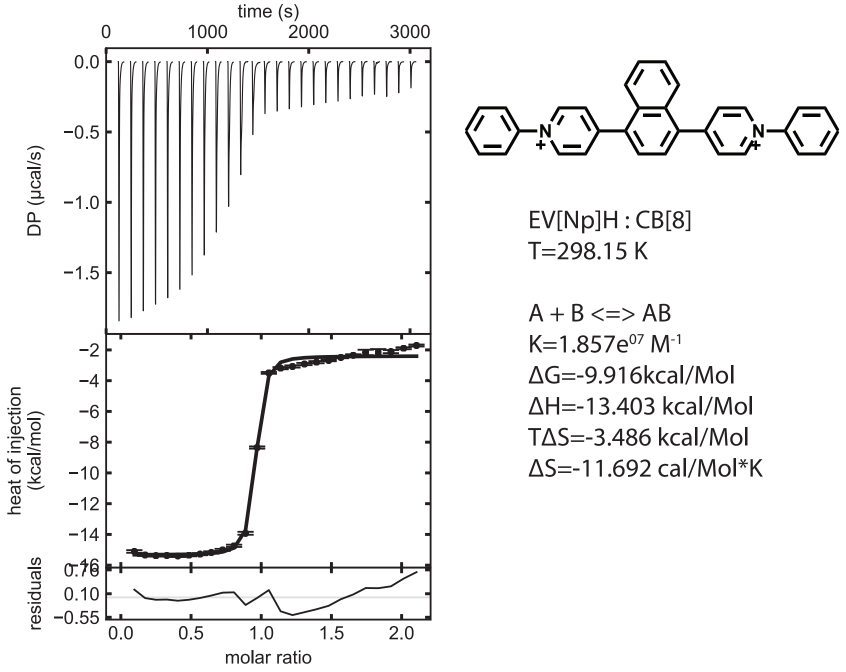

Figure S73: ITC titration of 0.6 mM EV[Np]H to 0.051 mM CB[8] (T=298.15 K, 50 mM sodium acetate buffer pH = 4.75, Cl^- counterions)

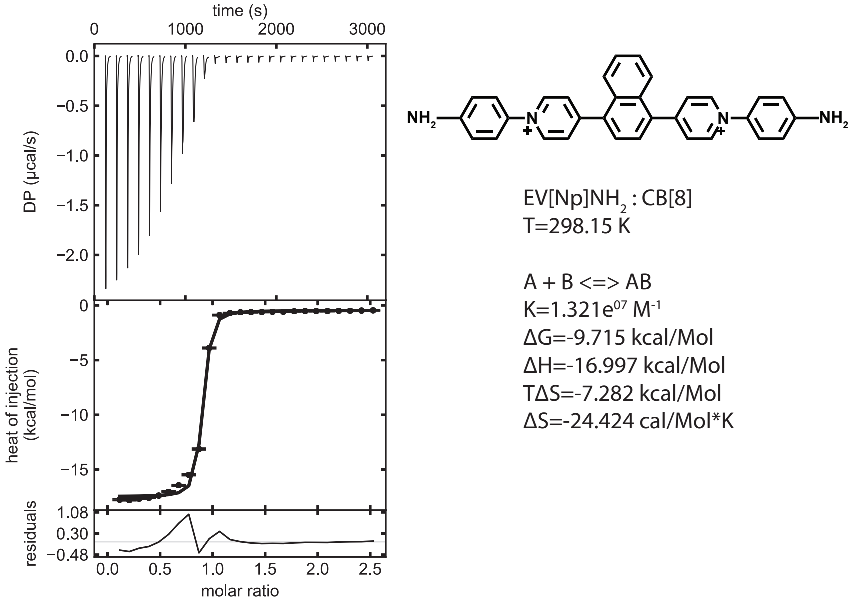

Figure S74: ITC titration of 1 mM EV[Np]NH_2 to 0.051 mM CB[8] (T=298.15 K, 50 mM sodium acetate buffer pH = 4.75, Cl^- counterions)

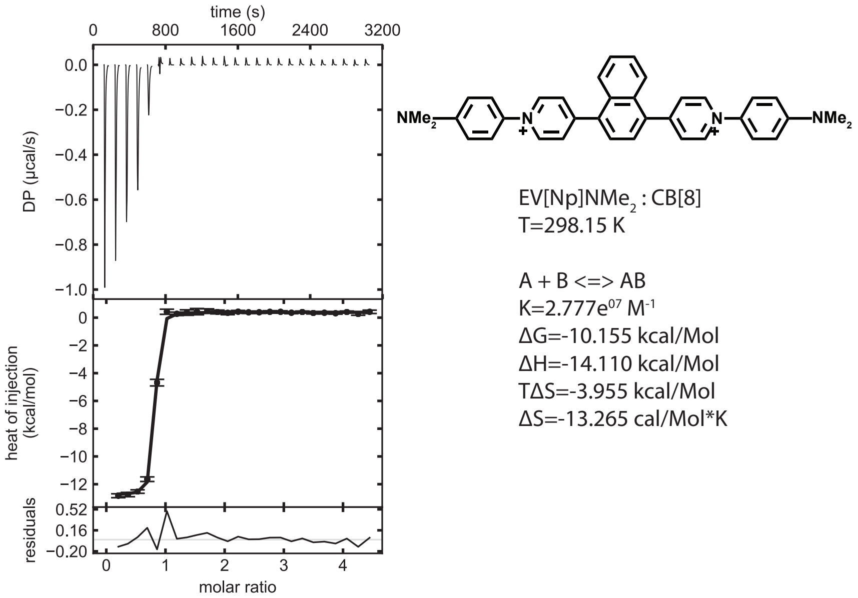

Figure S75: ITC titration of 1.1 mM EV[Np]NMe_2 to 0.051 mM CB[8] (T=298.15 K, 50 mM sodium acetate buffer pH = 4.75, Cl^- counterions)

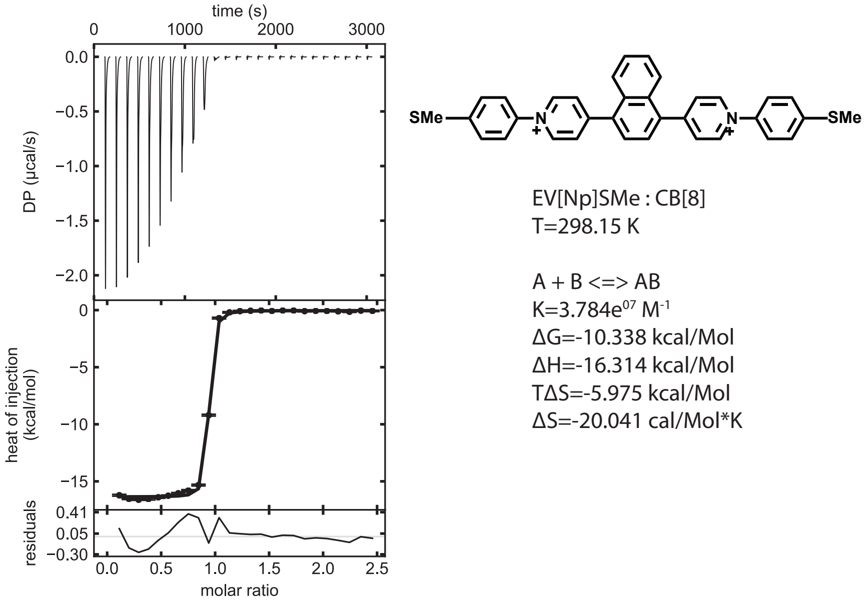

Figure S76: ITC titration of 1 mM EV[Np]SMe to 0.051 mM CB[8] (T=298.15 K, 50 mM sodium acetate buffer pH = 4.75, Cl^- counterions)

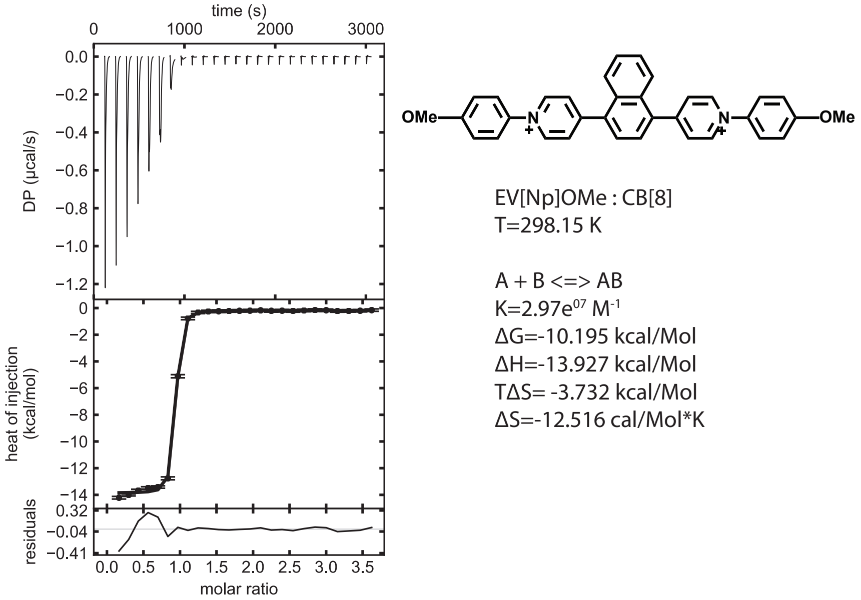

Figure S77: ITC titration of 1 mM EV[Np]OMe to 0.051 mM CB[8] (T=298.15 K, 50 mM sodium acetate buffer pH = 4.75, Cl^- counterions)

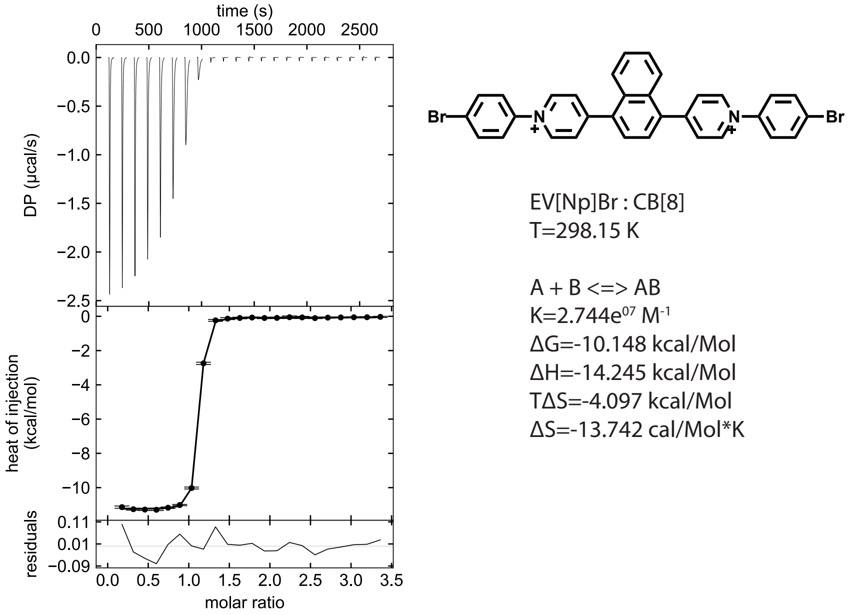

Figure S78: ITC titration of 1 mM EV[Np]Br to 0.051 mM CB[8] (T=298.15 K, 50 mM sodium acetate buffer pH = 4.75, Cl^- counterions)

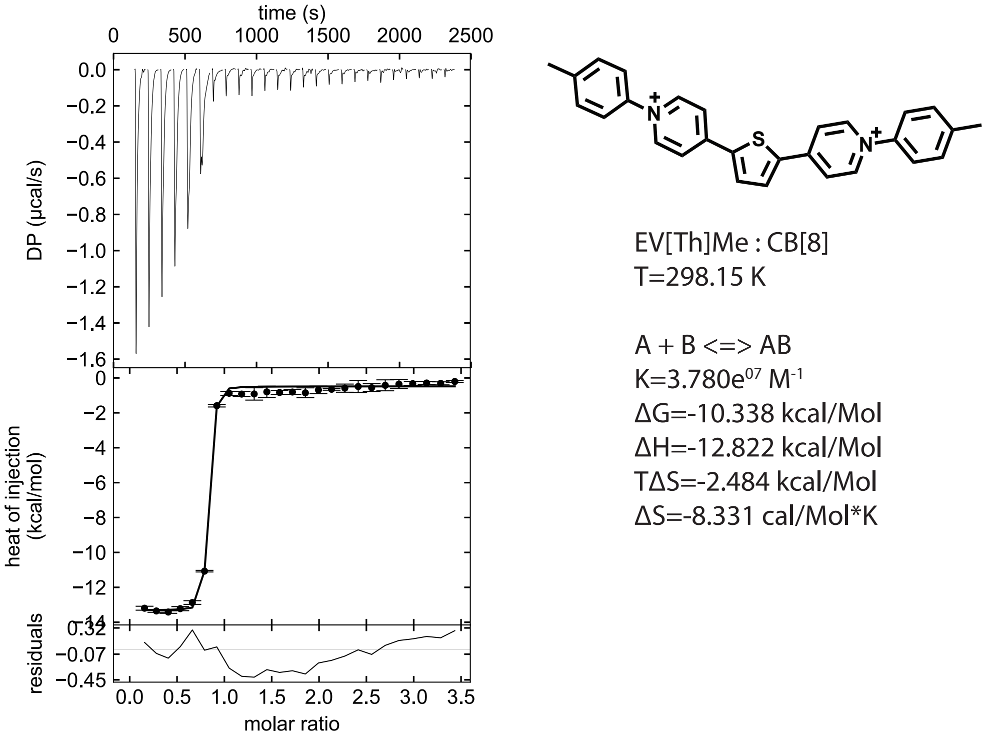

Figure S79: ITC titration of 0.85 mM EV[Th]Me to 0.051 mM CB[8] (T=298.15 K, 50 mM sodium acetate buffer pH = 4.75, Cl^- counterions)

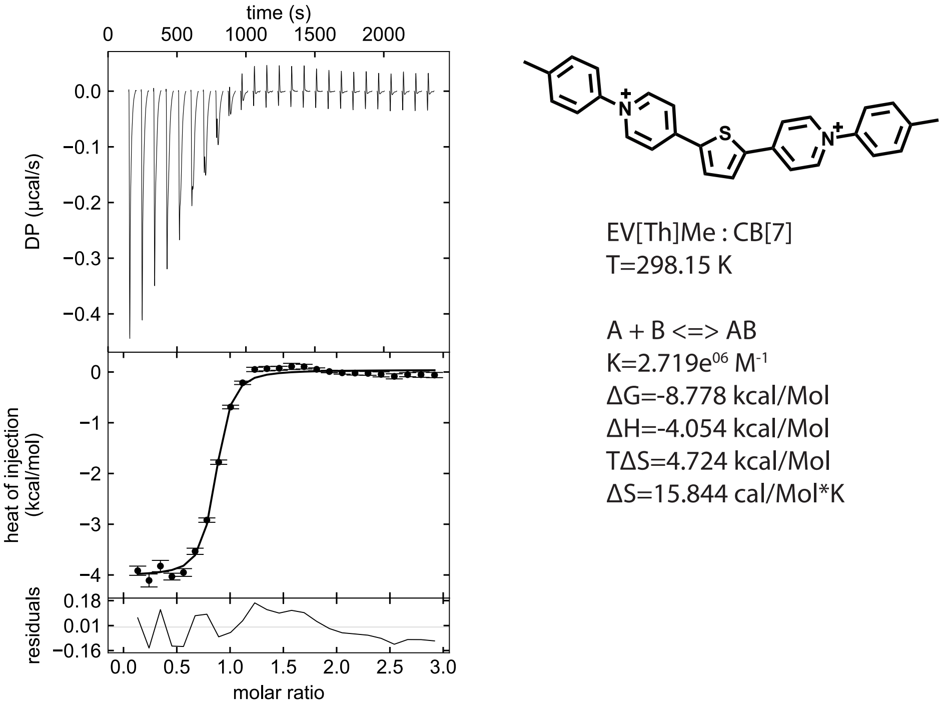

Figure S80: ITC titration of 0.8 mM EV[Th]Me to 0.061 mM CB[7] (T=298.15 K, 50 mM sodium acetate buffer pH = 4.75, Cl^- counterions)

7 Steady-state and time-resolved photophysical characterisation of extended viologens before and after adding CB[8] and CB[7]

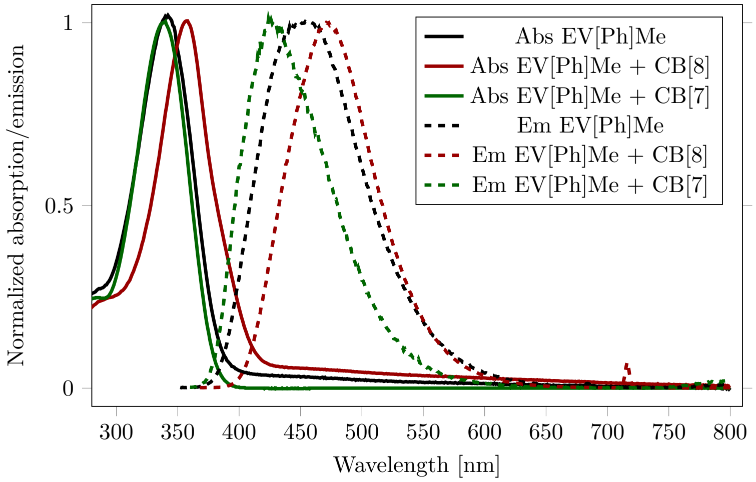

Figure S81: Normalized absorption (solid lines) and fluorescence (dashed lines) spectra of EV[Ph]Me (black line) and its complexes with 1 eq of CB[8] (red lines) and 2 eq of CB[7] (green lines). All measurements were performed in water, 10 µM.

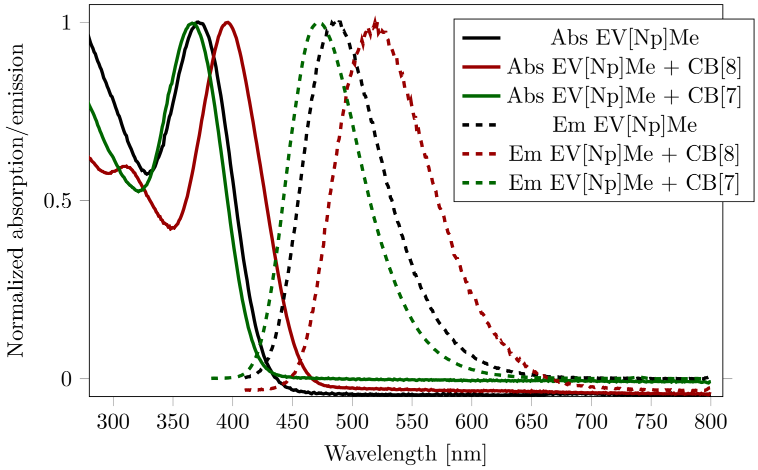

Figure S82: Normalized absorption (solid lines) and fluorescence (dashed lines) spectra of EV[Np]Me (black line) and its complexes with 1 eq of CB[8] (red lines) and 2 eq of CB[7] (green lines). All measurements were performed in water, 10 µM.

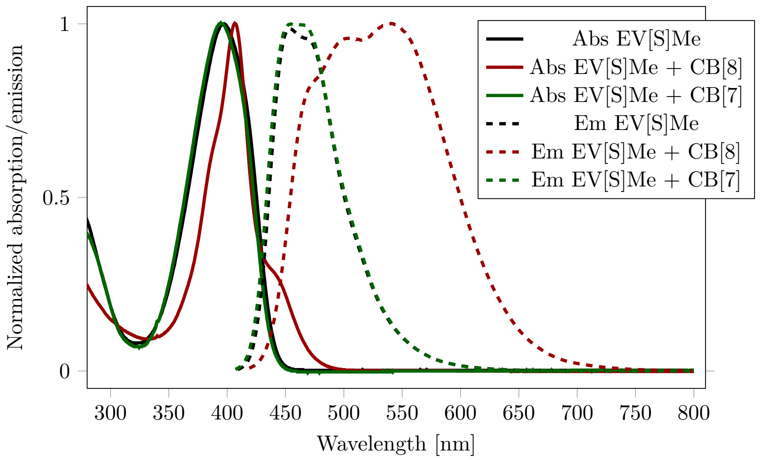

Figure S83: Normalized absorption (solid lines) and fluorescence (dashed lines) spectra of EV[Th]Me (black line) and its complexes with 1 eq of CB[8] (red lines) and 2 eq of CB[7] (green lines). All measurements were performed in water, 10 μM.

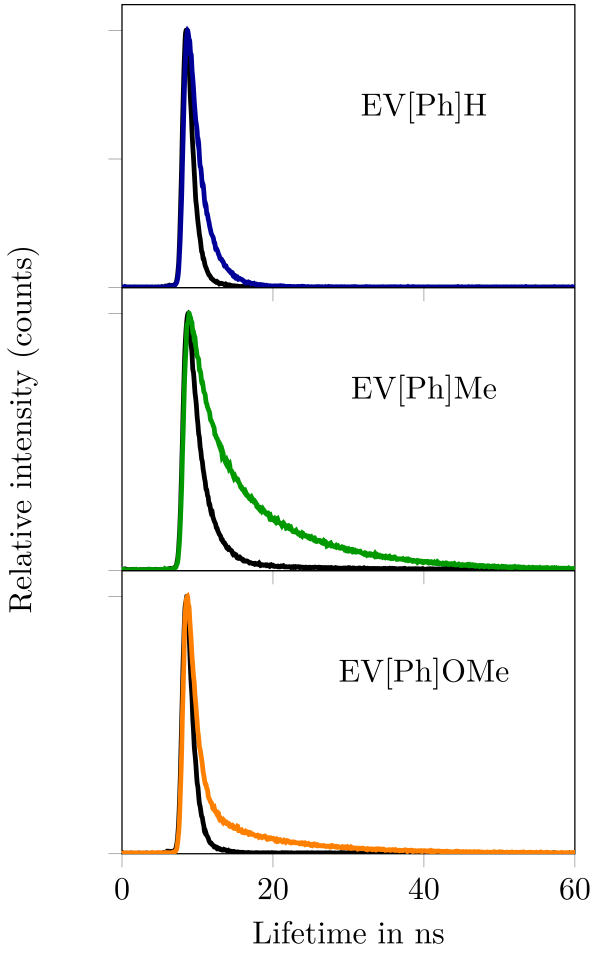

Figure S84: Time-correlated single photon counting decay profiles for EV[Ph]H, EV[Ph]Me, EV[Ph]OMe (black lines) and their 2:2 complexes with CB[8] (coloured lines).

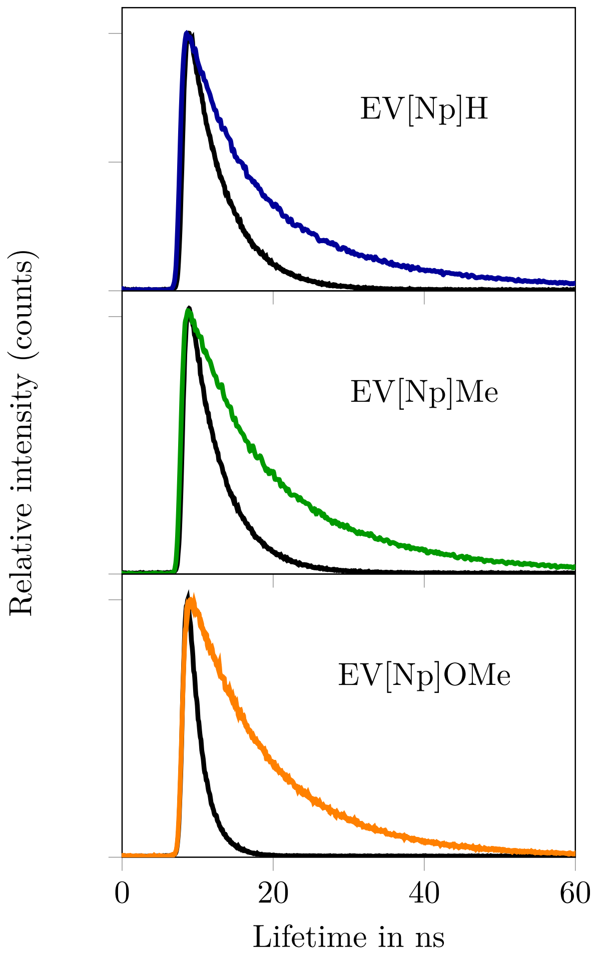

Figure S85: Time-correlated single photon counting decay profiles for EV[Np]H, EV[Np]Me, EV[Np]OMe (black line) and their 2:2 complexes with CB[8] (coloured lines).

Table S3: Steady-state spectral data of EV[X]R in water.

Guest	Host	Host:Guest	MaxAbs (nm)	MaxEm (nm)	Stokes shift (nm)	PLQY	τ_1 (ns)	τ_2 (ns)
EV[Ph]Me	-	-	343	456	113	0.65	1.5	-
EV[Ph]Me	CB[8]	1:1	357	472	115	0.92	2.5 (23%)	10.9 (77%)
EV[Ph]Me	CB[7]	2:1	340	420	80	0.93	1.4	-
EV[Ph]H	-	-	337	404	67	0.84	1.94	-
EV[Ph]H	CB[8]	1:1	350	428	78	0.94	1.75 (97%)	8.89 (3%)
EV[Ph]H	CB[7]	2:1	356	420	64	0.93	1.4	-
EV[Ph]OMe	-	-	348	402	54	0.01	<1	-
EV[Ph]OMe	CB[8]	1:1	374	548	174	0.31	1.14 (51%)	10.33 (49%)
EV[Ph]OMe	CB[7]	2:1	356	420	64	0.93	1.4	
EV[Np]Me	-	-	371	487	116	0.68	4.2	-
EV[Np]Me	CB[8]	1:1	396	518	122	0.58	6.8 (31%)	17.1 (69%)
EV[Np]Me	CB[7]	2:1	366	473	107	0.89	3.2	-
EV[Np]OMe	-	-	373	483	110	0.31	1.82	-
EV[Np]OMe	CB[8]	1:1	402	516	114	0.66	6.23 (20%)	11.98 (80%)
EV[Np]H	-	-	372	485	113	0.28	4.6	-
EV[Np]H	CB[8]	1:1	396	518	122	0.76	6.62	19.01
EV[Th]Me	-		397	453	56	0.62	1.3	-
EV[Th]Me	CB[8]	1:1	407	544	137	0.07	2.7 (82%)	6.7 (18%)
EV[Th]Me	CB[7]	2:1	395	456	61	0.67	1.4	-

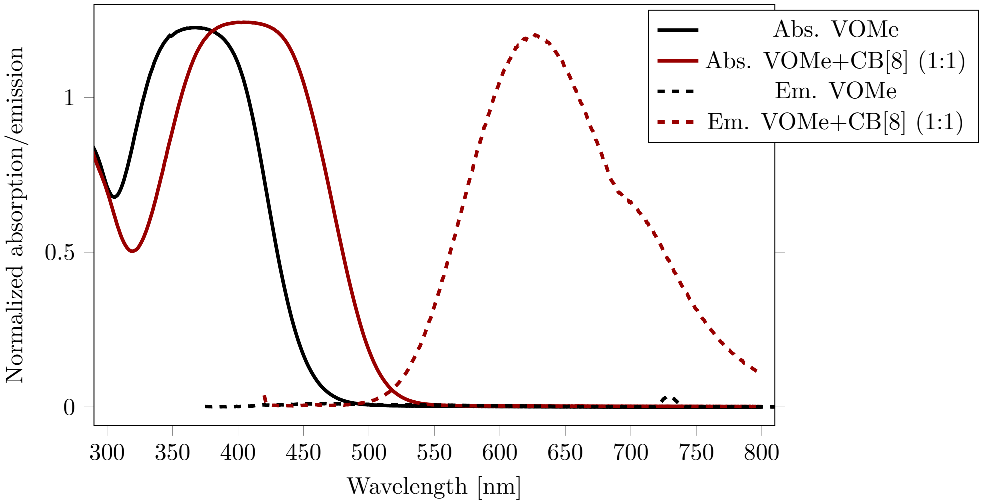

Figure S86: Normalized absorption of the VOMe and VOMe:CB[8] 2:2 complex.

8 Computational details

8.1 Molecular dynamics

Molecular dynamics (MD) simulations were performed with the NAMD 2.12 program[S4] using the generalized Amber force-field and the RESP charge model.[S5] The EV[X]R:CB[8] (where X=Ph, Np, Th) host-guest complexes were solvated in a TIP3P cubic water box with 4000 H_2O molecules using the Packmol software package.[S6] The systems was neutralized by adding 4 Cl ions. For each system, our MD protocol consisted of the following steps: (1) energy minimization over 10000 steps using a timestep of 1 fs; (2) equilibration over 2 ns in the NVT ensemble (T = 298.15 K) with the RMSD of heavy atoms of the host and guest molecules constrained to their initial position using a force constant of 1 kcal·mol^{1}·Å^{2}; (3) 4 ns equilibration run in the NPT ensemble (p = 1.01325 bar, T = 298.15 K) with a timestep of 1 fs; (5) 240 ns production run in the NPT ensemble, where the temperature and pressure were held constant at 298.15 K and 1.0 atm, respectively. Constant temperature was set by a Langevin thermostat with a collision frequency of 1 ps^{1}, whereas the target pressure was reached using the Berendsen barostat. All of the bonds and angles involving hydrogen atoms

were constrained by the SHAKE algorithm.[S7] We used the particle mesh Ewald method[S8] for the long-range electrostatics in combination with a 12 Å cut-off for the evaluation of the non-bonded interactions. Trajectories were run with a time step of 2 fs and the coordinates were printed out in every 500 steps and the last 120 ns used for the analysis.

8.2 TB-DFT calculations

The semiempirical quantum chemical method GFN2-xTB (version 5.9) with the self-consistent D4 dispersion model and implicit water was used for the optimization of EV[X]Me guests and their 2:2 complexes, $CB[8]_2 \cdot (EV[X]Me)_2$. The spectroscopic properties were obtained with sTDA using a spectral range of 12 eV that provide converged spectra for both the EVs and the 2:2 complexes.

Starting geometries

For each system, we explored a range of starting structures. A conformational clustering was applied on the supramolecular complexes based on the conformational sampling obtained in the MD simulations. A total of ten clusters were selected considering clusters defined via RMSDs of all heavy atoms in the guest molecules. For each cluster several representative structures were selected as starting geometries for the TB-DFT optimization.

Geometry optimization

For the $CB[8]_2 \cdot (EV[Ph]Me)_2$ complex, a total of 48 starting structures were used. These complexes converge to structures within an energy range of 6 kcal/mol after geometry optimization. The most stable structures of complexes are similar to each other with the guest molecules oriented in a parallel head-to-head fashion. The structures exhibit a small inter-centroid shift between the guests and have different helicity between guest molecules (Figure S76).

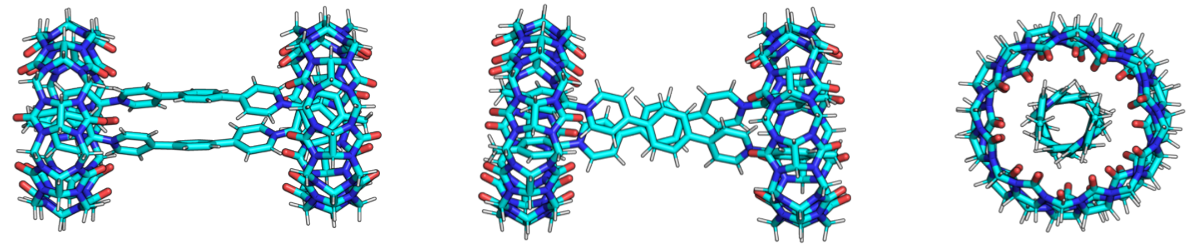

Figure S87: Geometry optimized structures of the EV[Ph]Me in complex with CB[8].

For the $CB[8]_2 \cdot (EV[Np]Me)_2$ complex, a total of 64 starting structures were considered that fall in an energy range of 6 kcal/mol. The most stable structures of complexes are similar to each other in this case as well, with the guest molecules oriented in a parallel head-to-head fashion, exhibiting small shift between guests (Figure S77).

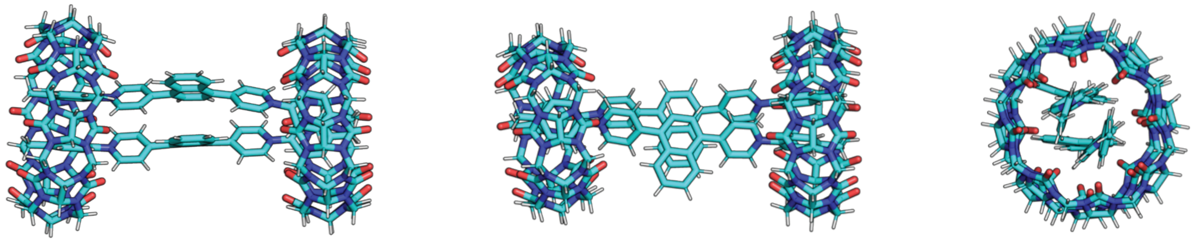

Figure S88: Geometry optimized structures of the EV[Np]Me in complex with CB[8].

For the $CB[8]_2 \cdot (EV[Th]Me)_2$ complex, a total of 64 starting structures were considered within an energy range of 8 kcal/mol. The structures are also structurally similar and have a noticeable difference in helicity and the relative orientation of CB[8] (Figure S78).

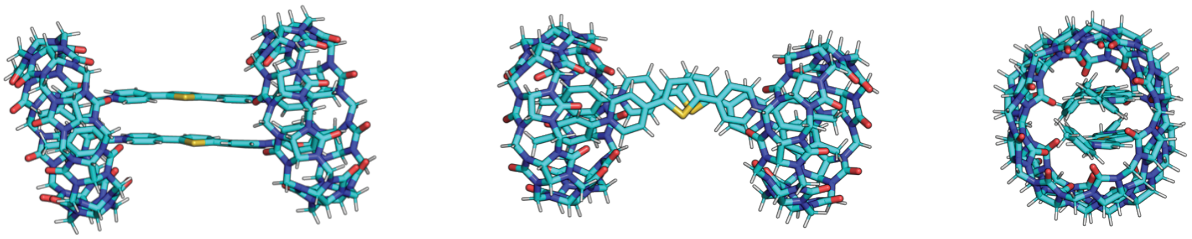

Figure S89: Geometry optimized structures of the EV[Th]Me in complex with CB[8].

The fact that most of the structures converge to similar local minimum for all three

types of complexes indicates the stability of the optimized geometries at the semiempirical level used. To further describe the structural properties of the complexes, we have considered various geometrical parameters shown in Figure 4 and Table S4, that describe the interactions and aggregation of the guests (distance between centroids, angle between interacting rings, intercentroid shift and the average angle between pyridinium motif and the central ring).

Table S4: Geometric parameters for 2:2 complexes of CB[8] and EV[Ph]Me, EV[Np]Me and EV[Th]Me

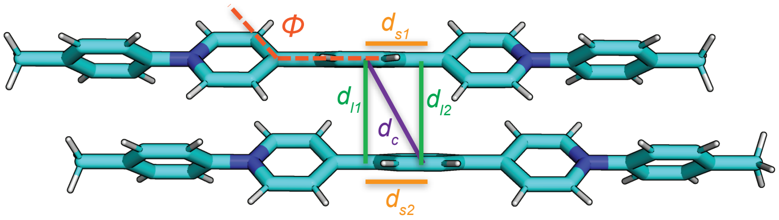

Complex	d_c(Å)**	α (°)	d_{l1} (Å)	d_{l2} (Å)	d_s1 (Å)	d_s2 (Å)	ϕ (°)
EV[Ph]Me: CB[8]	3.38	174.6	3.2979	3.3159	0.7432	0.6582	29.98
EV[Np]Me: CB[8] (1A-2A)*	3.84	166.3	2.9806	3.4451	2.4244	1.701	44.6
EV[Np]Me: CB[8] (1B-2A)*	3.4124	171.17	3.4121	3.3773	0.0458	0.488	
EV[Np]Me: CB[8] (1B-2B)*	3.3128	166.44	3.3159	3.8599	2.7577	1.9238	
EV[Th]Me: CB[8]	3.46	174.9	3.2427	3.2454	1.1991	1.1917	5.7

d_c - Distance between centroids of aromatic rings; α- angle between planes, α =180° for parallel orientation; d_l- distance between planes formed by central rings; d_{s1} and d_{s2} - intercentroid shifts; ϕ - average of four angles between pyridinium motif and the central ring. *There are two other $\pi - \pi$ interaction points that can be found between the central rings in the case of EV[Np]Me (see Figure S79) **We have considered a cut-off of 4 Å in the distance between centroids to include the interaction.

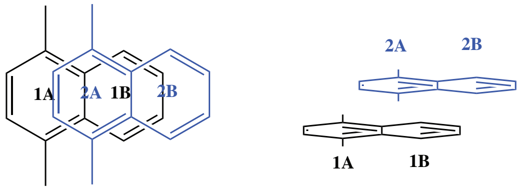

Figure S90: The assignment for the possible $\pi - \pi$ interactions that can be found between the central rings in the case of EV[Np]Me.

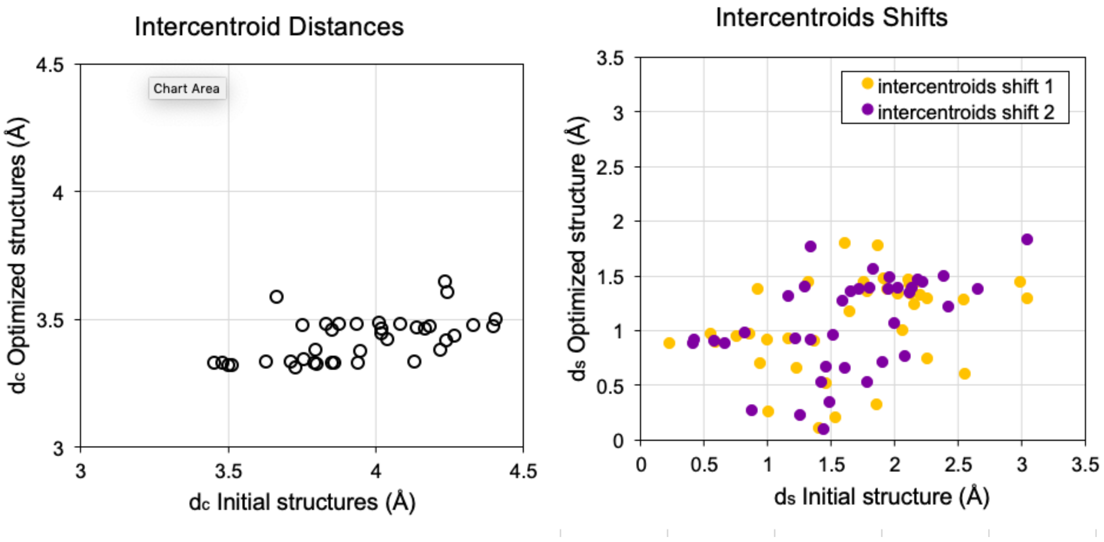

Figure S91: Comparison of d_c and d_s between initial and optimized structure in $CB[8]_2 \cdot (EV[Ph]Me)_2$.

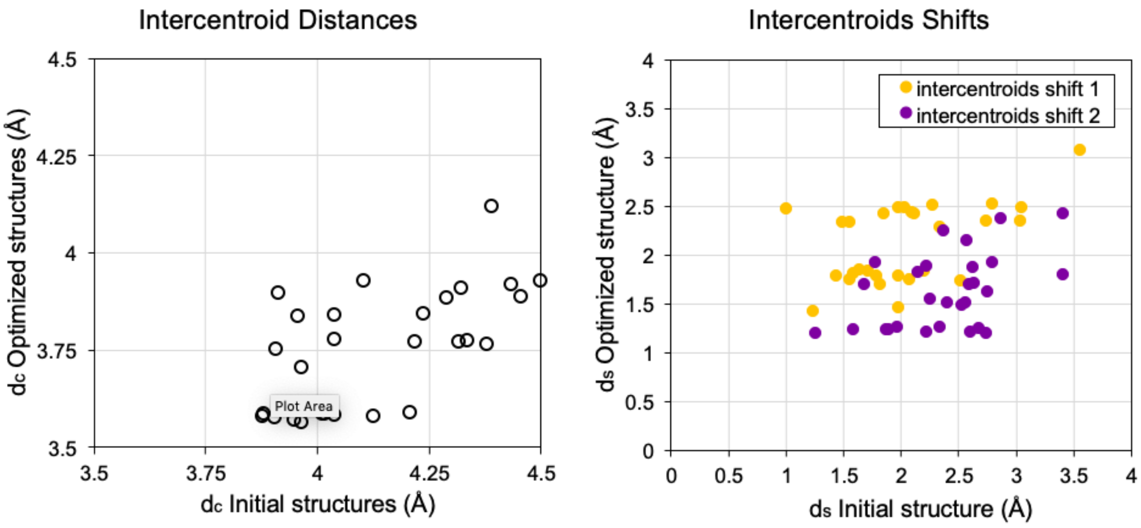

Figure S92: Comparison of d_c and d_s between initial and optimized structure in $CB[8]_2 \cdot (EV[Np]Me)_2$.

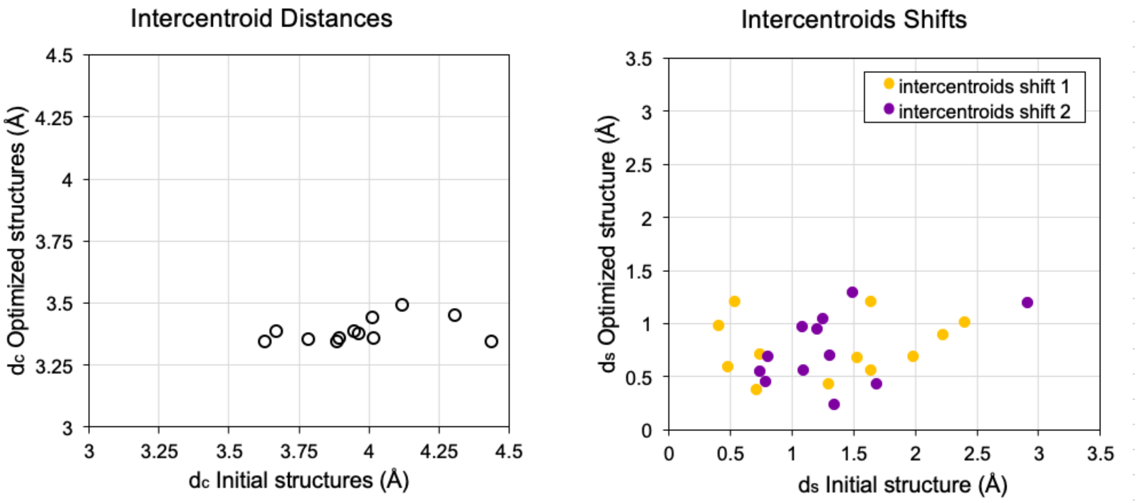

Figure S93: Comparison of d_c and d_s between initial and optimized structure in $CB[8]_2 \cdot (EV[Th]Me)_2$.

Spectroscopic data

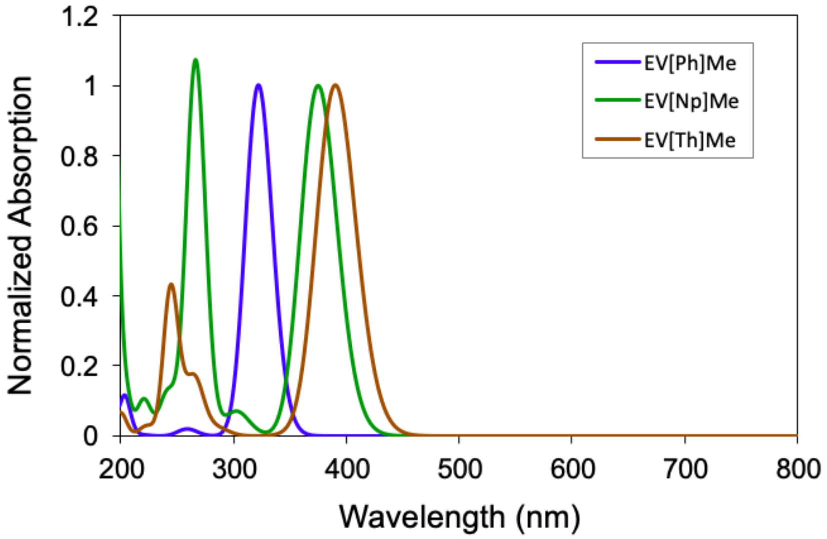

Figure S94: Normalized absorption of monomeric EV[Ph]Me, EV[Np]Me and EV[Th]Me.

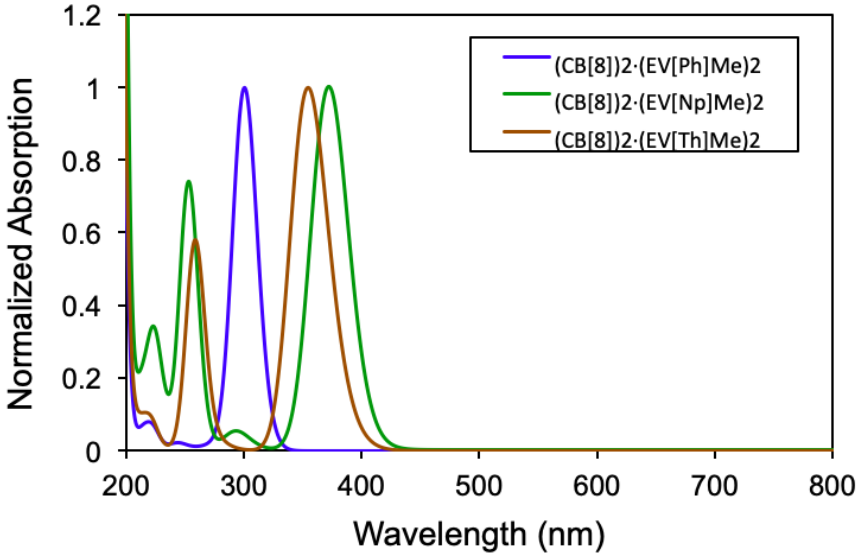

Figure S95: Normalized absorption of the 2:2 CB[8] complexes of EV[Th]Me and EV[Np]Me and EV[Ph]Me.

References

(S1) Day, A.; Arnold, A. P.; Blanch, R. J.; Snushall, B. Controlling Factors in the Synthesis of Cucurbituril and Its Homologues. *J. Org. Chem.* **2001**, *66*, 8094–8100.

(S2) Barnes, J. C.; Juricek, M.; Strutt, N. L.; Frasconi, M.; Sampath, S.; Giesener, M. A.; L, M. P.; Bruns, C. J.; Stern, C. L.; Sarjeant, A. A.; Stoddart, F. J. ExBox: A Polycyclic Aromatic Hydrocarbon Scavenger. *J. Am. Chem. Soc.* **2013**, *135*, 183–192.

(S3) Ryan, S. T. J.; Young, R. M.; Henkelis, J. J.; Hafezi, N.; Vermeulen, N. A.; Hennig, A.; Dale, E. J.; Wu, Y.; Krzyaniak, M. D.; Fox, A.; Nau, W. M.; Wasielewski, M. R.; Stoddart, J. F.; Scherman, O. A. Energy and Electron Transfer Dynamics within a Series of Perylene Diimide/Cyclophane Systems. *J. Am. Chem. Soc.* **2015**, *137*, 15299–15307.

(S4) Phillips, J. C.; Braun, R.; Wang, W.; Gumbart, J.; Tajkhorshid, E.; Villa, E.; Chipot, C.; Skeel, R. D.; Kale, L.; Schulten, K. J. Scalable molecular dynamics with NAMD. *J. Comput. Chem.* **2005**, 1781–1802.

(S5) Case, D. A.; Ben-Shalom, I. Y.; Brozell, S. R.; Cerutti, D. S.; Cheatham III, T. E.; Cruzeiro, V. W. D.; Darden, T. A.; Duke, R. E.; Ghoreishi, D.; Gilson, M. K.; Gohlke, H.; Goetz, A. W.; Greene, D.; Harris, R.; Homeyer, N.; Izadi, S.; Kovalenko, A.; Kurtzman, T.; Lee, T. S.; LEGrand, S.; Li, P.; Lin, C.; Liu, J.; Luchko, T.; Luo, R.; Marmelstein, D. J.; Merz, K. M.; Miao, Y.; Monard, G.; Nguyen, C.; Nguyen, H.; Omelyan, I.; Onufriev, A.; Pan, F.; Qi, R.; Roe, D. R.; Roitberg, A.; Sagui, C.; Schott-Verdugo, S.; Shen, J.; Simmerling, C. L.; Smith, J.; Samolon-Ferrer, R.; Swils, J.; Wa, AMBER 2018. *University of California, San Francisco*

(S6) Martinez, L.; Andrade, R.; Birgin, E. G.; Martinez, J. M. Packmol: A package for building initial configurations for molecular dynamics simulations. *J. Comput. Chem.* **2009**, 2157–2164.

(S7) Andersen, H. C. Rattle: A “velocity” version of the shake algorithm for molecular dynamics calculations. *Journal of Computational Physics* **2009**, 24–34.

(S8) Darden, T.; York, D.; Pedersen, L. Particle mesh Ewald: An Nlog(N) method for Ewald sums in large systems. *J. Chem. Phys.* **1993**, 10089–10092.
